# Diversity of Human-Associated Bifidobacterial Prophage Sequences

**DOI:** 10.3390/microorganisms9122559

**Published:** 2021-12-10

**Authors:** Darren Buckley, Toshitaka Odamaki, Jinzhong Xiao, Jennifer Mahony, Douwe van Sinderen, Francesca Bottacini

**Affiliations:** 1 INFANT Research Centre, University College Cork, Cork, Ireland; darren.buckley@umail.ucc.ie; 2 Next Generation Science Institute, Morinaga Milk Industry Co., Ltd., Zama 252-8583, Japan; t-odamak@morinagamilk.co.jp (T.O.); j_xiao@morinagamilk.co.jp (J.X.); 3 APC Microbiome Ireland, School of Microbiology, University College Cork, Cork, Ireland; J.Mahony@ucc.ie; 4 Biological Sciences, Munster Technological University, Cork, Ireland

**Keywords:** gut microbiota, *Bifidobacterium*, bifidobacteria, phageome, prophage, CRISPR-Cas

## Abstract

Members of *Bifidobacterium* play an important role in the development of the immature gut and are associated with positive long-term health outcomes for their human host. It has previously been shown that intestinal bacteriophages are detected within hours of birth, and that induced prophages constitute a significant source of such gut phages. The gut phageome can be vertically transmitted from mother to newborn and is believed to exert considerable selective pressure on target prokaryotic hosts affecting abundance levels, microbiota composition, and host characteristics. The objective of the current study was to investigate prophage-like elements and predicted CRISPR-Cas viral immune systems present in publicly available, human-associated *Bifidobacterium* genomes. Analysis of 585 fully sequenced bifidobacterial genomes identified 480 prophage-like elements with an occurrence of 0.82 prophages per genome. Interestingly, we also detected the presence of very similar bifidobacterial prophages and corresponding CRISPR spacers across different strains and species, thus providing an initial exploration of the human-associated bifidobacterial phageome. Our analyses show that closely related and likely functional prophages are commonly present across four different species of human-associated *Bifidobacterium*. Further comparative analysis of the CRISPR-Cas spacer arrays against the predicted prophages provided evidence of historical interactions between prophages and different strains at an intra- and inter-species level. Clear evidence of CRISPR-Cas acquired immunity against infection by bifidobacterial prophages across several bifidobacterial strains and species was obtained. Notably, a spacer representing a putative major capsid head protein was found on different genomes representing multiple strains across *B. adolescentis*, *B. breve*, and *B. bifidum*, suggesting that this gene is a preferred target to provide bifidobacterial phage immunity.

## 1. Introduction

The human body harbours trillions of microbial cells whose coordinated actions are believed to be crucial for human health and well-being. The gastrointestinal (GI) compartment contains the highest density of microbes, known collectively as the gut microbiota. Over the course of a human life, the gut microbiota develops and changes in composition, with the formation of complex trophic relationships ranging from symbiosis to parasitism [1,2,3].

Colonisation of the infant gut is a crucial step in the assembly of the gut community and begins immediately after, and perhaps even before, birth via the vertical transfer of microbes from the mother [4]. The development and maturation of the early gut microbiome is a dynamic and orderly process influenced by several environmental and host factors in which positive and negative interactions occur between key members of the gut community [5]. It has been shown that certain species of *Bifidobacterium* or *Lactobacillus* play an important role in the development of the immature gut and are associated with positive implications for long-term host health [6,7]. Conversely, negative features of the gut microbiota such as reduced genetic diversity or aberrant composition, especially with abundant *Bacteroides* or *Ruminococcus*, have been associated with pro-inflammatory states [8,9,10,11,12,13].

Bacteriophages (phages) are viruses that infect prokaryotic hosts and play crucial roles in shaping the composition and diversity of bacterial communities in many environments including the early gut microbiota [14,15]. Prophages are bacteriophages whose genetic material is integrated into the bacterial chromosome and are able to produce phages when activated or induced [16]. It has been shown that intestinal bacteriophages can be detected within hours of birth. Induced prophages, already present within the microbial community, appear to constitute a large source of these intestinal virions [17]. Following birth, the intestinal community experiences successions of drops in phage abundance, followed by periods of “steady-states” [18]. Intestinal phages are believed to exert considerable evolutionary pressure on bacterial hosts affecting abundance levels, microbiota composition, and host characteristics including colonisation, antibiotic resistance, and immune system modulation [19].

The ability of prokaryotes to withstand phage attacks is key for their survival. Prokaryotes have developed numerous resistance mechanisms to fend off phage predation. One of these phage resistance mechanisms is the Clustered Regularly Interspaced Short Palindromic Repeats (CRISPR) system. CRISPR-associated (Cas) proteins play an instrumental role in the survival of prokaryotes against viral invaders by cleaving the DNA of foreign genetic elements. The CRISPR-cas adaptive immune system has been found within 77% of studied bifidobacterial genomes [20] and consists of a genetic locus containing the CRISPRs with their non-repetitive, unique spacer sequences and adjacent genes encoding the Cas proteins [21,22]. It is the spacers that enable the adaptable and sequence-specific inactivating mechanism of the CRISPR system. A spacer is a short segment of sequence that is homologous to phage or other invading DNA sequences and represents a genetic memory of a previous interaction with that sequence. By the acquisition of up to 200 spacers, the prokaryotic host can acquire an expansive viral immune system affecting the efficacy of bacteriophages in exerting evolutionary pressure in their host environment.

However, the dynamics and mechanisms involved in these phage-host interactions are currently poorly described. Therefore, deeper insights into the interplay between the bacterial gut community and their viral predators may allow causal links to be established between microbial dysbiosis and disease, improve current therapeutics, and identify novel targets for microbiome modulating strategies.

In the current study, a comprehensive analysis of predicted bifidobacterial prophages [23] and CRISPR-Cas spacer arrays found among *Bifidobacterium adolescentis*, *Bifidobacterium bifidum*, *Bifidobacterium breve*, and *Bifidobacterium longum* is presented. These species were chosen as the most abundant bifidobacterial species in the human gut. Notably *Bifidobacterium bifidum*, *Bifidobacterium breve*, and *Bifidobacterium longum* are most abundant in early life while *Bifidobacterium adolescentis* becomes more abundant in adulthood. By availing of prophage and CRISPR-Cas prediction software, comparative analysis, and phylogenetic inference, this work represents an overview of the observed diversity among identified prophage-like elements in human-derived bifidobacterial strains, thereby representing a foundation for a better characterisation of the human-associated bifidobacterial phageome.

## 2. Materials and Methods

The assembly information and complete genome sequences of publicly available bifidobacterial strains from *B. adolescentis*, *B. bifidum*, *B. breve*, and *B. longum* species were retrieved from the RefSeq database (https://www.ncbi.nlm.nih.gov/refseq/ (accessed on 1 July 2021)) for analysis.

The whole-genome nucleotidic sequence of the downloaded bifidobacterial strains was used as input for prophage prediction using the PHASTER (PHAge Search Tool Enhanced Release) application programming interface (API) (https://phaster.ca (accessed on 1 July 2021)).

Comparative analysis was performed at both nucleotide and amino acid levels using Average Nucleotide Identity (ANI) analysis, and an all-vs-all BLASTP alignment, respectively, and MCL (Markov Clustering Algorithm) clustering analysis. ANI was assessed at the nucleotide level using Pyani v0.3+ (https://github.com/widdowquinn/pyani (accessed on 1 July 2021). Two-way hierarchical clustering heatmaps were generated from the pyani outputs for graphical representation of intra- and inter-species similarities across the dataset. The predicted prophages underwent comparative analysis using an all-vs-all BLASTP alignment [24] and MCL clustering [25] following a previously described protocol for the analysis of lactococcal prophages [26]. Open Reading Frame (ORF) prediction was performed from nucleotide sequences of the predicted bifidobacterial prophages using Prodigal v2.6.3 [27]. The deduced amino acid sequences of the ORFs were then concatenated and compared in an all-vs-all manner using BLASTP. The all-vs-all BLASTP output file was used as the input for mclblastline to generate clusters of functionally related families. An example script of the comparative analysis is available here: https://github.com/frbot/Comparative-genomics/blob/master/comparative.sh (accessed on 1 July 2021). The obtained binary matrix containing the presence/absence of phage gene families obtained from the mclblastline output was used to generate interactive heatmaps via heatmaply (https://cran.r-project.org/web/packages/heatmaply/vignettes/heatmaply.html (accessed on 1 July 2021)).

Comparative locus maps of seventeen prophages selected from six clusters were generated using the python-based graphical representation program Easyfig v2.2.2 (https://mjsull.github.io/Easyfig/ (accessed on 1 July 2021)). To improve the overall analysis, manual revision of the PHASTER predictions was performed by adjusting the left and right boundaries of predicted prophages. Where appropriate, a prophage’s left- and/or right-ward ends were modified based on the following criteria: (i) presence of a tRNA-encoding gene or a phage integrase located at either the left or right side of the predicted prophage-like element; (ii) consecutive hypothetical proteins containing prophage-like predicted functions assigned to be part of the same prophage region; (iii) putatively co-transcribed, and contiguous Open Reading Frames (of distance < 1 Kb), and encoded within the same DNA strand.

Following Phaster revision, seventeen representative prophages were compared, and their features were classified in functional categories based on the information retrieved from the Virus Orthologous Group (VOG—http://vogdb.org/ (accessed on 1 July 2021)) and visualised.

Intra-group phylogenetic analysis was performed using VICTOR (Virus Classification, and Tree Building Online Resource—https://ggdc.dsmz.de/home.php (accessed on 1 July 2021)). All pairwise comparisons of the predicted phage nucleotide sequences were conducted using the Genome-BLAST Distance Phylogeny (GBDP) method [28] employing settings recommended for prokaryotic viruses [29]. The resulting intergenomic distances were used to infer a balanced minimum evolution tree with branch support via FASTME including SPR postprocessing [30] for each of the formulas D0, D4, and D6, respectively. Branch support was inferred from 100 pseudo-bootstrap replicates each. The obtained trees were rooted at the midpoint and visualised with FigTree http://tree.bio.ed.ac.uk/software/figtree/ (accessed on 1 July 2021). Taxon boundaries at species, genus and family level were estimated with the OPTSIL program [31], the recommended clustering thresholds, and an F value (fraction of links required for cluster fusion) of 0.5 [32].

ViPtree (the Viral Proteomic Tree server—https://www.genome.jp/viptree/ (accessed on 1 July 2021) was used to generate a “*proteomic tree*” of the seventeen predicted prophages and infer their positioning within the viral tree of life.

Identification of CRISPR/Cas systems in bifidobacterial genomes was performed using CRISPRCasFinder [33] (https://crisprcas.i2bc.paris-saclay.fr/CrisprCasFinder/Index (accessed on 1 July 2021)). Non-redundant databases of predicted phages and spacers were created using CD-HIT [34] and used to perform comparative analysis at the nucleotide level in a phage-vs-spacer BLASTN alignment. The nucleotide sequences associated with the BLAST hits were extracted from their parent bifidobacterial genome GenBank file. Prodigal was used to predict the ORFs of the extracted nucleotide sequences, and the compressed archive of the HMMER3 compatible Hidden Markov Models for the pVOG database was used to verify gene identification and functional annotation with the viral protein-specific database.

## 3. Results

### 3.1. Sequence Download and Feature Extraction

In order to compare the genetic features of predicted bifidobacterial prophage-like elements, the GenBank flat files (gff) of all currently available bifidobacterial genomes belonging to the *B. adolescentis*, *B. bifidum*, *B. breve*, and *B. longum* species were downloaded from the RefSeq database (See Section 2). The complete and draft genome sequences of the four above-mentioned species were downloaded for a total of 52 genomes from *B. adolescentis*, 96 from *B. bifidum*, 108 from *B. breve*, and 329 from *B. longum* (of which 288 belong to subsp. *longum* and 41 to subsp. *infantis*). Therefore, the final dataset consisted of 585 bifidobacterial genomes, which were extracted and processed for further analysis. Of note, in the case of the *B. longum* species, two human-associated subspecies have previously been identified (subsp. *infantis* and *longum*). For this study, we did divide *B. longum* into two relevant subspecies *B. longum* subsp. *longum* and *B. longum* subsp. *infantis* to assess possible links with the development and maturation of the early gut microbiome.

### 3.2. Prophage Prediction

PHASTER was employed to identify prophage-like elements in the downloaded bifidobacterial genomes. PHASTER is a prophage prediction tool used for rapid identification and annotation of prophage sequences within bacterial genomes [35]. PHASTER prediction identified 480 prophage-like regions among the 585 bifidobacterial genomes analysed, thus giving an average of 0.82 prophages per human-associated bifidobacterial genome in line with previous literature on *B. breve* [17,36]. The gff files of the identified prophage regions are provided as prophages_all_gff.zip. Overall, 52 presumed prophages were predicted to be resident in the assessed *B. adolescentis* genomes, while 55, 121, 245, and 7 were assigned to be present in *B. bifidum*, *B. breve*, *B. longum* subsp. *longum*, and *B. longum* subsp. *infantis* genomes, respectively (Tables S1–S4). As expected, we observed a high degree of variability in the number of putative prophage regions per genome (ranging from zero to six), and size of the identified regions (ranging from 4.5 Kb to 51.2 Kb with a median size of 14.3 Kb, and a mode of 9.9 Kb). It is noteworthy that many of these smaller prophages are likely to be cryptic, remnant, or fragmented prophages, while the 47 prophage regions predicted to be larger than 30 Kb in size are more likely to represent complete, biologically intact prophages. Overall, the median GC content observed was 60.8%. Specifically, *B. adolescentis* prophages had an average GC content of 59 %, while those residents in *B. bifidum*, *B. breve*, and *B. longum* (representing both subspecies) were shown to possess a GC content of 62 %, 59.8 %, and 61 %, respectively. While evaluating the GC content of the predicted prophages, we confirmed that the observed values are similar to the average GC content of their corresponding bifidobacterial hosts.

We used a simple nomenclature integrating the host species, bacterial strain, and phages numbered in ascending fashion, based on their location within the strain genome. Each species was given an abbreviation. *B. adolescentis* was Bad, *B. bifidum* was Bif, *B. longum* was Blong, and *B. breve* was Bre. For example, BifBIOML-A4ph1 is the first phage that we predicted in the *B. bifidum* strain BIOML-A4.

Strikingly, PHASTER predicted only one presumed complete phage across the whole dataset, encoded within the *B. breve* BR3 genome (Table S3). The manual investigation, and VOG annotation of this prophage, here named *B. breve* BR3 phage 1 (25 Kb), indicated a lack of genes encoding for structural phage proteins, thus suggesting that this phage is unlikely to constitute a complete phage. Furthermore, a manually curated analysis of strains included in this study has previously indicated the presence of functional phages in certain bifidobacterial genomes, but these were predicted as incomplete by PHASTER. This discrepancy highlights one of the main challenges we observed in applying this popular (pro)phage prediction software to bifidobacterial genomes. A high degree of variability and lack of a general consensus between viral sequences, coupled with the underrepresentation of bifidobacterial (and actinobacterial) prophages in the reference database results in ineffective prediction of complete phages. While PHASTER represents a helpful tool to highlight prophage-like regions in bifidobacterial genomes, manual inspection, and experimental investigations are generally required to verify the completeness of the identified phages.

### 3.3. Comparative Analysis

#### 3.3.1. Average Nucleotide Identity

**Figure 1:**
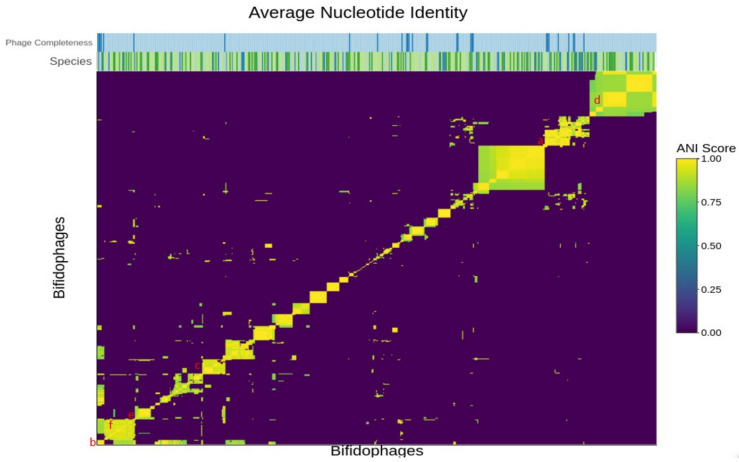
ANI heatmap produced by heatmaply. Yellow boxes in the heatmap signify prophage sequences with high levels of nucleotide similarity to each other. There are clusters of high similarity at both intra- and inter-species levels. The rowside legend indicates the degree of predicted completeness of the predicted prophages. Bifidophages predicted to be likely complete or complete are highlighted in dark blue on the “Completeness” column key. Suspected partial or likely remnant bifidophages are highlighted in light blue. The red letters a to f represent the six clusters highlighted in Figure 4.

**Figure 2:**
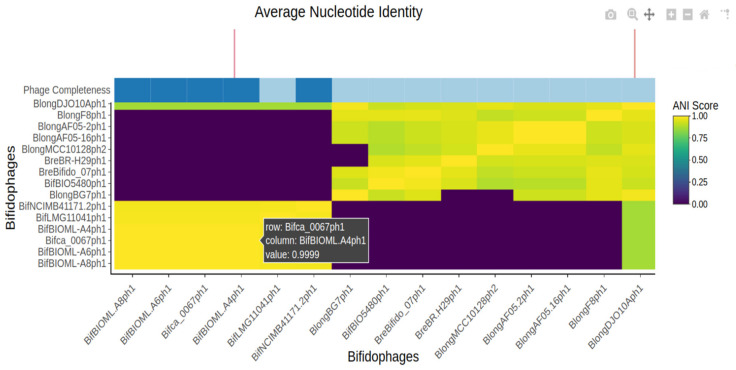
Interactive elements of ANI heatmap produced by heatmaply. Each cell can be highlighted to display the ANI value and the associated bifidophages. Representation of the lowermost left cluster in Figure 1 showing high intra-species ANI of a prophage-like region across several *Bifidobacterium bifidum* strains. Notably, five of the six regions were annotated as “likely complete” prophages by PHASTER.

In order to explore phage diversity at the nucleotide level, ANI was calculated for the identified 480 prophage-like sequences using PYANI. The resulting ANI values were organised in a matrix and visualised in a two-way hierarchical clustering heatmap for graphical representation of intra- and inter-species similarities (Figure 1 and Figure 2). Using a cut off of 0.9 ANI, the analysis identified twenty-four potentially interesting clusters of high ANI including both inter-, and intra-species examples containing an average of twelve representatives per cluster.

#### 3.3.2. Markov Clustering Algorithm Analysis

**Figure 3:**
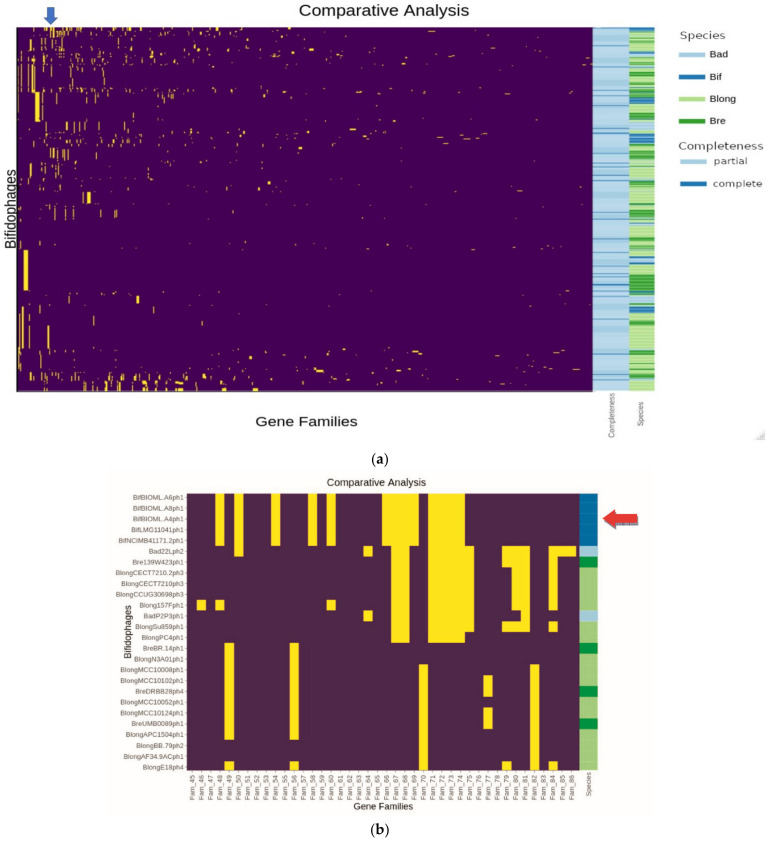
(**a**) Comparative Analysis heatmap produced by heatmaply. Gene families are presented on the horizontal axis, while bifidophages are presented on the vertical axis. We used a binary yes/no 100% gene similarity cut off. Gene familie that are present are represented by a yellow cell, while purple indicates gene absence. Specifically, yellow cells on the same vertical line indicate a gene family present in multiple bifidophages, while yellow cells on the same horizontal line indicates multiple gene families present in a single bifidophage. The rowside legends indicates which species of bidifobacteria the phage belongs to and the degree of completeness predicted by PHASTER. *B. longum* is represented in pale green, *B. breve* in dark green, *B. adolescentis* in pale blue, and *B. bifidum* in dark blue. As previous, bifidophages predicted to be likely complete or complete are highlighted in dark blue and suspected partial or likely remnant bifidophages are highlighted in light blue. A blue arrow highlights an example of a cluster of multiple genes shared between the same *Bifidobacterium bifidum* strains highlighted in Figure 2. A red arrow indicates an example of gene families from a bifidophage shared across multiple *Bifidobacterium* species, highlighted in Figure 2. (**b**) Comparative analysis heatmap highlighting the same cluster represented in Figure 2 indicated here by a red arrow. This image is a zoomed-in view of the area highlighted in Figure 3a. Multiple gene families are shared between the *B. bifidum* phages BifBIOML-a4 phage 1, BifBIOML-a6 phage 1, BifBIOML-a8 phage 1, Bif LMG11041 phage 1 and BifNCIMB41171 phage 1. There is also an example of interspecies gene sharing between phages targeting hosts from *B. adolescentis*, *B. breve*, and *B. longum* directly below the bifidum example. Most notably, Bad22L phage 2, Bre139W423 phage 1 and BlongSu859 phage 1.

To perform a comparative genome analysis at the protein level of the putative prophages, the deduced products of the predicted prophage ORFs were compared in an all-versus-all manner using BLASTP, and MCL clustering to assess the diversity of prophage-like elements and the degree of gene sharing between elements. A heatmap representation of the resulting presence/absence and clustering matrix is indicated in Figure 3a. Further investigation of this heatmap revealed clusters containing predicted prophage-like sequences that were shared across all four assessed species (Figure 3b). Following comparative analysis, the MCL clusters were individually investigated and assessed based on the size and number of prophage-like sequences contained within each cluster, the number of “likely complete” or “complete” representatives, ANI scores, and the degree of gene sharing within the clusters. Six clusters of similar prophage-like sequences were selected for further visualisation with a comparative locus map using Easyfig v2.2.2.

#### 3.3.3. Comparison of ANI and MCL Results

Detailed comparative analysis of the ANI, and MCL heatmaps revealed 14 clusters containing 253 prophages exhibiting a high level of similarity at both ANI and gene sharing levels. These ranged from clusters of 5 to 45 prophages with an average of 10 prophages per cluster. On further investigation, six of these clusters proved to contain similar phage-like sequences with shared integration regions present in genomes of different bifidobacterial strains/species and likely representing complete prophage genomes. For these reasons, they were selected for a more detailed inspection. The remaining eight clusters were deemed to contain less valuable information as despite having shared integration regions these prophage sequences appeared to represent remnant or incomplete phages (as their length was <13 Kb). The large number of likely remnant prophages observed, lacking structural genes or other functional modules (and thus of small size), is in line with previous observations indicating a high degree of genomic decay among prophages within bacterial genomes [37].

**Figure 4:**
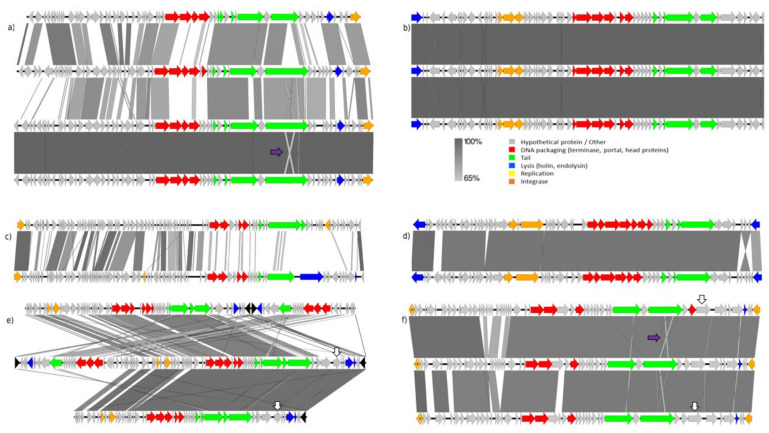
Bifidoprophage genomic comparison and characterisation by Easyfig. Each arrow indicates an open reading frame, orientated in the direction of transcription. The length of arrows is proportional to the the length of the predicted open reading frame. Genes predicted to have similar functions are marked with the same color. Grey arrows indicate hypothetical proteins. Genome architecture and mosaic relationships between the prophage genomes are highlighted with pairwise alignments. The colour spectrum between genomes reflects amino acid identity as shown in the percentage based legend. The prophages in each alignment are described in descending order according to the above representations and are described further in the accompanying text. (**a**) Bre215W447aph1, BreDRBB30ph3, BadTF06-29ph2 and Bad11ph1. (**b**) BifBIOML-A4ph1, BifBIOML-A6ph1 and BifBIOML-A8ph1. (**c**) BlongATCC15697ph1 (from *B. longum* subsp. *infantis*) and BlongTF06-12ACph1 (from *B. longum* subsp. *longum*). (**d**) Bre139W423ph1 and BlongCECT7210ph3. (**e**) Bif85Bph3, BreDRBB30ph2 and BreLMC520ph1. (**f**) BlongAF05-2ph1 (from *B. longum* subsp. *longum*), BreDRBB29ph1 and BreBR3ph5. The purple arrows highlight sequence inversions observed in phage tail-encoding genes. The white arrows highlight reverse transcriptase encoding genes mentioned in Section 3.3.3.

Seventeen predicted prophages were included in the six clusters selected for further analysis. As described in the Section 2, a manual review of the phage boundaries was performed to refine the completeness of the predicted prophages and to improve the comparative analysis. The annotated gff file of the seventeen manually curated prophages is provided as Supplementary File prophage_clusters_gff.zip. For each of the six clusters, prophage alignments were generated (Figure 4), providing insight into the diversity of bifidophages predicted to be present in the genomes of *B. adolescentis*, *B. bifidum*, *B. breve*, and *B. longum*. Interestingly, our analysis showed that closely related, and likely functional, prophages with shared integration points can be found among all four species.

A high degree of mosaicism in bifidophage genomes was observed, in keeping with previous observations, indicating that events of integration and gene shuffling commonly occur in these elements [17,38,39,40]. Accurate detection of phage genes required the aid of pVOG predictions. Annotation of phage genes is difficult due to the lack of reference sequences in the databases. Notably, genes predicted to encode structural components of phage particles were always predicted to be present and were conserved across prophages (Figure 4). Structural genes encoding phage capsid and tail were found to be located in close proximity to one another, indicating that co-location of functionally-related phage genes is a feature of the bifidobacterial prophages studied. Co-location of functionally-related phage genes has previously been described in other phage families [26,41].

Diversity-generating retroelements (DGRs) are genetic cassettes that selectively mutate target genes to produce hypervariable proteins. First characterised in a group of *Bordetella* species, bacteriophage BPP-1 is predicted to possess a DGR associated with a hypervariable tail fiber-encoding gene, which together would be expected to enable host tropism switching [42,43]. We found evidence of a gene encoding a reverse transcriptase located in close proximity of phage tail structural genes across two clusters representative of potentially active prophages. In the cluster represented by Figure 4e, a predicted reverse transcriptase-encoding gene is positioned in close proximity to a phage tail protein-encoding gene present in BreDRBB30ph2 in *B. breve* DRBB30 and BreLMC520ph1 in *B. breve* LMC520 (indicated by a vertical white arrow in Figure 4e). In a second cluster represented in Figure 4f, a reverse transcriptase-encoding gene is located in close proximity to a tail protein-encoding gene in prophage genomes BlongAF05-2ph1, BreBR3ph5, and BreDRBB29ph1, identified in the genomes of *B. longum* AF05-2, *B. breve* BR3, and *B. breve* DRBB29, respectively (also indicated by a vertical white arrow in this figure). These may introduce increased sequence divergence between the targeted tail gene and the rest of the genome when compared to this genome region in related phages. An almost identical tail protein-encoding gene is present in both BlongAF05-2ph1 and BreDRBB29ph1 with evidence of sequence shuffling observed in the sequence alignment and occurring in the tail protein-encoded gene by BreBR3ph5, indicating a change in the target for this phage.

Occasionally, we identified genes encoding elements of the DNA replication machinery, but these were not always present (or likely not detected). This may be due to a lack of homologous genes in the reference database. Complete bifidophages are often integrated in the proximity of tRNA-encoding genes [23] and were always found to harbour an integrase gene (at either left or right border of the prophage genome), which is involved in the integration or excision of the prophage in or out of the host chromosome.

All possibly complete and active bifidophages predicted by our analysis show a modular organisation of their genes, based on gene function, resembling that of lambdoid phages [37] and previously described *Bifidobacterium* prophages [17,44]. These modules can functionally be grouped into integration/excision, lysis, DNA replication, tail/head morphogenesis, and DNA packaging. We have observed that phage integrase-encoding genes are often located at the left or right boundary of predicted prophage-like elements. The products of these integrase genes mediate the integration/excision of the phage into/out of the host chromosome during lysogenic/lytic cycle switching and promote site-specific recombination between phage and host DNA recognition sites. These integrase modules were often found close to genes predicted to be involved in lysis as previously described in bifidophages [44]. During the lytic phase of most bacteriophages, a phage-encoded peptidoglycan-degrading activity is activated. Holin proteins provide access to the peptidoglycan layer for endolysins by creating small holes in the membrane. Endolysins are enzymes that degrade peptidoglycan and were always identified as single proteins in our predicted elements. Genes involved in tail/head morphogenesis follow a modular organisation and are always co-located in clusters within the identified prophage-like elements. Notably, genes encoding DNA packaging proteins, capsid components, and tail fibers were found to be located in close proximity.

#### 3.3.4. Comparative Analysis of Seventeen Identified Prophages

The combination of ANI and MCL clustering analysis allowed the identification of six MCL clusters encompassing seventeen prophages, which are commonly present among four *Bifidobacterium* species.

Figure 4a shows a pairwise alignment, which represents an interesting example of an identical prophage shared between two non-identical hosts *B. breve* 215W447a and *B. breve* DRBB30. There is a close relationship with two other prophages identified in *B. adolescentis* TF06-29 and *B. adolescentis* 1-11. Therefore, this cluster represents a good candidate for presumed inter- and intra-species prophage transmission. Of note, apparent DNA inversion, insertion, and deletion events are observed in one of the tail-associated genes (Figure 4) across *B. breve* and *B. adolescentis*, suggesting that these prophages represent potentially active phages with different host specificities.

Figure 4b contains three predicted prophages in *B. bifidum* BIOML-A4, BIOML-A6, and BIOML-A8. These prophage genomes are nearly identical with no major genetic differences or reshuffling observed between them. All three prophages are located at different loci within the genomes. Of note, these prophages lack a gene with similarity to genes encoding cell lytic functions. We cannot exclude that one of the hypothetical proteins encoded in this prophage is involved in lysis, and this may be due to a lack of homologous genes in the reference database.

Figure 4c shows two predicted prophages in *B. longum* subsp. (*B. longum* subsp. *infantis* ATCC15697 and *B. longum* TF06-12AC strains) with a high degree of mosaicism. The left half of the prophage region is highly homologous, but DNA integrations have promoted genomic insertions on the second half of the genome. This is a prime example of the high level of genomic diversity that can be observed across bifidobacterial prophages even within the same species.

Figure 4d represents an example of a nearly identical phage that is common between *B. breve* 139W423 and *B. longum* subsp. *infantis* CECT7210. This prophage harbours several gene encoding functions involved in DNA packaging, capsid, tail fiber, and DNA replication. These give reason to believe that this phage may be active and target representative strains of both *B. breve* and *B. longum* species.

In Figure 4e a typical example of closely related prophages was observed among strains *B*. *bifidum* 85B, *B*. *breve* DRBB30, and *B*. *breve* LMC520. With a degree of mosaicism, it appears that two distinct prophages (identified by two sets of DNA packaging and tail genes) have integrated side by side in both *B*. *bifidum* 85B and *B*. *breve* DRBB30, thus suggesting a common integration site. All three strains carry a highly homologous prophage showing gene identity above 90%. Our comparative analysis indicates that prophage regions are highly prone to genomic inversions and reshuffling, especially in phage tail-associated regions.

Figure 4f represents a set of highly homologous prophage-like sequences in *B. longum* and *B. breve*, designated BlongAF05-2ph1, BreDRBB29ph1 and BreBR3ph5, showing evidence of deletions and gene shuffling. As described above, a reverse transcriptase encoding gene is found in close proximity to a phage tail protein-encoding gene in all three prophages likely representing a diversity generating retroelement not yet described in *Bifidobacterium* (reverse transcriptase encoding gene highlighted by a vertical white arrow and tail-associated inversion indicated by a purple arrow in Figure 4f).

### 3.4. Phylogenetic Analysis

**Figure 5:**
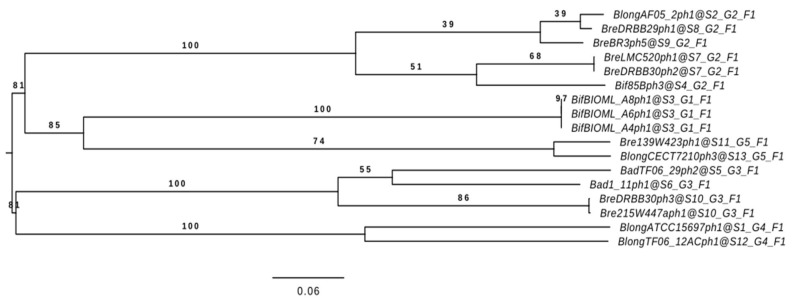
Intra-group Phylogenetic Tree created by VICTOR. Phylogenetic tree obtained using the prophage genomes of the seventeen prophages included in the six clusters of interest. The bar scale indicates phylogenetic distances. Bootstrap values are reported for a total of 100 replicates.

To investigate the phylogenetic relationships of the seventeen selected prophages, two approaches were used. The seventeen nucleotide sequences were compared to each other using VICTOR for intra-group phylogenetic analysis. This process generated a phylogenetic tree which was used to infer the evolutionary relationships between these predicted prophages. Prophages identified by ANI to be present between strains and species have been found to be phylogenetically related (Figure 5). The clusters identified in Section 3.3. are supported by this analysis, thus indicating that the relationships and similarities highlighted by our comparative genome analysis are also supported at a phylogenetic level.

**Figure 6:**
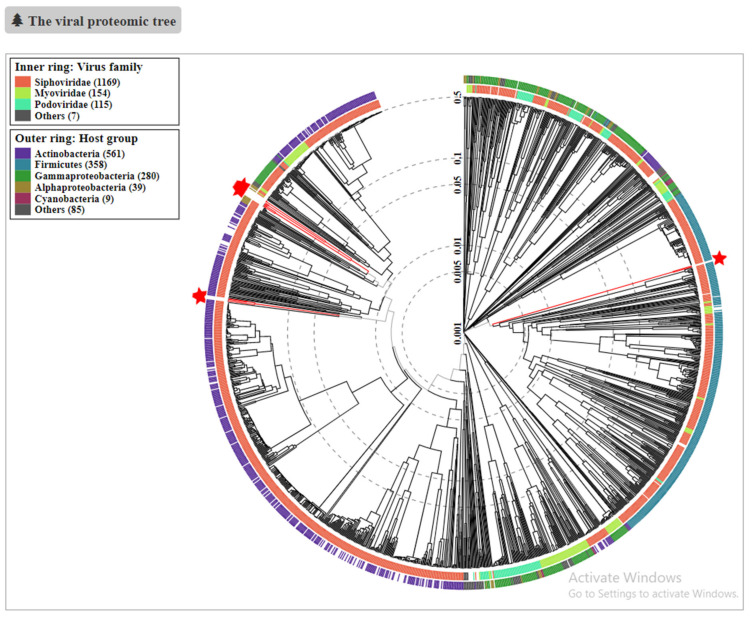
ViPtree circular proteomic tree of related dsDNA viruses with prokaryotic hosts. Submitted prophage sequences are highlighted with red stars.

A multifasta file containing all seventeen predicted prophage genomes was assessed by ViPtree for a global phylogenetic analysis against a viral reference database. To determine the phylogenetic positioning of the 17 selected bifidobacterial prophages within the proteomic tree of reference prokaryotic dsDNA viral sequences, a nucleotide multifasta file containing all seventeen predicted prophages was submitted to the ViPtree analysis (see Section 2). As viral groups identified in a proteomic tree approach correspond well to established viral taxonomies [45], we decided to use this resource to establish the nearest neighbours of our prophage-like elements in existing databases. Gene prediction on the viral genomes and a protein similarity search against the NCBI non-redundant database were performed in parallel with the proteomic tree construction. This tool generated a global phylogenetic analysis of our query sequences against the database of double-stranded DNA viruses known to be associated with prokaryotic hosts containing 2687 viruses. Notably, there are no *Bifidobacterium*-associated viruses in the reference database, which is an indication of the underrepresentation of bifidophages in currently available tools. As scores of zero are excluded from the resulting proteomic tree, this computation resulted in a final tree of 1490 sequences. The assessed *Bifidobacterium* prophages are highlighted by a red star in the generated tree presented in Figure 6.

The predicted prophages do not cluster in the same clade and are unlikely to constitute a *Bifidobacterium*-specific family. Though residing in very related hosts, these prophages appear to be distinctly different from each other. This result represents another clear indication that there is a high degree of genetic diversity observed across bifidophages, which also justifies the need for collecting a higher number of representative and manually-curated viral sequences for this genus. The high level of mosaicism displayed by these prophage-like elements may be explained by multiple module exchange events between phages infecting Firmicutes and Actinobacteria. Such events could have been facilitated by the common ecological origin of members of these two phyla [44]. However, we also noticed that our 17 prophages generally cluster with viruses of actinobacterial hosts.

As shown in Figure 6, the prophages represented by Figure 4a, highlighted by the inferior left red star, are similar to each other, and to other bifidobacteria-associated phages. They are also distantly related to *Microbacterium*, and *Gordonia* phages, which target actinobacterial hosts.

Furthermore, the prophages represented in Figure 4c, highlighted by the right red star, are the only example of bifidobacterial prophages closely located with mainly Firmicutes-infecting phages. They are similar to each other but much less similar to the other bifidophages and are even less similar to the other phages analysed.

Finally, Figure 6 shows the third and largest phylogenetic clade identified by ViPtree represented by the superior left red star containing prophages from the remaining clusters. They are similar to each other but not to other bifidobacteria-associated phages. They are more related to *Streptomyces* and *Mycobacterium* infecting prophages, all having Actinobacteria as prokaryotic host. These findings once again highlight the lack of bifidobacterial prophages in the reference databases.

### 3.5. CRISPR-Cas Prediction

In order to identify the CRISPR-Cas spacer arrays, the software tool CRISPRCasFinder was employed (see Section 2). The CRISPRCasFinder program enables easy detection of CRISPRs and Cas genes in sequence data based on the search of protein similarity using Hidden Markov Models (HMMs) and a genetic model of the identified components. CRISPRCasFinder analysis provides summary information on CRISPR arrays and *cas* gene clusters in the order in which they are positioned along the sequence and details on individual CRISPR arrays and *cas* gene clusters.

CRISPRCasFinder prediction identified 38,676 protospacers among the 585 whole genomes analysed. Further analysis using CD-HIT identified 14,501 non-redundant spacers, thus giving an average of 24.7 unique spacers per human-associated bifidobacterial genome. Overall, 1513 spacers were predicted to belong to *B. adolescentis*, while 883 spacers were assigned to *B. bifidum*, 2895 to *B. breve*, and 9210 to *B. longum* strains. The non-repetitive spacers were extracted from these predictions and processed for further investigation.

### 3.6. Comparison of Phage and Spacer Sequences

In order to compare the predicted prophage and spacer nucleotide sequences, the spacer arrays were mapped onto the prophage sequences to assess if they target any identified prophages. BLAST alignments of predicted spacer arrays against prophages identified 1488 significant hits and 638 unique sequences using an e-value of 0.01 and 98% identity as cut-off values for significance (Table S7). These unique sequences likely represent prophage genes and validate our prediction that these sequences represent parts of invading genetic elements. The larger number of hits indicates that these sequences are conserved across different bifidophages and may be preferential targets for the CRISPR/Cas system in bifidobacteria.

Functional assignment of the genes was confirmed using pVOG database and HMMER3 revealing 20 hits across 15 different genes (Table S5). Less than 3% of the unique sequences had corresponding information in the pVOG database, once again highlighting the lack of bifidobacterial viral information currently present in this database.

Of those with information, the majority were classified as hypothetical proteins in REFSEQ database. Of note, 13 of the 20 hits targeted likely complete prophage sequences, while 4 of the 20 hits targeted remnant or partial phage sequences containing some but not all of the required modular genetic elements. The remaining three hits are unlikely to hit prophage sequences, however, one sequence was associated with protein A6, a protein essential for maturation of an immature virion, and another was associated with a probable diguanylate cyclase DgcQ. Most notably, a spacer representing a putative major capsid head protein was found on multiple strains across *B. adolescentis*, *B. breve*, and *B. bifidum*, thus suggesting that this gene may be a preferred target for bifidobacterial phage immunity (Table S6).

## 4. Discussion

### 4.1. Prophage Prediction

Due to the inherent diversity, and lack of a universal marker among viral genomes, prophage prediction is a challenging task. PHASTER was chosen as a putative phage search tool due to its accessible application programming interface and competitive sensitivity scores in benchmarking tests on gold standard genomes [46,47]. PHASTER proved adequate in predicting prophage-like regions within bifidobacterial genomes, but manual investigation of these regions is required to verify predictions.

We found the PHASTER predictions of bifidobacterial prophages to have inadequate sensitivity. PHASTER only predicted one phage to be intact/complete, and on manual examination, the identified genomic region of this prophage does not contain genes associated with key structural phage proteins. Conversely, PHASTER classified prophages previously identified to be complete, as incomplete prophage fragments. This is likely due to a lack of high-quality information associated with bifidobacterial genomes, or close relatives, in the reference database. To attenuate this issue for future studies, we intend to develop a curated database of predicted and, where relevant, functionally characterised bifidobacterial prophage sequences to supplement relevant viral reference databases.

The PHASTER completeness scoring algorithm relies heavily on phage-related keywords being present in the submitted Genbank files. Bifidobacterial prophages are a novel area of research, highlighted by the lack of a single *Bifidobacterium*-associated viral nucleotide sequence in the Virus-Host database featuring manual annotation of viral sequences covering RefSeq, Genbank, Uniprot, and Viral Zone. Furthermore, only two *Bifidobacterium* phages are included in the Virus Orthologous Group database, used in this project for gene annotation. These findings underscore the predictive weaknesses exposed in this study and explain the motivation for a high-quality reference database of *Bifidobacterium*-associated phages. To attenuate this issue for future research, we will develop and submit a curated database of predicted bifidobacterial prophage sequences to supplement viral reference databases.

### 4.2. Assumptions and Limitations

During our analysis, we made several assumptions due to a lack of high-quality information in this area. We assumed that phages under a certain size are more likely to represent cryptic or remnant phages (<13 Kb), and that phage genomes larger than a certain size are more likely to encompass complete or active phages when compared to shorter phages (>20 Kb). These assumptions are based on studies involving likely unrelated phages and may not be suitable for extrapolation to this area. Furthermore, comprehensive studies investigating bifidobacterial phages will need to test these assumptions by manually reviewing and functionally characterising a larger selection of predicted phages.

As this is an in silico analysis, the obtained findings were not validated by experimentation of host–phage interactions in a recreated gut environment to verify phage activity. Nonetheless, our comparative study allowed us to identify a high level of genomic diversity in *Bifidobacterium*-associated phages, some of which appear to be capable of infecting multiple species. Future research efforts will be needed to experimentally verify our findings of an available biobank in a fashion similar to previous work [39]. However, a manual review of the selected prophage sequences was performed, and all prophages involved in the five clusters contained the key genes required for prophage activity, thus suggesting they may be functional. These findings lend further weight to the argument for further research in this area.

### 4.3. Bifidobacterial Prophage Elements

The objective of this study was successfully achieved. Our analysis represents an investigation of the diversity of viral elements in bifidobacterial species, showing that either similar or highly diverse prophages can be found between species and strains. This study provides compelling evidence showing that closely related, and likely functional, prophages can be found shared across all four human-associated *Bifidobacterium* species analysed. This novel finding positions bifidobacterial phages as a strong candidate for further investigation on a larger scale.

Analysis of the CRISPR-Cas protospacers provided evidence of historical interactions between bifidobacterial species and strains. Despite a large number of prophage–protospacer hits, most of the sequences did not have accompanying pVOG annotation, once again highlighting the shortage of available bifidobacterial gene information. Most notably, a spacer representing a putative major capsid head protein was found on multiple strains, thus suggesting that this gene may be a preferred target for bifidobacterial phage immunity.

Further research would involve metagenomic analysis of new samples to allow the identification of potentially active phages and follow-up analysis of selected samples in a recreated gut environment to verify host–phage interactions. Increased knowledge of host-phage interactions may lead to novel targets for microbiota-modulating therapies.

## Data Availability

Data supporting the reported results can be found in the Supplementary Materials.

## Supplementary Materials

The following are available online at https://www.mdpi.com/article/10.3390/microorganisms9122559/s1, Table S1: Predicted prophages of *Bifidobacterium adolescentis*, Table S2: Predicted prophages of *Bifidobacterium bifidum*, Table S3: Predicted prophages of *Bifidobacterium breve*, Table S4: (a) Predicted prophages of *Bifidobacterium longum* subsp. *longum*, (b) Predicted Prophages of *Bifidobacterium longum* subsp. *infantis*, Table S5: Results table of pVOG HMMER3 queries against the phage-protospacer hits, Table S6: Annotation of pVOG genes associated with phage-protospacer hits, Table S7: BLAST alignments of predicted spacer arrays against predicted prophages.
