# Supplementary material for: Diversity of Human-Associated Bifidobacterial Prophage Sequences
Source: Microorganisms. 2021 Dec 10;9(12):2559. doi: 10.3390/microorganisms9122559 (PMC8705816; doi:10.3390/microorganisms9122559)
Supplement: Supplementary file 1 [file microorganisms-09-02559-s001.zip › microorganisms-1449866-SI.pdf]

microorganisms

Article

Diversity of Human-Associated Bifidobacterial Prophage Sequences

Darren Buckley [1], Toshitaka Odamaki [2], Jinzhong Xiao [2], Jennifer Mahony [3], Douwe van Sinderen [3,*] and Francesca Bottacini [3,4,*]

1 INFANT Research Centre, University College Cork, Cork, Ireland; darren.buckley@umail.ucc.ie
2 Next Generation Science Institute, Morinaga Milk Industry Co., Ltd., Zama 252-8583, Japan; t-odamak@morinagamilk.co.jp (T.O.); j_xiao@morinagamilk.co.jp (J.X.)
3 APC Microbiome Ireland and School of Microbiology, University College Cork, Cork, Ireland; J.Mahony@ucc.ie
4 Biological Sciences, Munster Technological University, Cork, Ireland
* Correspondence: d.vansinderen@ucc.ie (D.v.S.); francesca.bottacini@mtu.ie (F.B.)

Table S1. Predicted Prophages of *Bifidobacterium adolescentis*.

Phage Name	Location	Completeness Score	Size	GC Content
BadATCC15703ph1	1723406-1730671	incomplete(60)	7.2Kb	54.9
BadL2-32ph1	1097675-1103808	incomplete(10)	6.1Kb	60.5
Bad22Lph1	792315-802319	incomplete(30)	10Kb	58.5
Bad22Lph2	1492335-1532197	incomplete(60)	39.8Kb	61.3
BadBB23ph1	1755699-1775279	questionable(80)	19.5Kb	52.2
BadBB23ph2	1843106-1861999	incomplete(20)	18.8Kb	62.5
Bad42Bph1	1692963-1699748	incomplete(10)	6.7Kb	62
Bad487Bph1	947580-960946	incomplete(10)	13.3Kb	58.3
Bad487Bph2	951934-982860	questionable(90)	30.9Kb	59.7
Bad487Bph3	1192083-1225169	incomplete(50)	33Kb	57.6
Bad487Bph4	1247614-1256640	incomplete(10)	9Kb	56.9
BadAL46-7ph1	822128-832132	incomplete(30)	10Kb	58.5
BadLMG10733ph1	757185-765725	incomplete(30)	8.5Kb	60.6
BadLMG11579ph1	771811-780352	incomplete(30)	8.5Kb	60.6
BadLMG11579ph2	957029-965424	incomplete(10)	8.3Kb	60
Bad1-11ph1	822158-866333	incomplete(50)	44.1Kb	57.8
BadTF06-2ACph1	5583-43708	incomplete(40)	38.1Kb	57.6
BadTF06-29ph1	673368-683371	incomplete(30)	10Kb	58.5
BadTF06-29ph2	740762-791335	incomplete(40)	50.5Kb	58.1
BadTF06-29ph3	940317-951590	incomplete(20)	11.2Kb	60.4
BadTF06-10ACph1	324114-364449	incomplete(40)	40.3Kb	57.5
BadTF06-10ACph2	418095-456221	incomplete(40)	38.1Kb	57.6
BadAF28-4ACph1	793018-803079	incomplete(30)	10Kb	58.4
BadAF21-27ph1	723769-759770	incomplete(60)	36Kb	60.3
BadOF04-5ph1	792601-802605	incomplete(30)	10Kb	58.5
BadOF04-9ACph1	792607-802668	incomplete(30)	10Kb	58.4
BadAF15-3ph1	649904-658444	incomplete(30)	8.5Kb	60.4
BadAF15-3ph2	796362-803874	incomplete(20)	7.5Kb	61.2
BadAF15-3ph3	860716-869111	incomplete(10)	8.3Kb	60
BadTM06-51ph1	684703-693243	incomplete(30)	8.5Kb	60.4
BadAM41-17ph1	795232-803772	incomplete(30)	8.5Kb	60.5
BadAM36-3ACph1	632904-641444	incomplete(30)	8.5Kb	60.4

Phage Name	Location	Completeness Score	Size	GC Content
BadAM36-3ACph2	808419-816821	incomplete(10)	8.4Kb	60
BadAM34-11ph1	762892-771294	incomplete(10)	8.4Kb	60
BadAM34-11ph2	936194-944734	incomplete(30)	8.5Kb	60.4
BadAM14-37ph1	782533-791072	incomplete(30)	8.5Kb	60.5
BadAM12-59ph1	478711-488715	incomplete(30)	10Kb	58.5
BadAM12-20ph1	711057-721040	incomplete(30)	9.9Kb	58.4
BadAM13-11ph1	666991-675531	incomplete(30)	8.5Kb	60.4
BadAM13-11ph2	1252914-1261060	incomplete(20)	8.1Kb	62.5
BadAM13-11ph3	1534027-1542152	incomplete(10)	8.1Kb	58.3
BadAF45-19ph1	402635-444220	incomplete(50)	41.5Kb	58.9
BadP2P3ph1	849943-872844	incomplete(50)	22.9Kb	58.2
Badca-0067ph1	629702-638099	incomplete(10)	8.3Kb	59.9
Badca-0067ph2	814012-822551	incomplete(30)	8.5Kb	60.5
Bad1001271st1-A4ph1	4838-35003	incomplete(40)	30.1Kb	59.6
BadZJ2ph1	784350-794411	incomplete(30)	10Kb	58.4
BadZJ2ph2	2041051-2051182	incomplete(50)	10.1Kb	61.6
BadBIO5485ph1	187731-196133	incomplete(10)	8.4Kb	60
BadBIO5485ph2	356775-365315	incomplete(30)	8.5Kb	60.5
BadMGYG-HGUT-02395ph1	1755699-1775279	questionable(80)	19.5Kb	52.2
BadMGYG-HGUT-02395ph2	1843106-1861999	incomplete(20)	18.8Kb	62.5

Table S2. Predicted Prophages of *Bifidobacterium bifidum.*

Prophage Name	Location	Completeness Score	Size	GC Content
Bif324Bph1	230781-237721	incomplete(40)	6.9Kb	63.1
Bif324Bph2	1694474-1707921	incomplete(30)	13.4Kb	64
Bif85Bph1	496687-503636	incomplete(40)	6.9Kb	63.3
Bif85Bph2	537502-578477	questionable(80)	40.9Kb	62.5
Bif85Bph3	570910-588677	incomplete(20)	17.7Kb	64.5
BifAF11-25Bph1	112722-120287	incomplete(30)	7.5Kb	59
BifAM12-10ph1	295436-324042	incomplete(30)	28.6Kb	53.9
BifAM12-10ph2	328837-341545	incomplete(50)	12.7Kb	64.4
BifAM18-1ph1	182943-195688	incomplete(60)	12.7Kb	62.3
BifAM18-11ph1	182912-195657	incomplete(60)	12.7Kb	62.3
BifAM18-12ACph1	182910-195655	incomplete(60)	12.7Kb	62.3
BifAM18-29ph1	182906-195651	incomplete(60)	12.7Kb	62.3
BifAM36-1ACph1	824235-832894	incomplete(10)	8.6Kb	63.6
BifAM42-15ACph1	286141-294812	incomplete(10)	8.6Kb	63.5
BifATCC29521.3ph1	1061146-1079728	incomplete(30)	18.5Kb	53.9
BifATCC29521.3ph2	1086540-1101890	incomplete(30)	15.3Kb	63.9
BifBF3ph1	211628-238531	incomplete(40)	26.9Kb	60.9
BifBF3ph2	1067540-1076199	incomplete(10)	8.6Kb	63.6
BifBGN4ph1	1899838-1906788	incomplete(40)	6.9Kb	63.2
BifBGN4ph2	1953346-1966886	incomplete(50)	13.5Kb	62.1
BifBIO5480ph1	584510-591477	incomplete(10)	6.9Kb	64.2
BifBIOML-A4ph1	464008-515228	questionable(90)	51.2Kb	61.9
BifBIOML-A6ph1	469236-520457	questionable(90)	51.2Kb	61.9
BifBIOML-A8ph1	473096-524318	questionable(90)	51.2Kb	61.9

Prophage Name	Location	Completeness Score	Size	GC Content
BifBIOML-A9ph1	1572833-1580363	incomplete(30)	7.5Kb	66
Bifca_0067ph1	473096-524318	questionable(90)	51.2Kb	61.9
BifJCM1254ph1	53217-60823	incomplete(30)	7.6Kb	62.4
BifLMG11041ph1	1705752-1741388	incomplete(60)	35.6Kb	63.5
BifMGYG-HGUT-02396 ph1	453620-469305	questionable(70)	15.6Kb	61.3
BifMGYG-HGUT-02396 ph2	1098707-1110895	incomplete(20)	12.1Kb	65.4
BifMGYG-HGUT-02396 ph3	1392523-1400834	incomplete(30)	8.3Kb	55.1
BifNCIMB41171.2ph1	857924-894667	questionable(80)	36.7Kb	62.8
BifNCIMB41171.2ph2	1710914-1717855	incomplete(40)	6.9Kb	63.2
BifNCTC13001ph1	1571608-1586958	incomplete(40)	15.3Kb	63.9
BifNCTC13001ph2	1593770-1612338	incomplete(30)	18.5Kb	53.9
BifPRI1ph1	1200976-1230606	questionable(80)	29.6Kb	57.9
BifPRI1ph2	1283980-1305203	incomplete(30)	21.2Kb	62
BifPRL2010ph1	1067430-1078444	incomplete(30)	11Kb	58.5
BifPRL2010ph2	1081028-1096867	incomplete(60)	15.8Kb	64
BifPRL2010ph3	1907504-1914445	incomplete(40)	6.9Kb	63.1
BifS17ph1	90873-115995	incomplete(20)	25.1Kb	64.2
BifS17ph2	1353831-1360433	incomplete(40)	6.6Kb	60.5
BifS6ph1	453620-469305	questionable(70)	15.6Kb	61.3
BifS6ph2	1098707-1110895	incomplete(20)	12.1Kb	65.4
BifS6ph3	1392523-1400834	incomplete(30)	8.3Kb	55.1
BifTF05-39ph1	1034444-1043103	incomplete(10)	8.6Kb	63.5
BifTF06-13ph1	368343-381098	incomplete(60)	12.7Kb	62.3
BifTF06-14ACph1	183378-196133	incomplete(60)	12.7Kb	62.3
BifTF06-14ACph2	242432-249381	incomplete(40)	6.9Kb	63.1
BifTM02-15ph1	292425-299375	incomplete(40)	6.9Kb	63.2
BifTM02-17ph1	13226-20176	incomplete(40)	6.9Kb	63.2
BifTM04-12ph1	63384-87370	incomplete(50)	23.9Kb	59.7
BifTM05-15ph1	1034248-1042907	incomplete(10)	8.6Kb	63.5
BifTM07-4ACph1	1023583-1032242	incomplete(10)	8.6Kb	63.5
BifTMC3115ph1	1079022-1085971	incomplete(40)	6.9Kb	63.1

Table S3. Predicted Prophages of *Bifidobacterium breve*

Prophage Name	Location	Completeness Score	Size	GC Content
Bre017W439ph1	1079650-1088168	incomplete(10)	8.5Kb	58
Bre017W439ph2	1210608-1218915	incomplete(20)	8.3Kb	62.7
Bre017W439ph3	2270509-2279795	incomplete(20)	9.2Kb	58.9
Bre082W48ph1	1203320-1217851	incomplete(30)	14.5Kb	62
Bre12Lph1	1031973-1040497	incomplete(10)	8.5Kb	57.9
Bre12Lph2	2213650-2222991	incomplete(20)	9.3Kb	58.8
Bre139W423ph1	1304994-1334589	incomplete(30)	29.5Kb	61.5
Bre180W83ph1	1188797-1203326	incomplete(20)	14.5Kb	62.2
Bre215W447aph1	1694064-1735429	incomplete(40)	41.3Kb	58.6
Bre2Lph1	1052109-1061453	incomplete(20)	9.3Kb	58.8
Bre689bph1	372269-390529	incomplete(20)	18.2Kb	61.4
BreACS-071-V-Sch8bph	1091201-1099719	incomplete(10)	8.5Kb	58

Prophage Name	Location	Completeness Score	Size	GC Content
1				
BreB.breve_1_modph1	1091518-1100044	incomplete(10)	8.5Kb	58
BreB.breve_1_modph2	2191415-2200551	incomplete(10)	9.1Kb	50.6
BreBBRI4ph1	59906-69192	incomplete(20)	9.2Kb	58.9
BreBifido_07ph1	1434448-1446917	incomplete(20)	12.4Kb	64.5
BreBIO6018ph1	1703385-1719163	incomplete(10)	15.7Kb	61.8
BreBR03ph1	1191028-1199551	incomplete(10)	8.5Kb	58
BreBR-14ph1	2522168-2528239	incomplete(10)	6Kb	63.5
BreBR-15ph1	278054-284595	incomplete(30)	6.5Kb	63.2
BreBR-19ph1	127725-135673	incomplete(50)	7.9Kb	61.9
BreBR3ph1	718527-744029	intact(140)	25.5Kb	57.9
BreBR3ph2	944950-959466	incomplete(30)	14.5Kb	62.1
BreBR3ph3	962586-972711	incomplete(40)	10.1Kb	61.6
BreBR3ph4	1649870-1679797	questionable(70)	29.9Kb	55.7
BreBR3ph5	1821902-1838587	incomplete(20)	16.6Kb	64.3
BreBR-H29ph1	357955-370402	incomplete(30)	12.4Kb	61.3
BreCNCMI-4321ph1	341784-359995	questionable(80)	18.2Kb	61.3
BreCNCMI-4321ph2	372247-388945	incomplete(20)	16.6Kb	64.5
BreCNCMI-4321ph3	1547062-1588308	incomplete(40)	41.2Kb	58.6
BreDRBB26ph1	1249277-1263781	incomplete(20)	14.5Kb	62.1
BreDRBB27ph1	337879-377529	incomplete(40)	39.6Kb	63.4
BreDRBB27ph2	511686-543798	questionable(70)	32.1Kb	52.8
BreDRBB27ph3	543669-568735	questionable(90)	25Kb	58.9
BreDRBB27ph4	796327-813439	incomplete(10)	17.1Kb	62.7
BreDRBB28ph1	391088-409307	questionable(80)	18.2Kb	61.3
BreDRBB28ph2	408685-418777	incomplete(20)	10Kb	61.2
BreDRBB28ph3	417912-446347	incomplete(20)	28.4Kb	64.3
BreDRBB28ph4	506969-522553	incomplete(10)	15.5Kb	63.4
BreDRBB28ph5	1301924-1316427	incomplete(20)	14.5Kb	62.1
BreDRBB29ph1	337879-377529	incomplete(40)	39.6Kb	63.4
BreDRBB29ph2	511685-543798	questionable(70)	32.1Kb	52.8
BreDRBB29ph3	543669-568735	questionable(90)	25Kb	58.9
BreDRBB29ph4	796327-813439	incomplete(10)	17.1Kb	62.7
BreDRBB30ph1	341784-359995	questionable(80)	18.2Kb	61.3
BreDRBB30ph2	372247-388945	incomplete(20)	16.6Kb	64.5
BreDRBB30ph3	1547080-1588327	incomplete(40)	41.2Kb	58.6
BreDSM20213.3ph1	1468956-1494343	incomplete(20)	25.3Kb	61
BreDSM20213.3ph2	1497060-1508394	incomplete(10)	11.3Kb	57.3
BreFDAAR-GOS_561ph1	1476679-1502066	incomplete(20)	25.3Kb	61
BreFDAAR-GOS_561ph2	1504783-1516117	incomplete(10)	11.3Kb	57.3
BreHPH0326ph1	465004-481694	incomplete(20)	16.6Kb	64.4
BreIDCC4401ph1	1867555-1877818	incomplete(40)	10.2Kb	61.9
BreJCM7017ph1	1069727-1078244	incomplete(10)	8.5Kb	58
BreJCM7017ph2	2111284-2120420	incomplete(10)	9.1Kb	50.6
BreJCM7019ph1	1072175-1080699	incomplete(10)	8.5Kb	58
BreJCM7019ph2	1103111-1111689	incomplete(20)	8.5Kb	61
BreJCM7019ph3	2328044-2337390	incomplete(20)	9.3Kb	59
BreJR01ph1	1155840-1170398	incomplete(30)	14.5Kb	61.9

Prophage Name	Location	Completeness Score	Size	GC Content
BreJSRL01ph1	1096160-1104683	incomplete(10)	8.5Kb	58
BreJTLph1	1732953-1741476	incomplete(10)	8.5Kb	58
BreLMC520ph1	371504-388189	incomplete(20)	16.6Kb	64.3
BreLMC520ph2	534514-542513	incomplete(40)	8Kb	62.1
BreLMC520ph3	1211836-1224661	incomplete(20)	12.8Kb	62.5
Brelw01ph1	769955-801141	incomplete(20)	31.1Kb	58.6
BreMC1ph1	19466-28773	incomplete(20)	9.3Kb	59.1
BreMCC1454ph1	2148796-2157933	incomplete(10)	9.1Kb	50.8
BreMCC1605ph1	73817-88312	incomplete(30)	14.4Kb	62
BreMGYG-HGUT-02469ph1	430953-441216	incomplete(30)	10.2Kb	61.9
BreNCFB2258ph1	1061708-1070226	incomplete(10)	8.5Kb	58
BreNCTC11815ph1	1468957-1494345	incomplete(20)	25.3Kb	61
BreNCTC11815ph2	1497062-1508396	incomplete(10)	11.3Kb	57.3
BreNRBB01ph1	1468951-1494338	incomplete(20)	25.3Kb	61
BreNRBB01ph2	1497055-1508389	incomplete(10)	11.3Kb	57.4
BreNRBB02ph1	404211-413453	incomplete(50)	9.2Kb	61.4
BreNRBB02ph2	1110822-1119339	incomplete(10)	8.5Kb	58
BreNRBB02ph3	2106318-2129047	incomplete(30)	22.7Kb	51.4
BreNRBB04ph1	1061156-1069673	incomplete(10)	8.5Kb	58
BreNRBB04ph2	1732488-1740122	incomplete(10)	7.6Kb	59.8
BreNRBB08ph1	404207-413449	incomplete(50)	9.2Kb	61.4
BreNRBB08ph2	1110712-1119229	incomplete(10)	8.5Kb	58
BreNRBB08ph3	2116184-2125319	incomplete(10)	9.1Kb	50.6
BreNRBB09ph1	571542-579892	incomplete(30)	8.3Kb	59.3
BreNRBB09ph2	1030861-1039384	incomplete(10)	8.5Kb	57.9
BreNRBB09ph3	1151895-1159330	incomplete(50)	7.4Kb	62.6
BreNRBB09ph4	2080363-2090142	incomplete(10)	9.7Kb	54.7
BreNRBB11ph1	1079792-1088310	incomplete(10)	8.5Kb	58.1
BreNRBB18ph1	404211-413453	incomplete(50)	9.2Kb	61.4
BreNRBB18ph2	1110730-1119247	incomplete(10)	8.5Kb	58
BreNRBB18ph3	2116111-2125246	incomplete(10)	9.1Kb	50.6
BreNRBB19ph1	404201-413443	incomplete(50)	9.2Kb	61.4
BreNRBB19ph2	1110693-1119210	incomplete(10)	8.5Kb	58
BreNRBB19ph3	2116154-2125289	incomplete(10)	9.1Kb	50.6
BreNRBB20ph1	404214-413456	incomplete(50)	9.2Kb	61.4
BreNRBB20ph2	1110734-1119251	incomplete(10)	8.5Kb	58
BreNRBB20ph3	2116315-2125451	incomplete(10)	9.1Kb	50.6
BreNRBB27ph1	404218-413460	incomplete(50)	9.2Kb	61.4
BreNRBB27ph2	1110740-1119257	incomplete(10)	8.5Kb	58
BreNRBB27ph3	2116262-2125398	incomplete(10)	9.1Kb	50.6
BreNRBB49ph1	404214-413456	incomplete(50)	9.2Kb	61.4
BreNRBB49ph2	1110734-1119251	incomplete(10)	8.5Kb	58
BreNRBB49ph3	2116216-2125351	incomplete(10)	9.1Kb	50.6
BreNRBB50ph1	364649-388510	questionable(90)	23.8Kb	64.3
BreNRBB50ph2	383978-428095	incomplete(40)	44.1Kb	63.4
BreNRBB51ph1	1362341-1386380	incomplete(60)	24Kb	61.9
BreNRBB52ph1	348640-355612	incomplete(40)	6.9Kb	65.9
BreNRBB52ph2	356056-363417	incomplete(50)	7.3Kb	66.4
BreNRBB52ph3	1099378-1107895	incomplete(10)	8.5Kb	58.1

Prophage Name	Location	Completeness Score	Size	GC Content
BreNRBB52ph4	1199925-1209634	incomplete(30)	9.7Kb	63.3
BreNRBB52ph5	1214231-1224322	incomplete(20)	10Kb	62.5
BreNRBB52ph6	1691916-1702426	incomplete(60)	10.5Kb	65.4
BreNRBB56ph1	394382-398930	incomplete(30)	4.5Kb	65.7
BreNRBB56ph2	412485-424926	questionable(70)	12.4Kb	61.7
BreNRBB56ph3	1102537-1111560	incomplete(50)	9Kb	65.3
BreNRBB57ph1	871636-908528	incomplete(30)	36.8Kb	59.2
BreS27ph1	1108085-1116603	incomplete(10)	8.5Kb	58
BreSC95ph1	576306-600516	incomplete(30)	24.2Kb	61.4
BreUCC2003ph1	430953-441216	incomplete(30)	10.2Kb	61.9
BreUMB0089ph1	1326207-1338770	incomplete(10)	12.5Kb	63.4
BreUMB0089ph2	1865179-1882407	incomplete(40)	17.2Kb	62.2
BreUMB0915ph1	616474-622101	incomplete(60)	5.6Kb	59.6

Table S4a. Predicted Prophages of *Bifidobacterium longum* subsp. *longum*

Prophage Name	Location	Completeness Score	Size	GC Content
Blong105-Aph1	308589-317744	incomplete(60)	9.1Kb	63.5
Blong105-Aph2	494748-502345	incomplete(50)	7.5Kb	62.3
Blong105-Aph3	937659-948274	incomplete(10)	10.6Kb	61.4
Blong105-Aph4	971865-980258	incomplete(10)	8.3Kb	61
Blong105-Aph5	1343331-1350332	incomplete(50)	7Kb	64.5
Blong105-Aph6	2251480-2258541	incomplete(20)	7Kb	61.1
Blong1888Bph1	34298-44105	incomplete(20)	9.8Kb	60.1
Blong1890Bph1	9763-19685	incomplete(20)	9.9Kb	60.4
Blong35624ph1	466363-472248	incomplete(30)	5.8Kb	57.2
Blong44Bph1	2239485-2248010	incomplete(20)	8.5Kb	62.8
Blong51Aph1	319607-346471	incomplete(30)	26.8Kb	63.8
Blong72Bph1	26263-38866	incomplete(10)	12.6Kb	62.3
Blong9ph1	8245-18103	incomplete(20)	9.8Kb	60.3
BlongAF04-13ph1	202518-211594	incomplete(50)	9Kb	63.5
BlongAF05-16ph1	298792-318117	incomplete(20)	19.3Kb	64.1
BlongAF05-2ph1	298792-318117	incomplete(20)	19.3Kb	64.1
BlongAF08-2ph1	378664-396853	incomplete(20)	18.1Kb	61.1
BlongAF08-2ph2	530723-562241	incomplete(30)	31.5Kb	57
BlongAF08-2ph3	782738-818601	incomplete(20)	35.8Kb	61.7
BlongAF13-41ph1	309627-320242	incomplete(10)	10.6Kb	61.4
BlongAF13-41ph2	344600-352993	incomplete(10)	8.3Kb	61.1
BlongAF13-41ph3	398144-413270	incomplete(50)	15.1Kb	55.9
BlongAF14-22ph1	89894-100507	incomplete(10)	10.6Kb	61.5
BlongAF14-22ph2	124900-133293	incomplete(10)	8.3Kb	61
BlongAF14-34ph1	89882-100495	incomplete(10)	10.6Kb	61.5
BlongAF14-34ph2	124888-133281	incomplete(10)	8.3Kb	61
BlongAF26-10ph1	481338-491178	incomplete(20)	9.8Kb	60.4
BlongAF30-11ph1	275002-284895	incomplete(40)	9.8Kb	57.8
BlongAF30-12ph1	157624-167463	incomplete(20)	9.8Kb	60.4
BlongAF30-12ph2	512100-521566	incomplete(60)	9.4Kb	64.4
BlongAF34-9ACph1	787572-800839	incomplete(10)	13.2Kb	61.7
BlongAF35-13ACph1	107792-123761	incomplete(50)	15.9Kb	63.4

BlongAF35-13ACph2	330789-340426	incomplete(50)	9.6Kb	62.9
BlongAF36-1ph1	495602-505230	incomplete(50)	9.6Kb	62.2
BlongAH1206ph1	328378-346725	incomplete(20)	18.3Kb	64.2
BlongAH1206ph2	1085134-1115652	incomplete(40)	30.5Kb	54.8
BlongAM11-5ph1	298143-322979	questionable(80)	24.8Kb	59.2
BlongAM12-16ph1	468038-478653	incomplete(10)	10.6Kb	61.4
BlongAM12-16ph2	501441-509834	incomplete(10)	8.3Kb	61
BlongAM16-2ph1	495278-504731	incomplete(60)	9.4Kb	65.7
BlongAM20-3ph1	379114-389038	incomplete(20)	9.9Kb	60.3
BlongAM31-13LBph1	651785-684265	incomplete(30)	32.4Kb	56.1
BlongAM39-10ACph1	193133-201526	incomplete(10)	8.3Kb	61.1
BlongAM39-10ACph2	225883-236499	incomplete(10)	10.6Kb	61.4
BlongAM39-8ACph1	1362-6379	incomplete(40)	5Kb	62.1
BlongAPC1461ph1	884053-894640	incomplete(10)	10.5Kb	61.1
BlongAPC1462ph1	1122565-1129091	incomplete(10)	6.5Kb	63
BlongAPC1504ph1	2019092-2032921	incomplete(10)	13.8Kb	61.6
BlongATCC15697.2ph1	1652670-1694678	incomplete(40)	42Kb	56.2
BlongATCC15697.2ph2	1802506-1813161	incomplete(50)	10.6Kb	59.8
BlongATCC15697.2ph3	1974117-1991034	incomplete(10)	16.9Kb	62.8
BlongATCC55813ph1	49444-57845	incomplete(10)	8.4Kb	60.9
BlongATCC55813ph2	82238-92851	incomplete(10)	10.6Kb	61.4
BlongATCC55813ph3	1280947-1293384	incomplete(20)	12.4Kb	56.9
BlongBAMA-B05ph1	240296-267052	incomplete(30)	26.7Kb	58.9
BlongBAMA-B05ph2	262195-299307	incomplete(50)	37.1Kb	61.7
BlongBB-79ph1	152997-162202	incomplete(60)	9.2Kb	64.2
BlongBB-79ph2	2192673-2202775	incomplete(30)	10.1Kb	62.1
BlongBBMN68ph1	408237-416337	incomplete(30)	8.1Kb	59.4
BlongBBMN68ph2	681458-689851	incomplete(10)	8.3Kb	61.1
BlongBBMN68ph3	714208-724823	incomplete(10)	10.6Kb	61.4
BlongBBMN68ph4	808619-818338	incomplete(50)	9.7Kb	61.6
BlongBBMN68ph5	1668648-1678575	incomplete(20)	9.9Kb	60.5
BlongBBMN68ph6	1836540-1844435	incomplete(20)	7.8Kb	51.1
BlongBG7ph1	328431-340648	incomplete(20)	12.2Kb	63.2
BlongBifido_03ph1	1181963-1191063	incomplete(60)	9.1Kb	61.6
BlongBifido_04ph1	838634-850173	incomplete(10)	11.5Kb	63.8
BlongBifido_06ph1	605708-618220	incomplete(30)	12.5Kb	61.3
BlongBIO6283ph1	305549-342205	incomplete(50)	36.6Kb	61.6
BlongBIO6283ph2	332635-363146	incomplete(40)	30.5Kb	59.3
BlongBORIph1	503171-515880	questionable(70)	12.7Kb	62.6
BlongBORIph2	965672-976283	incomplete(10)	10.6Kb	61.3
BlongBORIph3	999865-1008258	incomplete(10)	8.3Kb	61
BlongBORIph4	1237987-1260491	incomplete(50)	22.5Kb	58.1
BlongBORIph5	1475058-1484184	incomplete(60)	9.1Kb	66.2
BlongBORIph6	2275093-2285017	incomplete(20)	9.9Kb	60.3
BlongBXY01ph1	1056702-1065095	incomplete(10)	8.3Kb	61
BlongBXY01ph2	1112645-1121086	incomplete(50)	8.4Kb	62
BlongBXY01ph3	1459459-1466557	incomplete(50)	7Kb	64.2
BlongBXY01ph4	2230557-2239424	incomplete(30)	8.8Kb	59.8
BlongBXY01ph5	2272399-2278381	incomplete(50)	5.9Kb	63.9
BlongBXY01ph6	2444230-2454156	incomplete(20)	9.9Kb	60.3
BlongC11A10Bph1	222663-232502	incomplete(20)	9.8Kb	60.5

BlongCACC517ph1	226172-236096	incomplete(20)	9.9Kb	60.3
BlongCACC517ph2	764791-773413	incomplete(50)	8.6Kb	66
BlongCACC517ph3	1235143-1245755	incomplete(10)	10.6Kb	61.4
BlongCACC517ph4	1270112-1278505	incomplete(10)	8.3Kb	61
BlongCCUG30698ph1	1136168-1141566	incomplete(30)	5.3Kb	65.6
BlongCCUG30698ph2	1220181-1244971	incomplete(40)	24.7Kb	65.4
BlongCCUG30698ph3	1343855-1370568	incomplete(30)	26.7Kb	61.9
BlongCCUG30698ph4	2420566-2434615	incomplete(30)	14Kb	59.6
BlongCECT7210.2ph1	306541-343865	questionable(80)	37.3Kb	60.6
BlongCECT7210.2ph2	618826-628580	questionable(70)	9.7Kb	62.2
BlongCECT7210.2ph3	749442-777421	incomplete(40)	27.9Kb	61.7
BlongCECT7210.2ph4	2265802-2277848	incomplete(50)	12Kb	61.9
BlongDJO10Aph1	1329811-1356785	incomplete(30)	26.9Kb	63.9
BlongDS15_3ph1	338300-347766	incomplete(60)	9.4Kb	64.3
BlongDS9_3ph1	878687-888153	incomplete(60)	9.4Kb	64.2
BlongDS9_3ph2	1610803-1617374	incomplete(10)	6.5Kb	62.9
BlongE18ph1	1043236-1053603	incomplete(30)	10.3Kb	55.6
BlongE18ph2	1053759-1068976	incomplete(30)	15.2Kb	56.8
BlongE18ph3	1231248-1245705	incomplete(30)	14.4Kb	62.3
BlongE18ph4	2030333-2051929	incomplete(20)	21.5Kb	61.4
BlongE18ph5	2335516-2349565	incomplete(30)	14Kb	59.5
BlongEK5ph1	899892-910324	incomplete(40)	10.4Kb	65.5
BlongF8ph1	1928281-1955722	incomplete(20)	27.4Kb	64.4
BlongGT15ph1	46542-56465	incomplete(20)	9.9Kb	60.3
BlongGT15ph2	191559-206718	incomplete(50)	15.1Kb	63.2
BlongGT15ph3	392094-401559	incomplete(60)	9.4Kb	64.3
BlongGT15ph4	1085793-1096413	incomplete(10)	10.6Kb	61.4
BlongGT15ph5	1120772-1129165	incomplete(10)	8.3Kb	61.1
BlongIndicaph1	8602-17003	incomplete(10)	8.4Kb	61
BlongIndicaph2	482903-506278	incomplete(50)	23.3Kb	60.2
BlongJCM1217ph1	1149809-1183774	incomplete(50)	33.9Kb	67
BlongJDM301ph1	1055245-1063638	incomplete(10)	8.3Kb	61
BlongJDM301ph2	1111188-1119629	incomplete(50)	8.4Kb	62
BlongJDM301ph3	1460029-1467127	incomplete(50)	7Kb	64.2
BlongJDM301ph4	2227785-2236649	incomplete(30)	8.8Kb	59.8
BlongJDM301ph5	2269629-2275611	incomplete(50)	5.9Kb	63.9
BlongJDM301ph6	2441465-2451391	incomplete(20)	9.9Kb	60.3
BlongJih1ph1	487560-493687	incomplete(60)	6.1Kb	59.7
BlongJSRL02ph1	320705-343093	incomplete(20)	22.3Kb	64
BlongJSRL02ph2	917718-938198	incomplete(30)	20.4Kb	57.4
BlongJSRL03ph1	110105-122241	incomplete(60)	12.1Kb	63.7
BlongKACC91563ph1	752881-769699	incomplete(50)	16.8Kb	59.5
BlongKACC91563ph2	956606-964440	questionable(70)	7.8Kb	63.4
BlongKACC91563ph3	1289161-1297553	incomplete(10)	8.3Kb	61
BlongKACC91563ph4	1321143-1331757	incomplete(10)	10.6Kb	61.3
BlongKACC91563ph5	2147222-2159581	incomplete(50)	12.3Kb	64
BlongKACC91563ph6	2317626-2325087	incomplete(20)	7.4Kb	61.4
BlongLMG21814ph1	1512573-1521037	incomplete(10)	8.4Kb	59.9
BlongLO-K29aph1	209961-223152	incomplete(10)	13.1Kb	62.6
BlongLO-K29bph1	42724-55996	incomplete(20)	13.2Kb	62.6
BlongLTBL16ph1	503621-510397	incomplete(40)	6.7Kb	60.7

BlongMC1ph1	1914818-1920286	incomplete(10)	5.4Kb	59.1
BlongMCC10008ph1	2269498-2280791	incomplete(20)	11.2Kb	63.3
BlongMCC10023ph1	298935-305995	incomplete(20)	7Kb	60.9
BlongMCC10029ph1	109496-119419	incomplete(20)	9.9Kb	60.5
BlongMCC10038ph1	269903-282005	incomplete(10)	12.1Kb	62.7
BlongMCC10040ph1	14503-24343	incomplete(20)	9.8Kb	60.5
BlongMCC10040ph2	2411281-2419998	incomplete(40)	8.7Kb	63.3
BlongMCC10044ph1	1125147-1133332	incomplete(10)	8.1Kb	62.3
BlongMCC10052ph1	2000074-2008170	incomplete(20)	8Kb	63.4
BlongMCC10053ph1	1731007-1739822	incomplete(20)	8.8Kb	61.7
BlongMCC10056ph1	158693-168533	incomplete(20)	9.8Kb	60.6
BlongMCC10062ph1	469-10309	incomplete(20)	9.8Kb	60.5
BlongMCC10064ph1	1131378-1139860	incomplete(10)	8.4Kb	62
BlongMCC10072ph1	127161-143129	incomplete(50)	15.9Kb	63.3
BlongMCC10073ph1	2231969-2238539	incomplete(20)	6.5Kb	62.8
BlongMCC10077ph1	1006544-1019749	incomplete(10)	13.2Kb	63
BlongMCC10078ph1	81662-91500	incomplete(20)	9.8Kb	60.4
BlongMCC10079ph1	1171822-1179248	incomplete(20)	7.4Kb	63.5
BlongMCC10081ph1	2252385-2259078	incomplete(20)	6.6Kb	60.8
BlongMCC10081ph2	2284422-2292746	incomplete(30)	8.3Kb	60.2
BlongMCC10090ph1	469-10308	incomplete(20)	9.8Kb	60.4
BlongMCC10091ph1	2342735-2349651	incomplete(20)	6.9Kb	61
BlongMCC10093ph1	1209742-1218806	incomplete(20)	9Kb	63.2
BlongMCC10094ph1	138321-145127	incomplete(40)	6.8Kb	64.5
BlongMCC10094ph2	148240-165556	incomplete(20)	17.3Kb	64.4
BlongMCC10094ph3	905104-943111	incomplete(30)	38Kb	61.8
BlongMCC10100ph1	1250296-1260777	incomplete(10)	10.4Kb	62.4
BlongMCC10101ph1	318634-326896	incomplete(30)	8.2Kb	64.9
BlongMCC10102ph1	2126332-2141751	incomplete(10)	15.4Kb	63.3
BlongMCC10103ph1	1282174-1295691	incomplete(40)	13.5Kb	61.8
BlongMCC10106ph1	2348261-2357752	incomplete(20)	9.4Kb	62.9
BlongMCC10107ph1	111155-121078	incomplete(20)	9.9Kb	60.4
BlongMCC10111ph1	1230058-1239586	incomplete(10)	9.5Kb	63.1
BlongMCC10113ph1	1018934-1029419	incomplete(10)	10.4Kb	62.7
BlongMCC10115ph1	2367468-2375493	incomplete(10)	8Kb	63.2
BlongMCC10116ph1	1109578-1118642	incomplete(10)	9Kb	62.9
BlongMCC10117ph1	29750-39587	incomplete(20)	9.8Kb	60.4
BlongMCC10118ph1	269-17143	incomplete(60)	16.8Kb	59
BlongMCC10120ph1	127704-137543	incomplete(20)	9.8Kb	60.3
BlongMCC10124ph1	2090530-2102622	incomplete(10)	12Kb	63.5
BlongMCC10127ph1	154669-164511	incomplete(20)	9.8Kb	60.3
BlongMCC10128ph1	142391-152313	incomplete(20)	9.9Kb	60.4
BlongMCC10128ph2	397273-404416	incomplete(20)	7.1Kb	63.8
BlongMCC10129ph1	101367-118688	incomplete(20)	17.3Kb	64.4
BlongMCC10130ph1	2315774-2328556	incomplete(20)	12.7Kb	60.3
BlongMGYG-HGUT-01292ph1	1652880-1694888	incomplete(40)	42Kb	56.2
BlongMGYG-HGUT-01292ph2	1802716-1813371	incomplete(40)	10.6Kb	59.7
BlongMGYG-HGUT-01292ph3	1974327-1991244	incomplete(10)	16.9Kb	62.8

BlongN3A01ph1	380875-392522	incomplete(30)	11.6Kb	61.8
BlongNCC2705ph1	1124467-1129936	incomplete(50)	5.4Kb	64.8
BlongNCIMB8809ph1	2000854-2023770	incomplete(20)	22.9Kb	61.4
BlongNCTC11818ph1	1149809-1183774	incomplete(50)	33.9Kb	67
BlongOF01-16ph1	81631-92247	incomplete(10)	10.6Kb	61.4
BlongOF01-16ph2	116452-124845	incomplete(10)	8.3Kb	61.1
BlongOM05-2BHph1	472070-481992	incomplete(20)	9.9Kb	60.4
BlongPC1ph1	1884438-1893792	incomplete(10)	9.3Kb	56.2
BlongPC4ph1	1641549-1651140	incomplete(20)	9.5Kb	63.6
Blongssp_longumph1	402978-411373	incomplete(10)	8.3Kb	60.6
BlongSu859ph1	1256623-1276895	incomplete(60)	20.2Kb	59.2
BlongSu859ph2	1680803-1704611	incomplete(20)	23.8Kb	59.4
BlongTF01-22ph1	402549-433185	incomplete(60)	30.6Kb	61.5
BlongTF01-22ph2	425360-438406	incomplete(10)	13Kb	64.2
BlongTF06-12ACph1	81274-114835	incomplete(20)	33.5Kb	56.1
BlongTF06-45Aph1	306454-317081	incomplete(10)	10.6Kb	61.3
BlongTF06-45Aph2	341438-349831	incomplete(10)	8.3Kb	61.1
BlongTF06-45Aph3	2298260-2306676	incomplete(10)	8.4Kb	62.2
BlongTF07-31ph1	46358-56709	incomplete(10)	10.3Kb	56.4
BlongTF07-39ph1	272871-283222	incomplete(10)	10.3Kb	56.4
BlongTF08-4ACph1	470587-480426	incomplete(20)	9.8Kb	60.4
BlongTM01-1ph1	115159-124363	incomplete(60)	9.2Kb	64.2
BlongTM01-1ph2	728693-738532	incomplete(20)	9.8Kb	60.3
BlongTM05-14ph1	542681-553323	incomplete(10)	10.6Kb	61.4
BlongTM05-14ph2	577528-585921	incomplete(10)	8.3Kb	61
BlongUMA3015ph1	1329558-1336622	incomplete(10)	7Kb	63.6
BlongUMB0788ph1	321488-330693	incomplete(60)	9.2Kb	64.2
BlongVKP-MAc-1636ph1	837922-844721	incomplete(30)	6.8Kb	61.9
BlongZJ1ph1	485204-494358	incomplete(40)	9.1Kb	58.5
BlongZJ1ph2	1017175-1034598	incomplete(40)	17.4Kb	62.7
BlongZJ1ph3	1132593-1161161	incomplete(30)	28.5Kb	63

Table S4b. Predicted Prophages of *Bifidobacterium longum* subsp. *infantis*

Prophage Name	Location	Completeness Score	Size	GC Content
BlongATCC15697ph1	1652880-1694888	incomplete(40)	42Kb	56.2
BlongATCC15697ph2	1802716-1813371	incomplete(40)	10.6Kb	59.7
BlongATCC15697ph3	1974327-1991244	incomplete(10)	16.9Kb	62.8
Blong157Fph1	1211424-1244738	incomplete(60)	33.3Kb	61.1
BlongEK3ph1	65933-75731	incomplete(20)	9.7Kb	59.9
Blong-BIC1206122787ph1	28071-37847	incomplete(20)	9.7Kb	60.7
Blong-BIC1206122787ph2	2016089-2025061	incomplete(10)	8.9Kb	62.3
Blong-BIC1307292462ph1	1727114-1741730	incomplete(20)	14.6Kb	55.7
Blong-BIC1401111250ph1	33888-43664	incomplete(20)	9.7Kb	60.7
Blong-BIC1401212621aph1	33812-43588	incomplete(20)	9.7Kb	60.7

Blong-BIC1401212621bph1	33904-43680	incomplete(20)	9.7Kb	60.7
Blong-BIB1401242951ph1	33876-43652	incomplete(20)	9.7Kb	60.7
Blong-BIB1401242951ph2	2023843-2031084	incomplete(20)	7.2Kb	62.4
Blong-BIB1401272845aph1	33880-43656	incomplete(20)	9.7Kb	60.7
Blong-BIB1401272845bph1	33904-43680	incomplete(20)	9.7Kb	60.7
Blong-BIB1401272845bph2	2022798-2031770	incomplete(10)	8.9Kb	62.3
BlongCECT7210ph1	306541-343865	questionable(80)	37.3Kb	60.6
BlongCECT7210ph2	618826-628580	questionable(70)	9.7Kb	62.2
BlongCECT7210ph3	749442-777421	incomplete(40)	27.9Kb	61.7
BlongCECT7210ph4	2265802-2277848	incomplete(50)	12Kb	61.9
BlongBT1ph1	697032-706839	incomplete(20)	9.8Kb	60.1
BlongBT1ph2	1348904-1357609	incomplete(10)	8.7Kb	60
BlongBT1ph3	2247424-2255817	incomplete(10)	8.3Kb	60.6
Blong1888Bph1	34298-44105	incomplete(20)	9.8Kb	60.1
BlongUBBI-01ph1	182153-191401	incomplete(10)	9.2Kb	59.3
BlongBi-26ph1	398457-406850	incomplete(10)	8.3Kb	60.5
BlongBIO5478ph1	88590-96983	incomplete(10)	8.3Kb	60.5
BlongNCTC11817ph1	1652674-1694682	incomplete(40)	42Kb	56.2
BlongNCTC11817ph2	1802510-1813165	incomplete(40)	10.6Kb	59.8
BlongNCTC11817ph3	1960812-1970727	incomplete(10)	9.9Kb	60.1
BlongNCTC11817ph4	1974023-1991039	incomplete(10)	17Kb	62.9
Blongssp__2_modph1	56581-67326	incomplete(20)	10.7Kb	60.7
Blongssp__2_modph2	217684-226878	incomplete(10)	9.1Kb	59.3
Blongssp__2_modph3	1641726-1650119	incomplete(10)	8.3Kb	60.6
Blongssp__3_modph1	1068838-1086178	incomplete(20)	17.3Kb	64.5
Blongssp__5ph1	246499-270059	questionable(70)	23.5Kb	59.7
Blongssp_infantis_6_USA001_1ph1	1098138-1115133	incomplete(10)	16.9Kb	62.8
Blongssp_infantis_6_USA001_1ph2	1276089-1286744	incomplete(40)	10.6Kb	59.7
Blongssp_infantis_6_USA001_1ph3	1401026-1428532	incomplete(30)	27.5Kb	55.7
BlongBG463.m5.93_JGph1	86387-94780	incomplete(10)	8.3Kb	60.6

Table S5. Results table of pVOG HMMER3 queries against the phage-protospacer hits.

Target	Query Name	E-value	Score	Bias
VOG5733	Ba-dAF45-19-spacer2_BreCNCMI-4321ph3_1_ORF1	0.035	13.2	0.0
VOG5532	Ba-dAM36-3AC-spacer25_BreNRBB56ph3_4_ORF1	7.6e-07	28.5	0.0
VOG4737	Ba-	0.0015	17.4	0.0

Target	Query Name	E-value	Score	Bias
	dAM36-3AC-spacer25_BreNRBB56ph3_4_ORF1			
VOG5461	Ba-dAM36-3AC-spacer25_BreNRBB56ph3_4_ORF1	0.056	11.3	0.0
VOG2674	Bi-fIPLA20017-spacer25_BifMGYG-HGUT-02396ph3_4_ORF1	0.017	14.2	0.1
VOG1026	Blong1897B-spacer70_BlongJCM1217ph1_1_ORF1	3.4e-09	35.7	0.6
VOG10509	Blong1897B-spacer70_BlongJCM1217ph1_1_ORF1	0.039	13.2	0.1
VOG4576	Blong35624-spacer136_BlongBifido_06ph1_4_ORF1	0.23	10.5	1.2
VOG1125	Blon-gATCC15697-spacer0_BlongATCC15697.2ph3_2_ORF1	0.0073	15.3	1.2
VOG1125	Blon-gATCC15697-spacer4_BlongATCC15697.2ph3_2_ORF1	0.0015	17.5	1.7
VOG7166	BlongJSRL02-spacer9_Blongssp__3_modph1_3_ORF1	0.04	12.8	0.0
VOG5782	BlongMCC10027-spacer10_BlongCCUG30698ph2_2_ORF1	0.023	13.2	0.0
VOG1815	BlongTF08-4AC-spacer48_Blong105-Aph2_4_ORF1	0.069	12.4	0.3
VOG7687	BreBR-10-spacer53_Bif85Bph2_5_ORF1	0.012	14.8	0.4
VOG5532	BreMC1-spacer6_BadBB23ph1_1_ORF1	0.044	13.2	0.0
VOG5765	BreMC1-spacer8_BreNRBB51ph1_4_ORF1	0.057	12.3	0.1
VOG5532	BreMC1-spacer9_BadBB23ph1_1_ORF1	8.7e-05	21.9	0.0
VOG9197	BreMC1-spacer9_BadBB23ph1_1_ORF1	0.022	13.5	0.0
VOG5532	BreMC1-spacer9_BifS17ph2_1_ORF1	5.3e-05	22.6	0.0
VOG9197	BreMC1-spacer9_BifS17ph2_1_ORF1	0.014	14.2	0.1

Table S6. Annotation of pVOG genes associated with phage-protospacer hits.

pVOG Target	Description
VOG01026	REFSEQ hypothetical protein
VOG01125	REFSEQ hypothetical protein
VOG01125	REFSEQ hypothetical protein
VOG01815	sp Q5PLI4 DGCQ_SALPA Probable diguanylate cyclase DgcQ
VOG02674	REFSEQ hypothetical protein
VOG04576	REFSEQ hypothetical protein
VOG04737	sp P0DSR1 A6_VAR67 Protein A6
VOG05461	REFSEQ hypothetical protein
VOG05532	sp Q9YX48 CAPSD_FDVS Putative major capsid protein
VOG05532	sp Q9YX48 CAPSD_FDVS Putative major capsid protein
VOG05532	sp Q9YX48 CAPSD_FDVS Putative major capsid protein
VOG05532	sp Q9YX48 CAPSD_FDVS Putative major capsid protein
VOG05733	REFSEQ hypothetical protein
VOG05765	REFSEQ hypothetical protein
VOG05782	REFSEQ hypothetical protein

VOG07166	REFSEQ hypothetical protein
VOG07687	REFSEQ hypothetical protein
VOG09197	REFSEQ hypothetical protein
VOG09197	REFSEQ hypothetical protein
VOG10509	REFSEQ hypothetical protein

Table S7. BLAST alignments of predicted spacer arrays against predicted prophages.

Spacer Name	Phage Name	Identity	E-value	Nucleotide Sequence
Bad22L-spacer23	BadAF21-27ph1	100.000	4.47e-11	TACGTCCATCGGCCACAATTGGACTGGGATGT
Bad22L-spacer69	BadTF06-29ph2	100.000	4.47e-11	GCGTGGGCTTTCTCCCTGACCAGCTCGAAGTC
Bad22L-spacer73	BadAM13-11ph3	100.000	4.47e-11	ACCACAGATGACTCATTCAATCTCGATCACAT
Bad6-spacer68	BadAF21-27ph1	100.000	1.66e-11	ATGAGCGCCTGTTGTTCGCTGGCTTTCATTGCT
Bad703B-spacer0	Bad487Bph3	100.000	3.77e-48	TGGTTCTGCGAAACTGACGATGGGTGCC-TATGAAGAGCAGTATGGGCATGTCGAT-TCCAAGCTGACAGCCACGGACAACAC-TGGTGGCCCAACCAGTACG
Bad703B-spacer1	Bad487Bph3	100.000	3.21e-13	GCCAAAGGCAACATCCGTTCCGTACCGAA-TAGTCAT
Bad703B-spacer2	Bad487Bph3	100.000	1.14e-19	GGTACGAACGTGAATGCGACGGACAA-TACTGCTGGTGCGACCGGTTCC
Bad70B-spacer74	Bad487Bph1	100.000	9.42e-14	GAACTGGGCCTGCGCGCGTCATACGCG-GATTGAACTA
BadAD2-8-spacer60	BreBIO6018ph1	100.000	5.02e-10	AAGACCCTGCTCGTGGAGGCGCAGACCGGC
BadAD2-8-spacer60	BlongLMG21814ph1	100.000	5.02e-10	AAGACCCTGCTCGTGGAGGCGCAGACCGGC
Ba-dAF14-56-spacer108	BreDRBB30ph3	100.000	1.09e-12	CGCGACCCATCAACAAAAAACATCATCGGG-GAGAG
Ba-dAF14-56-spacer108	BreCNCMI-4321ph3	100.000	1.09e-12	CGCGACCCATCAACAAAAAACATCATCGGG-GAGAG
Ba-dAF14-56-spacer108	Bad1-11ph1	100.000	1.09e-12	CGCGACCCATCAACAAAAAACATCATCGGG-GAGAG
Ba-dAF14-56-spacer21	Bad1-11ph1	100.000	3.69e-12	GTGAGGTGACCGCGAAATTCCAG-CAGGTCGCCAA
Ba-dAF14-56-spacer21	BreDRBB30ph3	100.000	4.77e-11	GAGGTGACCGCGAAATTCCAGCAGGTCGCCAA
Ba-dAF14-56-spacer21	BreCNCMI-4321ph3	100.000	4.77e-11	GAGGTGACCGCGAAATTCCAGCAGGTCGCCAA
Ba-dAF14-56-spacer32	BadAF21-27ph1	100.000	6.56e-10	GTCGTTGACGGTGGTCCAGTCGGTCGGGTC
Ba-dAF14-56-spacer54	BadBB23ph2	100.000	3.69e-12	GCATCTCGTCGATGCTCATCGGCTGCAC-GTCCGT
Ba-dAF14-56-spacer80	BadTF06-29ph2	100.000	3.69e-12	AAGAGCAGCGAATCACCGCCGTGAC-CATCCAAGC
Ba-dAF15-3-spacer112	BreDRBB30ph3	100.000	4.84e-12	CGAAAACCCATACAAGGAG-TAGGCAAATGAAGAA
Ba-dAF15-3-spacer112	BreCNCMI-4321ph3	100.000	4.84e-12	CGAAAACCCATACAAGGAG-TAGGCAAATGAAGAA
Ba-dAF15-3-spacer142	Bad487Bph3	100.000	9.91e-14	GCGCCAACCAGCACGGCCAGAGCGTTCAAC-GTGGTCA
BadAF15-3-spacer78	BreDRBB30ph3	100.000	3.69e-12	TGGCGATCATGTCCGCCAAA-GCGGGCGTCAAAGT
BadAF15-3-spacer78	BreCNCMI-4321ph3	100.000	3.69e-12	TGGCGATCATGTCCGCCAAA-GCGGGCGTCAAAGT
BadAF15-3-spacer78	Bad1-11ph1	100.000	3.69e-12	TGGCGATCATGTCCGCCAAA-

Spacer Name	Phage Name	Identity	E-value	Nucleotide Sequence
BadAF15-3-spacer86	Bad1-11ph1	100.000	1.09e-12	GCGGGCGTCAAAGT GCCATCCAAGTCGGCGAACACGAC-GCCAGACTCGT
BadAF28-4AC-spacer53	BlongLMG21814ph1	100.000	5.02e-10	GACAAGTGTCCGGTGGATGATGCCGACGCT
BadAF28-4AC-spacer53	BlongAF08-2ph1	100.000	1.80e-09	GACAAGTGTCCGGTGGATGATGCCGACGC
BadAF28-4AC-spacer53	Bad1-11ph1	100.000	1.80e-09	GACAAGTGTCCGGTGGATGATGCCGACGC
BadAF28-4AC-spacer61	Bad1-11ph1	100.000	5.02e-10	GCATCACATCCAAAGACAGTTCAGGCGAGG
BadAF28-4AC-spacer85	Bad1-11ph1	100.000	5.02e-10	CAATTGCGCAGCGAATTCGGCACGATGCTG
BadAF45-19-spacer0	BlongIndicaph2	98.000	8.17e-45	AGCCTGACGGCCTTCGCCTT-GAACTCCGGCGTGTATCTCGTTCCTTTT-GCCATGGTTCCATCATCTCCCATCGAG-TTAGGGGAAATGCGGAACAAAAATC
BadAF45-19-spacer31	BadATCC15703ph1	98.020	8.17e-45	ATTTTT-GTTCCGCCCTTATAGTGGGGC-GCTTGCGCCGCT TGGTTTGCATAA-TACTCGGTTTCGGTCCGTTCCGGTGTCCTG-TAGCCCAAGGACCGGTGCA
BadAF45-19-spacer41	BreDRBB30ph3	100.000	9.42e-14	AGTCTTCTGCATGACTAGCGTCCCGTTT-GCGCGGGAA
BadAF45-19-spacer41	BreCNCMI-4321ph3	100.000	9.42e-14	AGTCTTCTGCATGACTAGCGTCCCGTTT-GCGCGGGAA
BadAL46-2-spacer105	BadTF06-29ph2	100.000	2.25e-10	CGTGTATTCGTGTTTCAACGTGTTCCTTTCC
BadAL46-2-spacer135	BadTF06-29ph2	100.000	3.69e-12	CACAAGCCGATTTCTGGCCGGACGATAAC-GCCAG
BadAL46-2-spacer135	Bad1-11ph1	100.000	3.69e-12	CACAAGCCGATTTCTGGCCGGACGATAAC-GCCAG
BadAL46-2-spacer143	Bad22Lph2	100.000	9.42e-14	GCAAGGGCTGGAGCGGCAAGAC-CGTGGCCGACACCTG
BadAL46-2-spacer153	BadTF06-29ph2	100.000	9.42e-14	GCAAGCTCGACAACGTTTCGCTGCCGAC-GTTCCTCAA
BadAL46-2-spacer153	BadAM13-11ph3	100.000	9.42e-14	GCAAGCTCGACAACGTTTCGCTGCCGAC-GTTCCTCAA
BadAL46-2-spacer43	BifPRL2010ph1	100.000	9.91e-14	AGAGACATGGGAGCAGTATGAGCAAC-GACTGTTCCAT
BadAL46-2-spacer49	BadAM13-11ph2	100.000	3.21e-13	ACTTGCAC-CATGCCCGAAGGGTCTGGTGCTTTTCTT
BadAL46-2-spacer57	Bad487Bph1	100.000	1.09e-12	TAGCTGGCTCCGCCTT-GCTGGGTGAATCCGCCACC
BadAL46-2-spacer63	BadTF06-29ph2	100.000	1.09e-12	CGAC-GAAATCGCTCAGGCGAAGTCCGACATCGCGG
BadAL46-2-spacer67	BadBB23ph2	100.000	3.21e-13	ATCCGACGCGACCAGACCGGACGGCATCAC-CGTCGC
BadAL46-2-spacer99	Bad42Bph1	100.000	3.69e-12	CGCAGCTGAAGTACCTGCACTAC-CAGATTGCCTC
BadAL46-2-spacer99	Bad22Lph2	100.000	3.69e-12	CGCAGCTGAAGTACCTGCACTAC-

Spacer Name	Phage Name	Identity	E-value	Nucleotide Sequence
				CAGATTGCCTC
Ba-dAM12-20-spacer80	BadBB23ph2	100.000	6.17e-10	GAGGACGGCGACATCGACGACCCGGCCAAG
Ba-dAM12-20-spacer84	BadAF21-27ph1	100.000	2.76e-14	GTCGAC-CATGATCATAGCTTCAACAAACTTCTGAAAGA
Ba-dAM12-20-spacer88	Bad1-11ph1	100.000	5.67e-11	AACGGCGAGGAGGCGATCATCACCCGCTCGAA
Ba-dAM13-11-spacer154	BreBIO6018ph1	100.000	8.48e-09	TATCCTGCTCAATCGCTCTGAATGATAT
Ba-dAM13-11-spacer96	BadTF06-29ph2	100.000	1.09e-12	ATTGAGTGGCGTGCCGATCATCGGCAG-CATGTTTG
Ba-dAM14-37-spacer110	BifTF06-13ph1	100.000	1.72e-10	GTGACCTGGTAGGCCGCCAGCGTGCCGGCTC
Ba-dAM14-37-spacer110	BlongAF04-13ph1	100.000	2.22e-09	GACCTGGTAGGCCGCCAGCGTGCCGGCTC
Ba-dAM14-37-spacer110	Blong105-Aph1	100.000	2.22e-09	GACCTGGTAGGCCGCCAGCGTGCCGGCTC
Ba-dAM14-37-spacer114	BlongAF04-13ph1	100.000	1.24e-11	CTCTCGAATCGCAGAAGGACCGCGACGG-CATCA
Ba-dAM14-37-spacer114	Blong105-Aph1	100.000	1.61e-10	CTCGAATCGCAGAAGGACCGCGACGGCATCA
Ba-dAM14-37-spacer142	Bad22Lph2	100.000	3.69e-12	GAAGAG-CATGTCGGCCTGCTCGATGGCCTCCTGC
Ba-dAM14-37-spacer19	BadAF21-27ph1	100.000	3.28e-20	GCAGGGAGTGCAAGGACCGCAAGGCCCGAC-CGGGGCCACCGGAGCGACC
Ba-dAM41-17-spacer18	BlongAF04-13ph1	100.000	1.67e-12	ATCGCCATGCAATCATCCGACTAC-CTCGCGGTCGG
Ba-dAM41-17-spacer18	Blong105-Aph1	100.000	1.67e-12	ATCGCCATGCAATCATCCGACTAC-CTCGCGGTCGG
BadA-TCC15703-spacer106	Bad1-11ph1	100.000	3.69e-12	TGGATGTCTGCACGACGTACCTGCACCGG-TATAA
BadA-TCC15703-spacer140	Bad487Bph3	100.000	1.72e-10	GACGCTCCTGCGGGTAACGCTCGTAATCAGG
BadA-TCC15703-spacer142	BadAF45-19ph1	100.000	1.33e-11	GCCACGTCCGACCATCACGCCGAC-CTCGTCGCG
BadA-TCC15703-spacer142	Bad1001271st1_A4ph1	100.000	1.33e-11	GCCACGTCCGACCATCACGCCGAC-CTCGTCGCG
BadA-TCC15703-spacer146	Bad1-11ph1	100.000	1.33e-11	AACTACAGCCTCCCGCTCAACCTGAGTGAC-CTG
BadA-TCC15703-spacer154	BreNRBB52ph4	100.000	1.33e-11	ACGGACGGCTGGTATAC-GGCGGCCGAGGTACGG
BadA-TCC15703-spacer164	BadAF21-27ph1	100.000	1.09e-12	CCCACGGCTCAACACATGGCTCCGCGAC-CAATGGG
BadA-TCC15703-spacer38	BifMGYG-HGUT-02396ph1	100.000	6.56e-10	GGCGTGATGGACGTGGGCGACTCGACCGTG
BadA-TCC15703-spacer38	BifAM18-11ph1	100.000	6.56e-10	GGCGTGATGGACGTGGGCGACTCGACCGTG
BadA-TCC15703-spacer56	BifTF06-13ph1	100.000	1.24e-11	CGGGTTCAGCTGCGCCTTGGTCAGGTCGGCGCT
BadA-TCC15703-spacer56	BifMGYG-HGUT-02396ph1	100.000	1.24e-11	CGGGTTCAGCTGCGCCTTGGTCAGGTCGGCGCT
BadA-TCC15703-spacer56	BifAM18-11ph1	100.000	1.24e-11	CGGGTTCAGCTGCGCCTTGGTCAGGTCGGCGCT
BadA-TCC15703-spacer60	Bad1-11ph1	100.000	1.09e-12	GCG-TATTGCGGGCGAATCATGTCGCCATGCTCGGC
BadA-	BadTF06-29ph2	100.000	1.33e-11	TTCGCATGTGCTGGGATTCTTCCTCTTCATCCA

Spacer Name	Phage Name	Identity	E-value	Nucleotide Sequence
TCC15703-spacer78				
BadA-TCC15703-spacer80	Bad1-11ph1	100.000	1.33e-11	TCGCTTGATGCTTATGGCGTGGCTCAGGCTTGA
BadA-TCC15703-spacer94	BadAF21-27ph1	100.000	3.69e-12	TTTGGAGCACGGCTGGATCGGAGA-GAACCCCATT
Bad-BIO5485-spacer104	Bad1-11ph1	100.000	4.47e-11	TACGACACTCTGGAAATCTCTCAGACACTCTT
Badca-spacer19	BifAM12-10ph1	100.000	3.69e-12	TCTCCCAGCTGACGCCGTCCGCCTTGGTG-TAGGT
Badca-spacer19	Bad487Bph1	100.000	3.69e-12	TCTCCCAGCTGACGCCGTCCGCCTTGGTG-TAGGT
Badca-spacer19	Bad1001271st1_A4 ph1	100.000	3.69e-12	TCTCCCAGCTGACGCCGTCCGCCTTGGTG-TAGGT
Badca-spacer43	Bad22Lph2	100.000	1.09e-12	ACGTTCTTCATCATGTTGTAGGCGGCGGAATT-GGT
Badca-spacer49	BadTF06-29ph2	100.000	4.77e-11	GATAGGAGGAACACGATGAGCCTCACCAC-GGA
Badca-spacer49	Bad1-11ph1	100.000	4.77e-11	GATAGGAGGAACACGATGAGCCTCACCAC-GGA
Badca-spacer69	BadAM13-11ph2	100.000	1.24e-11	CAAATGGCGCCGTG-GACAGATCGTCGGCGGCCA
Badca-spacer75	BadP2P3ph1	100.000	1.24e-11	TTTCCACTGCCTGCTTGCGAGTGCGGCGTGGCT
Badca-spacer75	Bad1-11ph1	100.000	1.24e-11	TTTCCACTGCCTGCTTGCGAGTGCGGCGTGGCT
Badca-spacer79	BadTF06-29ph2	100.000	3.69e-12	CGGCGTGGCCGCGATGCTGAAATTCGCCAC-CGTG
Badca-spacer79	Bad1-11ph1	100.000	4.77e-11	CGGCGTGGCCGCGATGCTGAAATTCGCCACCG
Badca-spacer97	BadTF06-29ph2	100.000	1.24e-11	CAGCTCGAAGTCGTTCAGCCCGATTTCTTTGGA
BadIVS-1-spacer5	BadBB23ph1	98.000	8.17e-45	CTGCCGCTTGAACGACTCCTCGTAG-TGGCGGG-GATGCTTCGGGTCGGCCATCGTCGGCCTCCAATCTCATGTCATTCGTCCCTCATCG-GACTGTCTAATA
BadIVS-1-spacer5	BadBB23ph1	98.000	8.17e-45	CTGCCGCTTGAACGACTCCTCGTAG-TGGCGGG-GATGCTTCGGGTCGGCCATCGTCGGCCTCCAATCTCATGTCATTCGTCCCTCATCG-GACTGTCTAATA
BadIVS-1-spacer5	BreNRBB51ph1	98.039	3.04e-19	CTGCCGCTTGAACGACTCCTCGTAG-TGGCGGGGATGCTTCGGGTCGGCCAT
BadIVS-1-spacer7	Bad1-11ph1	100.000	3.69e-12	TGAGGGGCACTGTCTCTTATCACGGG-CATTCAAA
BadKm4-spacer107	Bad487Bph3	100.000	4.47e-11	GTGACCCTCAAGTGAGGTTAAACCGTTGGAAT
BadL2-32-spacer122	BreNRBB01ph2	100.000	3.69e-12	GCGCCAGGTCGATCATGCCGCGA-TAGCCCATGAT
BadL2-32-spacer122	BreDSM20213.3ph2	100.000	3.69e-12	GCGCCAGGTCGATCATGCCGCGA-TAGCCCATGAT
BadL2-32-spacer126	BifNCTC13001ph2	100.000	1.09e-12	CGTCCGGTCGCCGGGTCGGTTCCCATGTCCTT-GCG
BadL2-32-spacer126	Bi-fATCC29521.3ph1	100.000	1.09e-12	CGTCCGGTCGCCGGGTCGGTTCCCATGTCCTT-GCG
BadL2-32-spacer126	BifAM12-10ph1	100.000	1.09e-12	CGTCCGGTCGCCGGGTCGGTTCCCATGTCCTT-GCG
BadL2-32-spacer132	Bad487Bph3	100.000	3.69e-12	TCAAATGGATGGTGCGATTGTTCGACTTGTCG-TA
BadL2-32-spacer140	BadAM13-11ph3	100.000	1.09e-12	TTACGCTCTGCCACTGAGCTACCGAAGCTG-GATAT

Spacer Name	Phage Name	Identity	E-value	Nucleotide Sequence
BadL2-32-spacer166	Bad487Bph3	100.000	3.69e-12	AATTATGCGTGGTCGCGTCGCCGCCGTCGAACGA
BadL2-32-spacer168	BadTF06-29ph2	100.000	1.24e-11	CGATTATCTGACGTTCTCCGGTTTGTCGATGA
BadL2-32-spacer168	BadAM13-11ph3	100.000	1.24e-11	CGATTATCTGACGTTCTCCGGTTTGTCGATGA
BadL2-32-spacer182	BadP2P3ph1	100.000	1.24e-11	TGTACCGTTTATTCATTTCGCGTCCTCCTTCAT
BadL2-32-spacer182	Bad1-11ph1	100.000	1.24e-11	TGTACCGTTTATTCATTTCGCGTCCTCCTTCAT
BadL2-32-spacer75	BifTF06-13ph1	100.000	3.69e-12	GCCTGAACATCCAGAAGGCCACGCCCG-CAACATG
BadL2-32-spacer75	BifMGYG-HGUT-02396ph1	100.000	3.69e-12	GCCTGAACATCCAGAAGGCCACGCCCG-CAACATG
BadL2-32-spacer75	BifAM18-11ph1	100.000	3.69e-12	GCCTGAACATCCAGAAGGCCACGCCCG-CAACATG
BadL2-32-spacer77	BadAF21-27ph1	100.000	1.09e-12	CGCCTCTGGAACCACCACGCCAGCCAC-CATCAAAG
BadLMG11579-spacer126	BadAF21-27ph1	100.000	1.09e-12	CAACTTGCTAGAGGACAGCCAA-GCAAAAACATGGC
BadLMG11579-spacer134	BreDRBB30ph3	100.000	3.21e-13	GAATTTGCGGTATGTGGTGGCTTTGACGGTG-TATTC
BadLMG11579-spacer134	BreCNCMI-4321ph3	100.000	3.21e-13	GAATTTGCGGTATGTGGTGGCTTTGACGGTG-TATTC
BadLMG11579-spacer76	BadAF21-27ph1	100.000	1.09e-12	TTGCGCCGGGCCTCGCGCTGTTCCTCGGTGAGCGT
BadLMG11579-spacer86	BadTF06-29ph2	100.000	6.56e-10	GAGCGATTCGAGCGCCCCCGGCCAGATTTT
BadLMG11579-spacer98	Bad42Bph1	100.000	3.21e-13	CGCGGGCATGGAC-GCGAAGTCCAATGCGTTCAAGGT
BadLMG11579-spacer98	Bad22Lph2	100.000	3.21e-13	CGCGGGCATGGAC-GCGAAGTCCAATGCGTTCAAGGT
BadLMG18897-spacer19	BadTF06-29ph2	100.000	6.81e-16	GTGGGATGGTACGCGCTTCGAGCCTTTTGAC-CTCGTGGCGA
BadLMG18897-spacer23	BadZJ2ph2	100.000	1.43e-12	TCAGTTCGCTTCCTGCAGGTT-GTCATCGAGCCGGGA
BadTM06-4-spacer100	BadAF21-27ph1	100.000	4.47e-11	GTGCTGGTCGCGCTCGACCAGCAGGACCGTCC
BadTM06-4-spacer102	BadAF21-27ph1	100.000	4.47e-11	TTTGACGCCGGCGACCTCGAGGTGCACGTCGG
BadTM06-4-spacer114	BreNRBB01ph2	100.000	2.08e-09	GCATTCCAAGCCAACGACAAGGTCGTCTA
BadTM06-4-spacer114	BreDSM20213.3ph2	100.000	2.08e-09	GCATTCCAAGCCAACGACAAGGTCGTCTA
BadTM06-4-spacer144	BadAM13-11ph2	100.000	4.47e-11	GAAAAGATTCTCCTCTACGGAGATGCCATTCC
BadTM06-4-spacer144	Bad42Bph1	100.000	4.47e-11	GAAAAGATTCTCCTCTACGGAGATGCCATTCC
BadTM06-4-spacer144	Bad22Lph2	100.000	4.47e-11	GAAAAGATTCTCCTCTACGGAGATGCCATTCC
BadTM06-4-spacer70	BreDRBB30ph3	100.000	4.47e-11	AAGTCCATGGCCTTCTGCGTCGGCACGTTGCG
BadTM06-4-spacer70	BreCNCMI-4321ph3	100.000	4.47e-11	AAGTCCATGGCCTTCTGCGTCGGCACGTTGCG
BadTM06-51-spacer28	BadTF06-29ph2	100.000	6.49e-09	GAGTGAAATTGTTGGCGGGTCGCGCGCG
BadTM06-51-spacer28	BadAM13-11ph3	100.000	6.49e-09	GAGTGAAATTGTTGGCGGGTCGCGCGCG
BadTM06-51-spacer78	BifTF06-13ph1	100.000	5.02e-10	TCGCAGCTCGACCTGCTGCACCGGCTCACC
BadTM06-51-spacer7	BifMGYG-HGUT-0	100.000	5.02e-10	TCGCAGCTCGACCTGCTGCACCGGCTCACC

Spacer Name	Phage Name	Identity	E-value	Nucleotide Sequence
8	2396ph1			
BadTM06-51-spacer78	BifAM18-11ph1	100.000	5.02e-10	TCGCAGCTCGACCTGCTGCACCGGCTCACC
BadTM06-51-spacer82	BadTF06-29ph2	100.000	5.02e-10	TACTGCGGATACCGCATGGTGTCCGCTCAG
BadTM06-51-spacer84	BlongSu859ph1	100.000	5.02e-10	ATAAAAGTCAATCGCATTGTCCAACTGCCA
BadTM06-51-spacer84	BadP2P3ph1	100.000	5.02e-10	ATAAAAGTCAATCGCATTGTCCAACTGCCA
BadZJ2-spacer76	BlongTF06-45Aph3	100.000	5.02e-10	TAAGTAAGGAGACGTGATATGGCTCGACAG
BadZJ2-spacer76	BlongAF08-2ph1	100.000	5.02e-10	TAAGTAAGGAGACGTGATATGGCTCGACAG
BadZJ2-spacer76	BadAM13-11ph3	100.000	5.02e-10	TAAGTAAGGAGACGTGATATGGCTCGACAG
Bif1887B-spacer106	BifPRI1ph2	100.000	4.47e-11	CGGCTGAGCTACGCGCATAGGTGGGTATGAGT
Bif1887B-spacer106	Brelw01ph1	100.000	5.79e-10	GCTGAGCTACGCGCATAGGTGGGTATGAGT
Bif1887B-spacer40	Bad487Bph1	100.000	4.47e-11	AGTAGATTTTGTAAAGAACCTATATAGGTAGA
Bif1887B-spacer8	BifAM12-10ph1	100.000	3.69e-12	GCGGCAATGTCAGTCATCTGCGTTTT-GCTAATCA
BifAF11-25B-spacer0	BifBF3ph1	100.000	3.09e-09	TTTGCGTTCTTAAGTGCGCGCCAAAACGAC
Bi-fATCC29521-spacer116	BifPRI1ph2	100.000	5.79e-10	GGATGCAAGCGTGTTGACCAGATTGCTCAG
Bi-fATCC29521-spacer80	BifPRL2010ph1	100.000	2.47e-10	GCAAGGGGTCGTTTAACACCGAGCAACTCGA
BifBIOML-A11-spacer12	BifPRL2010ph1	100.000	5.02e-10	CGAGCATCGTCGCCGTCTTCTCGAGTTGCT
BifBIOML-A11-spacer21	BifPRL2010ph1	100.000	5.02e-10	GAGCTAATCGGAGAGGATTCTCAGGAGATG
BifBIOML-A11-spacer47	Bif85Bph2	100.000	5.02e-10	TTTGACGACGCCGAGCGTGGTCGCGGTCGC
BifDS32-spacer10	BifTM04-12ph1	99.000	1.76e-46	ATGTGAGGCGGGACGTGACGAGCAGCACTG-CAAGTGCCGTCAGCG-CAGGGCCGCGGCCGAGGCAGCCG-CAGCGAACGCTTCGGCCGCTCAGCAGCCGGCC
BifDS32-spacer12	BifTM04-12ph1	100.000	1.67e-09	ACGGCGCCGCGGAAGCCGGCGAAGGCGAA
BifDS32-spacer16	BifTM04-12ph1	98.000	8.17e-45	ATGCCGAAGGCCACGCCATTTCCACGGAAC-CGCACCTGGGCTATGTGGCCGATCTT-GCATCGCTGATCGAGATCCAGCAG-GAATCCACCGTCTCGCGCGT
BifDS32-spacer59	Bif85Bph2	100.000	1.09e-12	GCACCGCAACCGGAACCACAGACACAG-GAGGCTCA
Bi-fIPLA20015-spacer41	Bif85Bph2	100.000	1.80e-09	AAGCCGGGGTCTATCACCCGCACGTCATA
Bi-fIPLA20017-spacer25	BifMGYG-HGUT-02396ph3	100.000	3.77e-48	ATTTGACTCGTT-GGGGTCCGGGGTTATTGCGTTT-GGGGGCCATTATGAGTTTT-GGTGGTTATTGCGTTTCGAGGCCGGGGTTATTGCGTTTGGGAGCCGT
Bi-fIPLA20017-spacer26	BifMGYG-HGUT-02396ph3	98.438	6.49e-26	GCACCATAACCCCGGCCTCCAAACACAA-TAACCAGTGGCCTCAAAAGCTACGAA-TATTCACATC
Bi-fIPLA20017-spacer54	BlongAM16-2ph1	100.000	5.02e-10	AACGAATCAAGCACGCCGTCGCGGATCAGC
Bi-fIPLA20017-spacer80	Bif85Bph2	100.000	5.02e-10	GGAAGTCACGGCGGCTACGGTTCACGTCCG
Bi-	Bif85Bph2	100.000	5.02e-10	GAAAGCGTTGCCGTCCAGCACCATGCTTGC

Spacer Name	Phage Name	Identity	E-value	Nucleotide Sequence
fIPLA20017-spacer82 Bi-fIPLA20017-spacer84	Bif85Bph2	100.000	5.02e-10	CCCTCAATCCGATTTCATGCTTCATTCTGA
BifMGYG-HGUT-02396-spacer122	BreNRBB50ph1	100.000	5.02e-10	CGAGAAGGCCGACGACGCCTTGCCGCGCAT
BifMGYG-HGUT-02396-spacer72	Bif85Bph2	100.000	5.02e-10	CACGCCCACGCCAAGCCAGCCGTCAACGGC
BifNCTC10471-spacer22	BlongAF08-2ph1	100.000	3.69e-12	CAACAGTCCGAGGTCACGGGCTCGGATAA-GCTGG
BifNCTC10471-spacer4	BadAF21-27ph1	100.000	1.24e-11	CGAAGAAGCACATGAGGATGGTGAAC-GCGGTCA
BifNCTC10471-spacer56	BreBIO6018ph1	100.000	3.69e-12	AGGGACTTGAC-GTAGAAGTCGCCGAGGGTCTGCG
BifNCTC10471-spacer56	Bad1-11ph1	100.000	3.69e-12	AGGGACTTGAC-GTAGAAGTCGCCGAGGGTCTGCG
BifNCTC10471-spacer6	BreDRBB27ph4	100.000	1.24e-11	CGGCAACAAGTCGGCGTG-GAGCGCGGGTGCCAG
BifNCTC10471-spacer6	BlongAF08-2ph1	100.000	1.24e-11	CGGCAACAAGTCGGCGTG-GAGCGCGGGTGCCAG
BifNCTC10471-spacer74	BreDRBB28ph1	100.000	1.09e-12	GTGTCCACCATGCGCAAGGCGGGCGCG-GACATGAA
BifNCTC10471-spacer74	BreCNCMI-4321ph1	100.000	1.09e-12	GTGTCCACCATGCGCAAGGCGGGCGCG-GACATGAA
BifNCTC10471-spacer74	Bre689bph1	100.000	1.09e-12	GTGTCCACCATGCGCAAGGCGGGCGCG-GACATGAA
BifNCTC10471-spacer74	Blon-gAF35-13ACph2	100.000	1.09e-12	GTGTCCACCATGCGCAAGGCGGGCGCG-GACATGAA
BifNCTC10471-spacer78	BreDRBB28ph1	100.000	3.21e-13	TCCACTTGGATTGCATCAGCCGTGACAC-CGACCCGG
BifNCTC10471-spacer78	BreCNCMI-4321ph1	100.000	3.21e-13	TCCACTTGGATTGCATCAGCCGTGACAC-CGACCCGG
BifNCTC10471-spacer78	Bre689bph1	100.000	3.21e-13	TCCACTTGGATTGCATCAGCCGTGACAC-CGACCCGG
BifNCTC10471-spacer8	BifPRI1ph2	100.000	3.21e-13	ACGATCCGCTCGATCGCGACGCGGAACGG-GACGCAC
BifNCTC10471-spacer80	BreDRBB28ph1	100.000	1.24e-11	ACCGTGCCCGACACGGCCGGCACGCTGGAA-TAC
BifNCTC10471-spacer80	BreCNCMI-4321ph1	100.000	1.24e-11	ACCGTGCCCGACACGGCCGGCACGCTGGAA-TAC
BifNCTC10471-spacer80	Bre689bph1	100.000	1.24e-11	ACCGTGCCCGACACGGCCGGCACGCTGGAA-TAC
BifNCTC10471-spacer82	Bif85Bph2	100.000	3.69e-12	AGCGTGGAGTGGGAGCGGGCCGGGACGGAG-CAGG
BifNCTC10471-spacer84	BreDRBB28ph1	100.000	3.21e-13	CTCCGGGGATTGAAAAGCGCATGGGAC-GGCGTGACC
BifNCTC10471-spacer84	BreCNCMI-4321ph1	100.000	3.21e-13	CTCCGGGGATTGAAAAGCGCATGGGAC-GGCGTGACC
BifNCTC10471-spacer84	Bre689bph1	100.000	3.21e-13	CTCCGGGGATTGAAAAGCGCATGGGAC-GGCGTGACC
BifNCTC10471-spacer84	Blon-gAF35-13ACph2	100.000	3.21e-13	CTCCGGGGATTGAAAAGCGCATGGGAC-GGCGTGACC
BifNCTC10471-spacer87	BreBR3ph3	100.000	1.77e-41	GTAAAACAAACCACCGGCCGGCAC-TCCTCATGCCGAAAACCGAAAAAAATTAG-TGACGAAACACGAAACACCGACTT-GACTAAAAACA
BifNCTC10471-space	BreBR-19ph1	100.000	1.77e-41	GTAAAACAAACCACCGGCCGGCAC-

Spacer Name	Phage Name	Identity	E-value	Nucleotide Sequence
r87				TCCTCATGCCGAAAACCGAAAAAAATTAG-TGACGAAACACGAAACACCGACTT-GACTAAAAACA
BifNCTC10471-spacer87	BlongJCM1217ph1	98.864	8.22e-40	GTAAAACAAACCACCGGCCGGCAC-TCCTCATGCCGAAAACCGAAAAAAATTAG-TGACGAAACACGAAACACCGACTT-GACTAAAAACA
BifS17-spacer58	BlongAM16-2ph1	100.000	1.50e-10	GCCGAAGATTGCTCGGAACGGTCGGGCCGTA
BifTF07-22-spacer148	BifTF06-13ph1	100.000	3.69e-12	ACCGCATTGCAGACGGCCGCGCAG-TCGGTCGGCG
BifTF07-22-spacer148	BifMGYG-HGUT-02396ph1	100.000	3.69e-12	ACCGCATTGCAGACGGCCGCGCAG-TCGGTCGGCG
BifTF07-22-spacer148	BifAM18-11ph1	100.000	3.69e-12	ACCGCATTGCAGACGGCCGCGCAG-TCGGTCGGCG
Blong105-A-spacer47	BlongE18ph4	100.000	1.67e-09	TTGACTCGTTCTCGTGCGGCGGCGAGGCG
Blong105-A-spacer47	BlongBB-79ph2	100.000	1.67e-09	TTGACTCGTTCTCGTGCGGCGGCGAGGCG
Blong105-A-spacer47	BlongAPC1504ph1	100.000	1.67e-09	TTGACTCGTTCTCGTGCGGCGGCGAGGCG
Blong105-A-spacer47	Blon-gAF34-9ACph1	100.000	1.67e-09	TTGACTCGTTCTCGTGCGGCGGCGAGGCG
Blong105-A-spacer71	Blong157Fph1	100.000	5.49e-09	ATCATCGTGAAAGGCACACCAAATGACT
Blong105-A-spacer85	Blong-BAMA-B05ph1	100.000	5.49e-09	TCGGGTCGTATGTGACGTCGCCATGCAC
Blong12-spacer0	BlongBORIph1	100.000	2.30e-35	TTCCGTGGTCGG-CATGCTCTGCCTAAACCCGAAAAATCATCGTG ACTAAACCCGCAACAACTAATT-GAACACAAACA
Blong12-spacer0	Blong105-Aph2	100.000	6.54e-21	TTCCGTGGTCGG-CATGCTCTGCCTAAACCCGAAAAATCATCGTG ACTAAAC
Blong12-spacer0	Blong105-Aph2	100.000	1.11e-08	AACCCGCAACAACTAATTGAACACAAACA
Blong12-spacer54	BlongMCC10130ph1	100.000	5.49e-09	ACGGCGAGCCTCATATCCGCAGCCGTCA
Blong12-spacer56	BlongJCM1217ph1	100.000	5.49e-09	GATCGGCGCGGCGGGCGACAGCCAGTGG
Blong12-spacer56	Blong-CCUG30698ph2	100.000	5.49e-09	GATCGGCGCGGCGGGCGACAGCCAGTGG
Blong12-spacer96	BlongMCC10094ph1	100.000	2.34e-25	ACTGGTTTTCTAGGCAATTTCCAATCTAAC-GGGTGTTGCACTTTCATTTAGACAACGGG
Blong1-5B-spacer14	BlongAF08-2ph2	100.000	3.99e-08	GCCATTTCGTGAAAACCGTACGCTCAGA
Blong1-6B-spacer79	Blong157Fph1	100.000	1.24e-11	GTATCTGCCGGTGCGCATTACGGTCGGCACTAG
Blong17-1B-spacer121	BlongAM16-2ph1	100.000	3.21e-13	TAAGCGACCAGGACGCGAGGAC-CGCTAGCGAACGGT
Blong1888B-spacer101	Blon-gATCC15697.2ph1	100.000	1.24e-11	TTGATGATGAACCGGCCCCGATCACTCAAGA-GA
Blong1888B-spacer107	BreDRBB28ph4	100.000	3.69e-12	CAAAAACTCAACCAGCTCGTCCTGGAAAAA-TAAC
Blong1888B-spacer107	BlongMCC10124ph1	100.000	3.69e-12	CAAAAACTCAACCAGCTCGTCCTGGAAAAA-TAAC
Blong1888B-spacer107	BlongMCC10102ph1	100.000	3.69e-12	CAAAAACTCAACCAGCTCGTCCTGGAAAAA-TAAC
Blong1888B-spacer39	BreNRBB52ph5	100.000	3.69e-12	AGGTTCGAGCTTGAC-CATTCGCCCAGGTTCACGT
Blong1888B-spacer39	BreMCC1605ph1	100.000	3.69e-12	AGGTTCGAGCTTGAC-CATTCGCCCAGGTTCACGT
Blong1888B-spacer39	BreLMC520ph3	100.000	3.69e-12	AGGTTCGAGCTTGAC-CATTCGCCCAGGTTCACGT
Blong1888B-spacer39	BreJR01ph1	100.000	3.69e-12	AGGTTCGAGCTTGAC-

Spacer Name	Phage Name	Identity	E-value	Nucleotide Sequence
				CATTCGCCCAGGTTCACGT
Blong1888B-spacer39	BreDRBB28ph5	100.000	3.69e-12	AGGTTCGAGCTTGAC-CATTCGCCCAGGTTCACGT
Blong1888B-spacer39	BreDRBB26ph1	100.000	3.69e-12	AGGTTCGAGCTTGAC-CATTCGCCCAGGTTCACGT
Blong1888B-spacer39	BreBR3ph2	100.000	3.69e-12	AGGTTCGAGCTTGAC-CATTCGCCCAGGTTCACGT
Blong1888B-spacer39	Bre082W48ph1	100.000	3.69e-12	AGGTTCGAGCTTGAC-CATTCGCCCAGGTTCACGT
Blong1888B-spacer39	Bre017W439ph2	100.000	3.69e-12	AGGTTCGAGCTTGAC-CATTCGCCCAGGTTCACGT
Blong1888B-spacer39	BlongLO-K29bph1	100.000	3.69e-12	AGGTTCGAGCTTGAC-CATTCGCCCAGGTTCACGT
Blong1888B-spacer39	Blong-CECT7210.2ph3	100.000	3.69e-12	AGGTTCGAGCTTGAC-CATTCGCCCAGGTTCACGT
Blong1888B-spacer39	Blong-CCUG30698ph3	100.000	3.69e-12	AGGTTCGAGCTTGAC-CATTCGCCCAGGTTCACGT
Blong1888B-spacer39	BlongAPC1462ph1	100.000	3.69e-12	AGGTTCGAGCTTGAC-CATTCGCCCAGGTTCACGT
Blong1888B-spacer39	Blong72Bph1	100.000	3.69e-12	AGGTTCGAGCTTGAC-CATTCGCCCAGGTTCACGT
Blong1888B-spacer39	Blong157Fph1	100.000	3.69e-12	AGGTTCGAGCTTGAC-CATTCGCCCAGGTTCACGT
Blong1888B-spacer41	BlongMCC10100ph1	100.000	1.24e-11	CGCGCGTCATGGGACTCGATGACGAAGGGA-GAA
Blong1888B-spacer41	BlongMCC10079ph1	100.000	1.24e-11	CGCGCGTCATGGGACTCGATGACGAAGGGA-GAA
Blong1888B-spacer41	BlongMCC10077ph1	100.000	1.24e-11	CGCGCGTCATGGGACTCGATGACGAAGGGA-GAA
Blong1888B-spacer41	BlongMCC10073ph1	100.000	1.24e-11	CGCGCGTCATGGGACTCGATGACGAAGGGA-GAA
Blong1888B-spacer41	BlongMCC10064ph1	100.000	1.24e-11	CGCGCGTCATGGGACTCGATGACGAAGGGA-GAA
Blong1888B-spacer41	BlongMCC10044ph1	100.000	1.24e-11	CGCGCGTCATGGGACTCGATGACGAAGGGA-GAA
Blong1888B-spacer41	BlongMCC10038ph1	100.000	1.24e-11	CGCGCGTCATGGGACTCGATGACGAAGGGA-GAA
Blong1888B-spacer41	BlongLO-K29aph1	100.000	1.24e-11	CGCGCGTCATGGGACTCGATGACGAAGGGA-GAA
Blong1888B-spacer45	BreDRBB28ph4	100.000	3.69e-12	ATCGCACGCATGATCAG-CAGCCTGCCCCTCAAGG
Blong1888B-spacer45	BreBR-14ph1	100.000	3.69e-12	ATCGCACGCATGATCAG-CAGCCTGCCCCTCAAGG
Blong1888B-spacer45	BlongN3A01ph1	100.000	3.69e-12	ATCGCACGCATGATCAG-CAGCCTGCCCCTCAAGG
Blong1888B-spacer45	BlongMCC10124ph1	100.000	3.69e-12	ATCGCACGCATGATCAG-CAGCCTGCCCCTCAAGG
Blong1888B-spacer45	BlongMCC10102ph1	100.000	3.69e-12	ATCGCACGCATGATCAG-CAGCCTGCCCCTCAAGG
Blong1888B-spacer45	BlongMCC10052ph1	100.000	3.69e-12	ATCGCACGCATGATCAG-CAGCCTGCCCCTCAAGG
Blong1888B-spacer45	BlongMCC10008ph1	100.000	3.69e-12	ATCGCACGCATGATCAG-CAGCCTGCCCCTCAAGG
Blong1888B-spacer45	BlongE18ph4	100.000	3.69e-12	ATCGCACGCATGATCAG-CAGCCTGCCCCTCAAGG
Blong1888B-spacer45	BlongAPC1504ph1	100.000	3.69e-12	ATCGCACGCATGATCAG-

Spacer Name	Phage Name	Identity	E-value	Nucleotide Sequence
				CAGCCTGCCCCTCAAGG
Blong1888B-spacer47	Brelw01ph1	100.000	3.69e-12	CACTTGTCCTTGGTCTCTATGGGGTT-GCCGCCGC
Blong1888B-spacer47	BlongNCTC11817ph3	100.000	3.69e-12	CACTTGTCCTTGGTCTCTATGGGGTT-GCCGCCGC
Blong1888B-spacer49	Brelw01ph1	100.000	3.69e-12	AACGCTTCCTCGACCTGCTGGTCCCAC-CGTTCGA
Blong1888B-spacer49	BlongNCTC11817ph3	100.000	3.69e-12	AACGCTTCCTCGACCTGCTGGTCCCAC-CGTTCGA
Blong1888B-spacer53	Brelw01ph1	100.000	3.92e-12	CCGTGTGGGGTTGGTGCGGCTCATTTGAC-GAGGT
Blong1888B-spacer53	BlongNCTC11817ph3	100.000	3.92e-12	CCGTGTGGGGTTGGTGCGGCTCATTTGAC-GAGGT
Blong1888B-spacer65	BlongTF06-12ACph1	100.000	9.42e-14	TACAACATCCTTAACCAGA-TATTCAAGGCGGCGGTGG
Blong1888B-spacer65	BlongE18ph2	100.000	9.42e-14	TACAACATCCTTAACCAGA-TATTCAAGGCGGCGGTGG
Blong1888B-spacer65	Blon-gATCC15697.2ph1	100.000	9.42e-14	TACAACATCCTTAACCAGA-TATTCAAGGCGGCGGTGG
Blong1888B-spacer65	BlongAH1206ph2	100.000	9.42e-14	TACAACATCCTTAACCAGA-TATTCAAGGCGGCGGTGG
Blong1888B-spacer65	BlongAF08-2ph2	100.000	9.42e-14	TACAACATCCTTAACCAGA-TATTCAAGGCGGCGGTGG
Blong1888B-spacer83	Blon-gAM39-8ACph1	100.000	1.09e-12	TCGGCACGATGACGGCGGCCTCCTCAC-CGGCTTCG
Blong1890B-spacer42	Blong-CECT7210.2ph4	100.000	3.92e-12	GGTCACGACGGTGACAAGAGCGCCCAG-CATGTCG
Blong1897B-spacer131	Blon-gAM39-8ACph1	100.000	4.47e-11	TGGAGGATGCATCATGACGATGACGAGCGCCG
Blong1897B-spacer133	BlongU-MA3015ph1	100.000	4.47e-11	TCCAGCTCACCGAGTGGAAGGGGTAGCCGATG
Blong1897B-spacer133	BlongMCC10116ph1	100.000	4.47e-11	TCCAGCTCACCGAGTGGAAGGGGTAGCCGATG
Blong1897B-spacer133	BlongMCC10115ph1	100.000	4.47e-11	TCCAGCTCACCGAGTGGAAGGGGTAGCCGATG
Blong1897B-spacer133	BlongMCC10111ph1	100.000	4.47e-11	TCCAGCTCACCGAGTGGAAGGGGTAGCCGATG
Blong1897B-spacer133	BlongMCC10100ph1	100.000	4.47e-11	TCCAGCTCACCGAGTGGAAGGGGTAGCCGATG
Blong1897B-spacer133	BlongMCC10093ph1	100.000	4.47e-11	TCCAGCTCACCGAGTGGAAGGGGTAGCCGATG
Blong1897B-spacer133	BlongMCC10079ph1	100.000	4.47e-11	TCCAGCTCACCGAGTGGAAGGGGTAGCCGATG
Blong1897B-spacer133	BlongMCC10077ph1	100.000	4.47e-11	TCCAGCTCACCGAGTGGAAGGGGTAGCCGATG
Blong1897B-spacer133	BlongMCC10073ph1	100.000	4.47e-11	TCCAGCTCACCGAGTGGAAGGGGTAGCCGATG
Blong1897B-spacer133	BlongMCC10064ph1	100.000	4.47e-11	TCCAGCTCACCGAGTGGAAGGGGTAGCCGATG
Blong1897B-spacer133	BlongMCC10044ph1	100.000	4.47e-11	TCCAGCTCACCGAGTGGAAGGGGTAGCCGATG
Blong1897B-spacer133	BlongMCC10038ph1	100.000	4.47e-11	TCCAGCTCACCGAGTGGAAGGGGTAGCCGATG
Blong1897B-spacer133	BlongLO-K29aph1	100.000	4.47e-11	TCCAGCTCACCGAGTGGAAGGGGTAGCCGATG
Blong1897B-spacer20	BlongBBMN68ph4	100.000	1.39e-32	TGTGTCTGTTCAA-

Spacer Name	Phage Name	Identity	E-value	Nucleotide Sequence
9				TAGAAAGTGGGCCGTGTGAGCGTGTG-GATGTCTTCCATGTGAGCGGCAGGTTCTGGTCC
Blong1897B-spacer209	Blong35624ph1	98.276	3.91e-23	TGTGTCTGTTCAA-TAGAAAGTGGGCCGTGTGAGCGTGTG-GATGTCTTCCATGTGAGCG
Blong1897B-spacer210	BlongBBMN68ph4	100.000	1.37e-42	GACCGGCCAACGCCGAAAC-GGCCTGAAACCCGCCGCTCACATGGACTAAAAGAAACGCCGAAACGGCCTACTCCAACTT-GACAAAACACA
Blong1897B-spacer45	BreNRBB02ph1	100.000	5.49e-09	TGTACGGATATATGGCGGTTGCCGCCAC
Blong1897B-spacer78	BreJR01ph1	98.077	3.60e-20	GTTCGCTTCGGCCACCTTGGCCAG-CATGCCGCTGTTGTCCCCCTGCAGCAAC
Blong1897B-spacer78	BreDRBB28ph5	98.077	3.60e-20	GTTCGCTTCGGCCACCTTGGCCAG-CATGCCGCTGTTGTCCCCCTGCAGCAAC
Blong1897B-spacer78	BreDRBB26ph1	98.077	3.60e-20	GTTCGCTTCGGCCACCTTGGCCAG-CATGCCGCTGTTGTCCCCCTGCAGCAAC
Blong1897B-spacer80	BreJR01ph1	98.077	3.60e-20	TGCGGCGATGGCCATCG-CATAGTCGAACTGGGTCTTGTCCAGATCGAG-TTTG
Blong1897B-spacer80	BreDRBB28ph5	98.077	3.60e-20	TGCGGCGATGGCCATCG-CATAGTCGAACTGGGTCTTGTCCAGATCGAG-TTTG
Blong1897B-spacer80	BreDRBB26ph1	98.077	3.60e-20	TGCGGCGATGGCCATCG-CATAGTCGAACTGGGTCTTGTCCAGATCGAG-TTTG
Blong1897B-spacer87	BlongAF08-2ph2	100.000	2.04e-10	TCGTCGTCAACGTACGGTTTTCACGAAATGG
Blong1898B-spacer31	Blong157Fph1	100.000	5.49e-09	AGGTCGAGCGCCTCCTGCCATCCGGCCT
Blong1898B-spacer39	Blong-CECT7210.2ph4	100.000	5.49e-09	CGTTCCCCAGTGGGGTGAGTGTGATGCC
Blong2-2B-spacer18	BlongTM01-1ph1	100.000	5.49e-09	CGGTGCCCGGCAGGTCGTCAACGATCTG
Blong2-2B-spacer18	BlongMCC10101ph1	100.000	5.49e-09	CGGTGCCCGGCAGGTCGTCAACGATCTG
Blong2-2B-spacer18	BlongGT15ph3	100.000	5.49e-09	CGGTGCCCGGCAGGTCGTCAACGATCTG
Blong2-2B-spacer18	BlongDS15_3ph1	100.000	5.49e-09	CGGTGCCCGGCAGGTCGTCAACGATCTG
Blong2-2B-spacer18	BlongBB-79ph1	100.000	5.49e-09	CGGTGCCCGGCAGGTCGTCAACGATCTG
Blong2-2B-spacer18	BlongAF30-12ph2	100.000	5.49e-09	CGGTGCCCGGCAGGTCGTCAACGATCTG
Blong239-2-spacer10	BreSC95ph1	100.000	4.47e-11	TCGCCCGAAGCGACGCCGCCGGCATGCCCGGC
Blong239-2-spacer131	BlongAM16-2ph1	100.000	4.47e-11	TGCCTTCCACCTTCGCGAGCATCAACGACGCC
Blong239-2-spacer267	BlongTF01-22ph1	100.000	4.47e-11	CCGAGCTTGTCCTTGACCCAGTTGAGCGCGTC
Blong239-2-spacer28	BreSC95ph1	100.000	4.47e-11	CTGGCCGCGATGCGGAACGCGGCCGCGGCGTC
Blong239-2-spacer303	Blong-CECT7210.2ph3	100.000	5.79e-10	CGGACGAGGATCTGAGTTTCGAACTGGCGG
Blong35624-spacer102	Blon-gATCC15697.2ph1	100.000	2.08e-09	CCGCCTTGTGTTTCCACCCGTCCGGCGCG
Blong35624-spacer122	Brelw01ph1	100.000	1.61e-10	TGCCGCAGTATTGGCATGGTTCGGTGGTGAT
Blong35624-spacer126	BreUMB0089ph2	100.000	1.09e-12	GAGGCCACGTCCTTGGCGAGGTCCTT-GCCCTGGGT
Blong35624-spacer126	BlongMCC10130ph1	100.000	1.09e-12	GAGGCCACGTCCTTGGCGAGGTCCTT-GCCCTGGGT
Blong35624-spacer126	BlongMCC10081ph2	100.000	1.41e-11	GGCCACGTCCTTGGCGAGGTCCTTGCCCTGGGT
Blong35624-spacer128	BreLMC520ph3	100.000	1.15e-12	GGGCGGAAGCTCAGGTTGTTGAACATGCAG-TCCGA

Spacer Name	Phage Name	Identity	E-value	Nucleotide Sequence
Blong35624-spacer128	BreJR01ph1	100.000	1.15e-12	GGGCGGAAGCTCAGGTTGTTGAACATGCAG-TCCGA
Blong35624-spacer128	BreDRBB28ph5	100.000	1.15e-12	GGGCGGAAGCTCAGGTTGTTGAACATGCAG-TCCGA
Blong35624-spacer128	BreDRBB26ph1	100.000	1.15e-12	GGGCGGAAGCTCAGGTTGTTGAACATGCAG-TCCGA
Blong35624-spacer128	BreBR3ph2	100.000	1.15e-12	GGGCGGAAGCTCAGGTTGTTGAACATGCAG-TCCGA
Blong35624-spacer128	Bre082W48ph1	100.000	1.15e-12	GGGCGGAAGCTCAGGTTGTTGAACATGCAG-TCCGA
Blong35624-spacer128	Blong-CECT7210.2ph3	100.000	1.15e-12	GGGCGGAAGCTCAGGTTGTTGAACATGCAG-TCCGA
Blong35624-spacer130	Blong-CECT7210.2ph3	100.000	1.24e-11	GCGCTGATGATTGGGCCGATCTGCTCGCG-GAAG
Blong35624-spacer130	Blong-CCUG30698ph3	100.000	1.24e-11	GCGCTGATGATTGGGCCGATCTGCTCGCG-GAAG
Blong35624-spacer130	Blong72Bph1	100.000	1.24e-11	GCGCTGATGATTGGGCCGATCTGCTCGCG-GAAG
Blong35624-spacer130	Blong157Fph1	100.000	1.24e-11	GCGCTGATGATTGGGCCGATCTGCTCGCG-GAAG
Blong35624-spacer132	BlongN3A01ph1	100.000	3.69e-12	CCGCGTTGCTGATGCCCCACAATT-GGCCGTTCCA
Blong35624-spacer136	BreUMB0089ph2	100.000	1.24e-11	TTCGGGCACGATCTGGCAGTCGCAGTCGTCGTG
Blong35624-spacer136	BlongMCC10130ph1	100.000	1.24e-11	TTCGGGCACGATCTGGCAGTCGCAGTCGTCGTG
Blong35624-spacer136	BlongBifido_06ph1	100.000	1.24e-11	TTCGGGCACGATCTGGCAGTCGCAGTCGTCGTG
Blong35624-spacer152	BlongBifido_06ph1	100.000	1.09e-12	AAACCGTTCTGGTTCAGTTCTATGACCTTCTT-GTT
Blong35624-spacer156	BlongBifido_06ph1	100.000	4.47e-11	CTCAATGTCACGGCACCATGTACCGGGCAGGG
Blong35624-spacer160	BlongVKP-MAc-1636ph1	100.000	3.69e-12	TCTGCGGCCGGTTCTCCACACGGTTCAC-GGTCGT
Blong35624-spacer160	BlongMCC10130ph1	100.000	3.69e-12	TCTGCGGCCGGTTCTCCACACGGTTCAC-GGTCGT
Blong35624-spacer160	BlongMCC10081ph2	100.000	3.69e-12	TCTGCGGCCGGTTCTCCACACGGTTCAC-GGTCGT
Blong35624-spacer160	BlongBifido_06ph1	100.000	3.69e-12	TCTGCGGCCGGTTCTCCACACGGTTCAC-GGTCGT
Blong35624-spacer168	BlongTM01-1ph1	100.000	3.69e-12	TCAC-GGTGCCGTCCAGGCCTCTCAATTTCAGCCA
Blong35624-spacer168	BlongMCC10101ph1	100.000	3.69e-12	TCAC-GGTGCCGTCCAGGCCTCTCAATTTCAGCCA
Blong35624-spacer168	BlongGT15ph3	100.000	3.69e-12	TCAC-GGTGCCGTCCAGGCCTCTCAATTTCAGCCA
Blong35624-spacer168	BlongDS15_3ph1	100.000	3.69e-12	TCAC-GGTGCCGTCCAGGCCTCTCAATTTCAGCCA
Blong35624-spacer168	BlongBB-79ph1	100.000	3.69e-12	TCAC-GGTGCCGTCCAGGCCTCTCAATTTCAGCCA
Blong35624-spacer168	BlongAF30-12ph2	100.000	3.69e-12	TCAC-GGTGCCGTCCAGGCCTCTCAATTTCAGCCA
Blong35624-spacer170	BlongAM16-2ph1	100.000	1.24e-11	GTCTGGTAGGCGCTGTAACGGGTGTTCGTGTCG
Blong35624-spacer174	BreUMB0089ph2	100.000	3.21e-13	TTGTTGTCCTT-GGCGCGTTTCTCCCATTCGCGGGAA

Spacer Name	Phage Name	Identity	E-value	Nucleotide Sequence
Blong35624-spacer174	BlongMCC10130ph1	100.000	3.21e-13	TTGTTGTCCTT-GGCGCGTTTCTCCCATTCGCGGGAA
Blong35624-spacer174	BlongMCC10081ph2	100.000	3.21e-13	TTGTTGTCCTT-GGCGCGTTTCTCCCATTCGCGGGAA
Blong35624-spacer174	BlongBifido_06ph1	100.000	3.21e-13	TTGTTGTCCTT-GGCGCGTTTCTCCCATTCGCGGGAA
Blong35624-spacer174	BifPRI1ph2	100.000	3.21e-13	TTGTTGTCCTT-GGCGCGTTTCTCCCATTCGCGGGAA
Blong35624-spacer176	BlongVKP-MAc-1636ph1	100.000	1.24e-11	AAGGCTGGACGTGACGACGGACGCGACCTT-GCT
Blong35624-spacer182	BlongTM01-1ph1	100.000	4.47e-11	CACTCGTCCAGCAGCCGAGGGCTTCGTGGCGT
Blong35624-spacer192	BlongAM16-2ph1	100.000	1.24e-11	GAAC-GCGCGCCGCCAGCCGGCCGGGTCGTCCGC
Blong35624-spacer196	BlongAH1206ph2	100.000	3.69e-12	GTTGATGGCTATCACTATACGCGGTCCAA-TACTG
Blong35624-spacer202	BlongSu859ph1	100.000	3.69e-12	CGGTGTTGGCCTGCAC-GGTGCTCATCTGCTGGTC
Blong35624-spacer206	BlongAM16-2ph1	100.000	3.21e-13	TTGGGCATCCCGGCGTCGTT-GATGCTCGCGAAGGTG
Blong35624-spacer218	BlongAH1206ph2	100.000	5.07e-11	TTCGCGGTATCACTACCGCAAGCGGACAGGGT
Blong35624-spacer220	Blong157Fph1	100.000	1.24e-11	ACCGGCCTTGTTCGGGTTGCCGGGGGTGATCTG
Blong35624-spacer222	BifS17ph1	100.000	1.24e-11	CATCACGTCGGAGATGGTCTGCTGGTCGGCGTT
Blong35624-spacer222	Blong157Fph1	100.000	4.47e-11	ATCACGTCGGAGATGGTCTGCTGGTCGGCGTT
Blong35624-spacer240	Blon-gAM39-8ACph1	100.000	3.69e-12	GACAGGTCAACAACAACTGCCTCGTGTCCGGCGT
Blong35624-spacer248	BlongE18ph4	100.000	1.24e-11	ATCGCGTCTCTCAAGGAGGCCCAAA-GCATCTGG
Blong35624-spacer256	BlongMCC10130ph1	100.000	1.09e-12	AGCATGTCGGTGTGCGGCTG-CATGGTCATCTGCGT
Blong35624-spacer256	BlongMCC10081ph2	100.000	1.09e-12	AGCATGTCGGTGTGCGGCTG-CATGGTCATCTGCGT
Blong35624-spacer256	BlongBifido_06ph1	100.000	1.09e-12	AGCATGTCGGTGTGCGGCTG-CATGGTCATCTGCGT
Blong35624-spacer256	BifPRI1ph2	100.000	1.09e-12	AGCATGTCGGTGTGCGGCTG-CATGGTCATCTGCGT
Blong35624-spacer262	BlongMCC10040ph2	100.000	3.21e-13	GATTGTCTCGTTGACGGATGTGTTGCTGAC-CTGAGT
Blong35624-spacer296	BifPRI1ph2	100.000	3.69e-12	CCGCCGCAAATGCAAGGCCAGACACATCG-CAGCC
Blong35624-spacer298	BlongMCC10130ph1	100.000	1.09e-12	TCGGGCATAAAAAAC-CACCCGTGCGGGTGGTTGGG
Blong35624-spacer298	BlongBifido_06ph1	100.000	1.09e-12	TCGGGCATAAAAAAC-CACCCGTGCGGGTGGTTGGG
Blong35624-spacer298	BifPRI1ph2	100.000	1.09e-12	TCGGGCATAAAAAAC-CACCCGTGCGGGTGGTTGGG
Blong35624-spacer322	BreNRBB02ph1	100.000	3.69e-12	CCGTGGCGCGCCGCTCTCGCATACTGGGCGG-CAT
Blong35624-spacer326	BreNRBB02ph1	100.000	1.24e-11	GTTGGCTTCGGCGCTTACCCTTGGCGGTGACGT
Blong35624-spacer344	BadAF21-27ph1	100.000	1.24e-11	CGACCGCCATCGGAATCCCAACCTACGAA-TAAG

Spacer Name	Phage Name	Identity	E-value	Nucleotide Sequence
Blong35624-spacer344	Bad1001271st1_A4ph1	100.000	1.24e-11	CGACCGCCATCGGAATCCCAACCTACGAA-TAAG
Blong35624-spacer346	BadAF21-27ph1	100.000	1.24e-11	GTTCGCCGCATTGGCGGCGGCGTTGAA-GCCGAT
Blong35624-spacer348	BadAF21-27ph1	100.000	3.69e-12	CCGGCGGGGTCCAGGCCACTCCGGTCG-GAGCCAC
Blong35624-spacer350	BadAF21-27ph1	100.000	1.09e-12	GCCATCGCGCGATATGACGGCCAGCCG-GACAGGCA
Blong35624-spacer354	BadAF21-27ph1	100.000	3.69e-12	TATCCGCTCGCCCAAGAACGGCGGCGAC-GAACTC
Blong35624-spacer42	BlongSu859ph1	100.000	3.69e-12	AGCGCGGTCATGGTTTCCTCCTTAAAGGATCGTT
Blong35624-spacer42	BadP2P3ph1	100.000	3.69e-12	AGCGCGGTCATGGTTTCCTCCTTAAAGGATCGTT
Blong35624-spacer72	BlongMCC10040ph2	100.000	2.36e-09	ATCTTCTGGTTCGCGCCGTCGCGTCCGCC
Blong35624-spacer72	Blong-CCUG30698ph3	100.000	2.36e-09	ATCTTCTGGTTCGCGCCGTCGCGTCCGCC
Blong35624-spacer76	BlongEK5ph1	100.000	5.07e-11	GACACCATCATGGACGCCATCATCCCGACCAT
Blong35624-spacer82	BlongVKP-MAc-1636ph1	100.000	1.09e-12	TGACCGGTCTTGCCGTTTACCTGAC-CGTCCAAGGG
Blong35624-spacer82	BlongMCC10130ph1	100.000	1.09e-12	TGACCGGTCTTGCCGTTTACCTGAC-CGTCCAAGGG
Blong35624-spacer82	BlongBifido_06ph1	100.000	1.09e-12	TGACCGGTCTTGCCGTTTACCTGAC-CGTCCAAGGG
Blong35624-spacer90	Blong-CECT7210.2ph3	100.000	1.24e-11	CGCAACGCCTCTGGGAACGAGCCCTAA-TAGCCT
Blong35624-spacer90	Blong-CCUG30698ph3	100.000	1.24e-11	CGCAACGCCTCTGGGAACGAGCCCTAA-TAGCCT
Blong35624-spacer90	Blon-gAM39-8ACph1	100.000	1.24e-11	CGCAACGCCTCTGGGAACGAGCCCTAA-TAGCCT
Blong35624-spacer90	Blong157Fph1	100.000	1.24e-11	CGCAACGCCTCTGGGAACGAGCCCTAA-TAGCCT
Blong35624-spacer94	Blong-CCUG30698ph3	100.000	3.69e-12	GCAACATCACCAC-CATCTGGGTGCCGGCGATCAA
Blong35624-spacer94	Blong72Bph1	100.000	3.69e-12	GCAACATCACCAC-CATCTGGGTGCCGGCGATCAA
Blong35624-spacer94	Blong157Fph1	100.000	3.69e-12	GCAACATCACCAC-CATCTGGGTGCCGGCGATCAA
Blong35B-spacer31	BlongMCC10081ph1	100.000	5.49e-09	AGGTCGAGCGCTTCCCGCCAGCCGGCCT
Blong35B-spacer31	Blong-CECT7210.2ph3	100.000	5.49e-09	AGGTCGAGCGCTTCCCGCCAGCCGGCCT
Blong35B-spacer31	Blong-CCUG30698ph3	100.000	5.49e-09	AGGTCGAGCGCTTCCCGCCAGCCGGCCT
Blong44B-spacer110	BlongTF06-12ACph1	100.000	5.49e-09	ACCATCGAGGAAGGCCGAGCCGCCTATC
Blong7-1B-spacer29	BlongAH1206ph2	100.000	5.49e-09	TTCCAAGCGGCGATATTGTATTGCAACT
Blong7-1B-spacer43	BlongMCC10130ph1	100.000	5.49e-09	AGCATGGTGCGCACCGCCGTGTCGGTGA
Blong7-1B-spacer43	BlongMCC10081ph2	100.000	5.49e-09	AGCATGGTGCGCACCGCCGTGTCGGTGA
Blong7-1B-spacer43	BlongBifido_06ph1	100.000	5.49e-09	AGCATGGTGCGCACCGCCGTGTCGGTGA
Blon-gAF04-13-spacer91	BlongMCC10116ph1	100.000	8.05e-15	GCCCTCGCCCAC-GGTTCCAGCGCCGCCCTCGAGCGCGCC
Blon-	BlongMCC10093ph	100.000	8.05e-15	GCCCTCGCCCAC-

Spacer Name	Phage Name	Identity	E-value	Nucleotide Sequence
gAF04-13-spacer91	1			GGTTCCAGCGCCGCCCTCGAGCGCGCC
Blon-gAF04-13-spacer91	BlongMCC10079ph1	100.000	8.05e-15	GCCCTCGCCCAC-GGTTCCAGCGCCGCCCTCGAGCGCGCC
Blon-gAF04-13-spacer91	BlongMCC10077ph1	100.000	8.05e-15	GCCCTCGCCCAC-GGTTCCAGCGCCGCCCTCGAGCGCGCC
Blon-gAF04-13-spacer91	BlongMCC10073ph1	100.000	8.05e-15	GCCCTCGCCCAC-GGTTCCAGCGCCGCCCTCGAGCGCGCC
Blon-gAF04-13-spacer91	BlongMCC10064ph1	100.000	8.05e-15	GCCCTCGCCCAC-GGTTCCAGCGCCGCCCTCGAGCGCGCC
Blon-gAF04-13-spacer91	BlongMCC10044ph1	100.000	8.05e-15	GCCCTCGCCCAC-GGTTCCAGCGCCGCCCTCGAGCGCGCC
Blon-gAF04-13-spacer91	BlongMCC10038ph1	100.000	8.05e-15	GCCCTCGCCCAC-GGTTCCAGCGCCGCCCTCGAGCGCGCC
Blon-gAF04-13-spacer91	BlongLO-K29aph1	100.000	8.05e-15	GCCCTCGCCCAC-GGTTCCAGCGCCGCCCTCGAGCGCGCC
Blon-gAF26-10-spacer113	BlongBifido_06ph1	100.000	4.47e-11	AGGGGAACGTCCATCGAATGCCCGCCCACTGG
Blon-gAF26-10-spacer117	BlongEK5ph1	100.000	4.47e-11	TCCTTGAGGGCCACGAGCGCCTTCATCACGTC
Blon-gAF26-10-spacer173	Blong-BIC1307292462ph1	100.000	4.47e-11	GCCTTCGCCTGAAACATGTGGTCGGCCTCGTA
Blon-gAF26-10-spacer173	Blon-gATCC15697.2ph1	100.000	4.47e-11	GCCTTCGCCTGAAACATGTGGTCGGCCTCGTA
Blon-gAF26-10-spacer183	BlongU-MA3015ph1	100.000	4.47e-11	GGTTCGAAAATCGGCAAATCGTTCGGCGGCAG
Blon-gAF26-10-spacer183	BlongMCC10116ph1	100.000	4.47e-11	GGTTCGAAAATCGGCAAATCGTTCGGCGGCAG
Blon-gAF26-10-spacer183	BlongMCC10115ph1	100.000	4.47e-11	GGTTCGAAAATCGGCAAATCGTTCGGCGGCAG
Blon-gAF26-10-spacer183	BlongMCC10111ph1	100.000	4.47e-11	GGTTCGAAAATCGGCAAATCGTTCGGCGGCAG
Blon-gAF26-10-spacer183	BlongMCC10100ph1	100.000	4.47e-11	GGTTCGAAAATCGGCAAATCGTTCGGCGGCAG
Blon-gAF26-10-spacer183	BlongMCC10093ph1	100.000	4.47e-11	GGTTCGAAAATCGGCAAATCGTTCGGCGGCAG
Blon-gAF26-10-spacer183	BlongMCC10077ph1	100.000	4.47e-11	GGTTCGAAAATCGGCAAATCGTTCGGCGGCAG
Blon-gAF26-10-spacer183	BlongMCC10040ph2	100.000	4.47e-11	GGTTCGAAAATCGGCAAATCGTTCGGCGGCAG
Blon-gAF26-10-spacer183	BlongMCC10038ph1	100.000	4.47e-11	GGTTCGAAAATCGGCAAATCGTTCGGCGGCAG
Blon-gAF26-10-spacer183	BlongLO-K29aph1	100.000	4.47e-11	GGTTCGAAAATCGGCAAATCGTTCGGCGGCAG
Blon-gAF26-10-spacer185	BlongMCC10116ph1	100.000	4.47e-11	GGACAGACCAGCATCAAGTTCAACGACGCGTT
Blon-gAF26-10-spacer185	BlongMCC10115ph1	100.000	4.47e-11	GGACAGACCAGCATCAAGTTCAACGACGCGTT
Blon-gAF26-10-spacer185	BlongMCC10111ph1	100.000	4.47e-11	GGACAGACCAGCATCAAGTTCAACGACGCGTT
Blon-gAF26-10-spacer185	BlongMCC10093ph1	100.000	4.47e-11	GGACAGACCAGCATCAAGTTCAACGACGCGTT
Blon-gAF26-10-spacer185	BlongMCC10077ph1	100.000	4.47e-11	GGACAGACCAGCATCAAGTTCAACGACGCGTT
Blon-gAF26-10-spacer185	BlongMCC10073ph1	100.000	4.47e-11	GGACAGACCAGCATCAAGTTCAACGACGCGTT
Blon-	BlongMCC10064ph	100.000	4.47e-11	GGACAGACCAGCATCAAGTTCAACGACGCGTT

Spacer Name	Phage Name	Identity	E-value	Nucleotide Sequence
gAF26-10-spacer185	1			
Blon-gAF26-10-spacer185	BlongMCC10044ph1	100.000	4.47e-11	GGACAGACCAGCATCAAGTTCAACGACGCGTT
Blon-gAF26-10-spacer185	BlongMCC10038ph1	100.000	4.47e-11	GGACAGACCAGCATCAAGTTCAACGACGCGTT
Blon-gAF26-10-spacer185	BlongLO-K29aph1	100.000	4.47e-11	GGACAGACCAGCATCAAGTTCAACGACGCGTT
Blon-gAF26-10-spacer19	BlongTF01-22ph1	100.000	6.63e-11	TACTTCGGCGTCAAGGCCGACACCGACGG-CAAC
Blon-gAF26-10-spacer231	BlongVKP-MAc-1636ph1	100.000	4.47e-11	TATGACCTTCAGGCCGATCCGAAGGGCAACGC
Blon-gAF26-10-spacer231	BlongMCC10130ph1	100.000	4.47e-11	TATGACCTTCAGGCCGATCCGAAGGGCAACGC
Blon-gAF26-10-spacer231	BlongBifido_06ph1	100.000	4.47e-11	TATGACCTTCAGGCCGATCCGAAGGGCAACGC
Blon-gAF26-10-spacer233	BlongVKP-MAc-1636ph1	100.000	4.47e-11	GTGGCCGCGAACGACATGGGCGATGGTGTCAC
Blon-gAF26-10-spacer233	BlongMCC10130ph1	100.000	4.47e-11	GTGGCCGCGAACGACATGGGCGATGGTGTCAC
Blon-gAF26-10-spacer251	BreSC95ph1	100.000	4.47e-11	GTGCACACCGCGTACCAGCTGCGATTTCCCGC
Blon-gAF26-10-spacer42	BlongTF01-22ph1	100.000	4.47e-11	TTGGGTAAGATCGGCTCCCTGTTCGGCTCGTT
Blon-gAF26-10-spacer53	Blong-CECT7210.2ph3	100.000	4.47e-11	GTCATGGTCCCGTCCGCATGTATGTTGATGGT
Blon-gAF27-1BH-spacer103	BlongMCC10040ph2	100.000	4.47e-11	GGTACCGTAAGTGGAGTTAATCAGCGTCAGCA
Blon-gAF27-1BH-spacer103	Blong-CCUG30698ph3	100.000	4.47e-11	GGTACCGTAAGTGGAGTTAATCAGCGTCAGCA
Blon-gAF27-1BH-spacer222	BlongIndicaph2	100.000	3.91e-23	GTGTTTTGCTTCAATGTTT-GTTCGACAGGTTCGGCAGTGTTTTTCCGTAG-TGTGG
Blon-gAF27-1BH-spacer222	BlongBBMN68ph4	100.000	1.41e-22	TGTTTTGCTTCAATGTTT-GTTCGACAGGTTCGGCAGTGTTTTTCCGTAG-TGTGG
Blon-gAF27-1BH-spacer222	BlongBBMN68ph1	100.000	1.41e-22	TGTTTTGCTTCAATGTTT-GTTCGACAGGTTCGGCAGTGTTTTTCCGTAG-TGTGG
Blon-gAF35-13AC-spacer17	BreJR01ph1	100.000	1.76e-23	CAAACTCGATCTGGACAAGACCCAGTTCGAT-TATGCGATGGCCATCGCCGCAGGT
Blon-gAF35-13AC-spacer17	BreDRBB28ph5	100.000	1.76e-23	CAAACTCGATCTGGACAAGACCCAGTTCGAT-TATGCGATGGCCATCGCCGCAGGT
Blon-gAF35-13AC-spacer17	BreDRBB26ph1	100.000	1.76e-23	CAAACTCGATCTGGACAAGACCCAGTTCGAT-TATGCGATGGCCATCGCCGCAGGT
Blon-gAF35-13AC-spacer19	BreJR01ph1	98.182	8.18e-22	GTTGCTGCAGGGG-GACAACAGCGACATGCTGGCCAAGGTGGCCAAAGCGAACGGC
Blon-gAF35-13AC-spacer19	BreDRBB28ph5	98.182	8.18e-22	GTTGCTGCAGGGG-GACAACAGCGACATGCTGGCCAAGGTGGCCAAAGCGAACGGC
Blon-	BreDRBB26ph1	98.182	8.18e-22	GTTGCTGCAGGGG-

Spacer Name	Phage Name	Identity	E-value	Nucleotide Sequence
gAF35-13AC-spacer19				GACAACAGCGACATGCTGGCCAAGGTGGCCAAAGCGAACGGC
Blon-gAF35-13AC-spacer21	BreJR01ph1	100.000	3.77e-48	CTATATCTATGCAAACGAC-GATCAGGCCATCGGAG-TGCTCCAAGGCCTGAACAACATGCAGATCGCGGACAAATGGTTCACCATCCATGGCAAATAC-GAG
Blon-gAF35-13AC-spacer21	BreDRBB28ph5	99.000	1.76e-46	CTATATCTATGCAAACGAC-GATCAGGCCATCGGAG-TGCTCCAAGGCCTGAACAACATGCAGATCGCGGACAAATGGTTCACCATCCATGGCAAATAC-GAG
Blon-gAF35-13AC-spacer21	BreDRBB26ph1	99.000	1.76e-46	CTATATCTATGCAAACGAC-GATCAGGCCATCGGAG-TGCTCCAAGGCCTGAACAACATGCAGATCGCGGACAAATGGTTCACCATCCATGGCAAATAC-GAG
Blon-gAGR2137-spacer27	BlongSu859ph1	100.000	1.50e-10	CCGACTCGAGCAGTTTCTTCGCGCGCGCGAT
Blon-gAM11-2-spacer60	BlongE18ph4	100.000	1.67e-09	CCACTGATGAAAGACACGGACAAGCTCAC
Blon-gAM11-2-spacer60	Blon-gAF34-9ACph1	100.000	1.67e-09	CCACTGATGAAAGACACGGACAAGCTCAC
Blon-gAM11-2-spacer66	Blong-CECT7210.2ph3	100.000	5.49e-09	GTGAGCGTATACAGGGCATGCCCGTCAT
Blon-gAM11-2-spacer66	Blong-CCUG30698ph3	100.000	5.49e-09	GTGAGCGTATACAGGGCATGCCCGTCAT
Blon-gAM11-2-spacer66	Blong157Fph1	100.000	5.49e-09	GTGAGCGTATACAGGGCATGCCCGTCAT
Blon-gAM20-39-spacer10	BlongJih1ph1	98.990	6.31e-46	CAACGACCGGCCAC-GCAAAACCCTCGGGTTCATGAAACCAA-GCGAGAAGATCATCGAACTGCTCGACGAC-GCGTGATAACCTCAACAACCGACAACGTG
Blon-gAM20-39-spacer10	Blong-CECT7210.2ph4	98.990	6.31e-46	CAACGACCGGCCAC-GCAAAACCCTCGGGTTCATGAAACCAA-GCGAGAAGATCATCGAACTGCTCGACGAC-GCGTGATAACCTCAACAACCGACAACGTG
Blon-gAM20-39-spacer10	Blong157Fph1	98.990	6.31e-46	CAACGACCGGCCAC-GCAAAACCCTCGGGTTCATGAAACCAA-GCGAGAAGATCATCGAACTGCTCGACGAC-GCGTGATAACCTCAACAACCGACAACGTG
Blon-gAM20-39-spacer10	Blong157Fph1	98.990	6.31e-46	CAACGACCGGCCAC-GCAAAACCCTCGGGTTCATGAAACCAA-GCGAGAAGATCATCGAACTGCTCGACGAC-GCGTGATAACCTCAACAACCGACAACGTG
Blon-gAM20-39-spacer11	BlongJih1ph1	100.000	4.47e-11	CCATGGAAGGCCGCTCAAACCTCAGGTGTTGC
Blon-gAM20-39-spacer11	Blong-CECT7210.2ph4	100.000	4.47e-11	CCATGGAAGGCCGCTCAAACCTCAGGTGTTGC
Blon-gAM20-39-spacer13	Blong157Fph1	100.000	4.47e-11	CCATGGAAGGCCGCTCAAACCCCAGGTGTTGC
Blon-gAM20-39-spacer13	Blong157Fph1	100.000	4.47e-11	CCATGGAAGGCCGCTCAAACCCCAGGTGTTGC
Blon-gAM34-3-spacer105	BlongVKP-MAc-1636ph1	100.000	1.24e-11	GAGAGGCACGCCAACGATGCCTTCGGGCAC-GTC
Blon-	BlongMCC10130ph	100.000	1.24e-11	GAGAGGCACGCCAACGATGCCTTCGGGCAC-

Spacer Name	Phage Name	Identity	E-value	Nucleotide Sequence
gAM34-3-spacer105	1			GTC
Blon-gAM34-3-spacer149	BlongBifido_03ph1	100.000	1.15e-12	TTTCGACGGAGCCAAATCGGTCGG-CAAGTCCAGTG
Blon-gAM34-3-spacer167	BlongTF07-39ph1	100.000	1.24e-11	CCCATGAGCGGGTGCCGTTTCGGCCGTCTCCGG
Blon-gAM34-3-spacer167	BlongTF07-31ph1	100.000	1.24e-11	CCCATGAGCGGGTGCCGTTTCGGCCGTCTCCGG
Blon-gAM34-3-spacer167	BlongTF06-12ACph1	100.000	1.24e-11	CCCATGAGCGGGTGCCGTTTCGGCCGTCTCCGG
Blon-gAM34-3-spacer167	BlongE18ph2	100.000	1.24e-11	CCCATGAGCGGGTGCCGTTTCGGCCGTCTCCGG
Blon-gAM34-3-spacer167	BlongAH1206ph2	100.000	1.24e-11	CCCATGAGCGGGTGCCGTTTCGGCCGTCTCCGG
Blon-gAM34-3-spacer167	BlongAF08-2ph2	100.000	1.24e-11	CCCATGAGCGGGTGCCGTTTCGGCCGTCTCCGG
Blon-gAM34-3-spacer197	BlongDS9_3ph2	100.000	3.21e-13	CGCCAAGCAGATCAGCGAATCC-TATGGCCAATTCAT
Blon-gAM34-3-spacer203	Blon-gATCC15697.2ph1	100.000	1.24e-11	GCTGATTTTGAGTCCCGTTGGACATGCGAGAAC
Blon-gAM34-3-spacer209	BreNRBB52ph5	100.000	8.05e-15	AAGCGCTTGTCCTGCTCCTCACGGGAGA-GACTTTTGAAA
Blon-gAM34-3-spacer209	Blong-CECT7210.2ph3	100.000	8.05e-15	AAGCGCTTGTCCTGCTCCTCACGGGAGA-GACTTTTGAAA
Blon-gAM34-3-spacer211	BlongMCC10115ph1	100.000	1.09e-12	TTGCGGCCGAACTTGTAGATGGTCTCCTGA-TAGGC
Blon-gAM34-3-spacer211	BlongMCC10077ph1	100.000	1.09e-12	TTGCGGCCGAACTTGTAGATGGTCTCCTGA-TAGGC
Blon-gAM34-3-spacer231	BlongMCC10115ph1	100.000	1.61e-10	ACCGCTACGCGGACTGCTATACGGGTTTGCA
Blon-gAM34-3-spacer75	BreUMB0089ph2	100.000	1.24e-11	ACGGTGGACGTGCTGGTGTGCGACGAGGCG-CAG
Blon-gAM34-3-spacer75	BlongMCC10130ph1	100.000	1.24e-11	ACGGTGGACGTGCTGGTGTGCGACGAGGCG-CAG
Blon-gAM34-3-spacer75	BlongMCC10081ph2	100.000	1.24e-11	ACGGTGGACGTGCTGGTGTGCGACGAGGCG-CAG
Blon-gAM34-3-spacer75	BlongBifido_06ph1	100.000	1.24e-11	ACGGTGGACGTGCTGGTGTGCGACGAGGCG-CAG
Blon-gAM34-3-spacer75	BifPRI1ph2	100.000	1.24e-11	ACGGTGGACGTGCTGGTGTGCGACGAGGCG-CAG
Blon-gAM39-10AC-spacer48	BlongMCC10130ph1	100.000	1.09e-12	TGAACCTCGCCCAGCAG-GATTGCGTCGCCATGCGA
Blon-gAM39-10AC-spacer48	BlongBifido_06ph1	100.000	1.09e-12	TGAACCTCGCCCAGCAG-GATTGCGTCGCCATGCGA
Blon-gAM39-10AC-spacer58	BlongMCC10116ph1	100.000	1.09e-12	CTCTCGCTGGCAAACAACCAA-TAGAAAGGATGATC
Blon-gAM39-10AC-spacer58	BlongMCC10100ph1	100.000	1.09e-12	CTCTCGCTGGCAAACAACCAA-TAGAAAGGATGATC
Blon-gAM39-10AC-spacer58	BlongMCC10093ph1	100.000	1.09e-12	CTCTCGCTGGCAAACAACCAA-TAGAAAGGATGATC
Blon-gAM39-10AC-spacer	BlongMCC10079ph1	100.000	1.09e-12	CTCTCGCTGGCAAACAACCAA-TAGAAAGGATGATC

Spacer Name	Phage Name	Identity	E-value	Nucleotide Sequence
58				
Blon-gAM39-10AC-spacer 58	BlongMCC10077ph1	100.000	1.09e-12	CTCTCGCTGGCAAACAACCAA-TAGAAAGGATGATC
Blon-gAM39-10AC-spacer 58	BlongMCC10073ph1	100.000	1.09e-12	CTCTCGCTGGCAAACAACCAA-TAGAAAGGATGATC
Blon-gAM39-10AC-spacer 58	BlongMCC10064ph1	100.000	1.09e-12	CTCTCGCTGGCAAACAACCAA-TAGAAAGGATGATC
Blon-gAM39-10AC-spacer 58	BlongMCC10044ph1	100.000	1.09e-12	CTCTCGCTGGCAAACAACCAA-TAGAAAGGATGATC
Blon-gAM39-10AC-spacer 58	BlongMCC10038ph1	100.000	1.09e-12	CTCTCGCTGGCAAACAACCAA-TAGAAAGGATGATC
Blon-gAM39-10AC-spacer 58	BlongLO-K29aph1	100.000	1.09e-12	CTCTCGCTGGCAAACAACCAA-TAGAAAGGATGATC
Blon-gAM39-10AC-spacer 66	Blongssp__3_mod ph1	100.000	1.09e-12	CGCGACGCGATGGCCGACGGGCAGATCAC-CGCGCA
Blon-gAM39-10AC-spacer 66	BlongMCC10129ph1	100.000	1.09e-12	CGCGACGCGATGGCCGACGGGCAGATCAC-CGCGCA
Blon-gAM39-8AC-spacer9	Bre017W439ph2	100.000	1.24e-11	GAC-CGCCCTCGCGGCGGCCCGCCAGCGATGTTT
Blon-gAM39-8AC-spacer9	Blong-CECT7210.2ph3	100.000	1.24e-11	GAC-CGCCCTCGCGGCGGCCCGCCAGCGATGTTT
Blon-gAM39-8AC-spacer9	Blong-CCUG30698ph3	100.000	1.24e-11	GAC-CGCCCTCGCGGCGGCCCGCCAGCGATGTTT
Blon-gAPC1461-spacer107	Blon-gAM39-8ACph1	100.000	3.69e-12	GCTCTATCCTCCACACCCTGTAGGCATCGA-TATG
Blon-gAPC1461-spacer111	BlongTF06-45Aph3	100.000	1.24e-11	CGCGTATTTCTGCACAGTGCCCGAAGCGTCCTT
Blon-gAPC1461-spacer111	BlongAF08-2ph1	100.000	1.24e-11	CGCGTATTTCTGCACAGTGCCCGAAGCGTCCTT
Blon-gAPC1461-spacer119	BreNRBB52ph4	100.000	3.69e-12	GGTCGGCCAGCGTCTGGTCGGTCGCGGTGAAG TC
Blon-gAPC1461-spacer121	BlongLO-K29bph1	100.000	3.69e-12	CCTCGCCCTCGCGCCCCGGCTGCAGTATGCG-GAT
Blon-gAPC1461-spacer123	BreDRBB28ph1	100.000	3.69e-12	GAACACCATCGCCGACACCGTGCG-CAAGGCCGTG
Blon-gAPC1461-spacer123	BreCNCMI-4321ph1	100.000	3.69e-12	GAACACCATCGCCGACACCGTGCG-CAAGGCCGTG
Blon-gAPC1461-spacer123	Bre689bph1	100.000	3.69e-12	GAACACCATCGCCGACACCGTGCG-CAAGGCCGTG
Blon-gAPC1461-spacer125	BreDRBB28ph1	100.000	3.21e-13	TTCATGCTCGATGCTGATGGTGCTGTTGTT-GCTCGA
Blon-gAPC1461-spacer125	BreCNCMI-4321ph1	100.000	3.21e-13	TTCATGCTCGATGCTGATGGTGCTGTTGTT-GCTCGA
Blon-gAPC1461-spacer125	Bre689bph1	100.000	3.21e-13	TTCATGCTCGATGCTGATGGTGCTGTTGTT-GCTCGA
Blon-gAPC1461-spacer28	BreNRBB01ph2	100.000	3.69e-12	GTGGCGGTCTGCGAGTCCGCCGTCCGCCAC-GTGA
Blon-	BreDSM20213.3ph2	100.000	3.69e-12	GTGGCGGTCTGCGAGTCCGCCGTCCGCCAC-

Spacer Name	Phage Name	Identity	E-value	Nucleotide Sequence
gAPC1461-spacer28				GTGA
Blon-gAPC1461-spacer28	BreDRBB30ph3	100.000	3.69e-12	GTGGCGGTCTGCGAGTCCGCCGTCCGCCAC-GTGA
Blon-gAPC1461-spacer28	BreCNCMI-4321ph3	100.000	3.69e-12	GTGGCGGTCTGCGAGTCCGCCGTCCGCCAC-GTGA
Blon-gAPC1461-spacer63	BreDRBB28ph1	100.000	1.24e-11	GGTCGCTTCATGGGTCATGCCCCGCTTGTGGGT
Blon-gAPC1461-spacer63	BreCNCMI-4321ph1	100.000	1.24e-11	GGTCGCTTCATGGGTCATGCCCCGCTTGTGGGT
Blon-gAPC1461-spacer63	Bre689bph1	100.000	1.24e-11	GGTCGCTTCATGGGTCATGCCCCGCTTGTGGGT
Blon-gAPC1461-spacer87	Bre689bph1	100.000	1.09e-12	ACGGTGGCTTCGCCTTCTTCGTCCAGCAC-CACGGT
Blon-gAPC1461-spacer93	BlongTF06-12ACph1	100.000	3.21e-13	TTTGCGCTTGAGACGTTTGAGGCTCATGGCG-TACAG
Blon-gAPC1461-spacer93	BlongTF07-39ph1	100.000	1.49e-11	TTTGCGCTTGAGACGTTTGAGGCTCATGGCGTA
Blon-gAPC1461-spacer93	BlongTF07-31ph1	100.000	1.49e-11	TTTGCGCTTGAGACGTTTGAGGCTCATGGCGTA
Blon-gAPC1473-spacer85	BreBR3ph3	98.630	1.79e-31	TGTTTTTGTTCAATAGGAAGTTGAGGTTTTAG-TCACGAAGATTTTTCGGGTTTAGGCG-GAAAGGCATGCCGAC
Blon-gAPC1473-spacer90	BlongAM16-2ph1	100.000	5.49e-09	TGGATGGCTTTATCCCGACGACGACCAC
Blon-gAPC1476-spacer115	BlongTM01-1ph1	100.000	5.49e-09	GACGCCAACGACCAGTTTCGCGATTTCG
Blon-gAPC1476-spacer115	BlongMCC10101ph1	100.000	5.49e-09	GACGCCAACGACCAGTTTCGCGATTTCG
Blon-gAPC1476-spacer115	BlongGT15ph3	100.000	5.49e-09	GACGCCAACGACCAGTTTCGCGATTTCG
Blon-gAPC1476-spacer115	BlongDS15_3ph1	100.000	5.49e-09	GACGCCAACGACCAGTTTCGCGATTTCG
Blon-gAPC1476-spacer115	BlongBB-79ph1	100.000	5.49e-09	GACGCCAACGACCAGTTTCGCGATTTCG
Blon-gAPC1476-spacer115	BlongAF30-12ph2	100.000	5.49e-09	GACGCCAACGACCAGTTTCGCGATTTCG
Blon-gAPC1476-spacer91	BlongN3A01ph1	100.000	5.49e-09	TTTCGTATGCGGGGTAGGCGTTGCGGTT
Blon-gAPC1477-spacer101	BlongE18ph4	100.000	3.69e-12	ACCCGGTGCCGAAAGGGCCGACCGTGTAC-GAGGC
Blon-gAPC1477-spacer35	BlongAM16-2ph1	100.000	3.21e-13	GATGAGGCGGCCGTTTTTCACCAGCAGCAC-CTTGTC
Blon-gAPC1477-spacer35	BlongAF30-12ph2	100.000	3.21e-13	GATGAGGCGGCCGTTTTTCACCAGCAGCAC-CTTGTC
Blon-gAPC1477-spacer45	BreNRBB50ph1	100.000	1.09e-12	GCCGAGCAGCGCAGGAACAAC-GCCGTGCCGTCCTC
Blon-gAPC1477-spacer67	BlongMCC10115ph1	100.000	1.22e-12	AAGGGCGATTT-GGCGGCCCGCGTGGTCGTCCACTG
Blon-gAPC1477-spacer93	BlongTM01-1ph1	100.000	1.09e-12	CGCATCGTGGCCACGAAC-CGTTTCTGGCCGACCAC
Blon-gAPC1477-spacer93	BlongDS15_3ph1	100.000	1.09e-12	CGCATCGTGGCCACGAAC-CGTTTCTGGCCGACCAC
Blon-gAPC1477-spacer93	BlongBB-79ph1	100.000	1.09e-12	CGCATCGTGGCCACGAAC-CGTTTCTGGCCGACCAC
Blon-gAPC1477-spacer93	BlongAF30-12ph2	100.000	1.09e-12	CGCATCGTGGCCACGAAC-CGTTTCTGGCCGACCAC

Spacer Name	Phage Name	Identity	E-value	Nucleotide Sequence
Blon-gAPC1477-spacer93	BlongMCC10101ph1	100.000	1.41e-11	CATCGTGGCCACGAACCGTTTCTGGCCGACCAC
Blon-gAPC1477-spacer93	BlongGT15ph3	100.000	1.41e-11	CATCGTGGCCACGAACCGTTTCTGGCCGACCAC
Blon-gAPC1482-spacer129	BlongAM16-2ph1	100.000	3.21e-13	GATGCGGTCGGCGCCGTAGTCCACGCCACGG-TACGA
Blon-gAPC1482-spacer57	Blong-CCUG30698ph3	100.000	1.09e-12	GTCACCAAGGTCAAGGCCCACGGCCCCTCG-CAACA
Blon-gATCC15697-spacer0	Blon-gATCC15697ph3	100.000	3.77e-48	GGCCGCTTTGGCGGTTAC-CGCCGACAAACTCGCCGCCAACTCGGTGGTGGCGGGCAAGCTCGCGGCCAACAGCGTGGAC-GCGGGCAACATCGTCGCCGGC
Blon-gATCC15697-spacer0	Blon-gATCC15697.2ph3	100.000	3.77e-48	GGCCGCTTTGGCGGTTAC-CGCCGACAAACTCGCCGCCAACTCGGTGGTGGCGGGCAAGCTCGCGGCCAACAGCGTGGAC-GCGGGCAACATCGTCGCCGGC
Blon-gATCC15697-spacer2	Blon-gATCC15697ph3	100.000	9.42e-14	CGCCAACTCGGTGGACGCGAGCAA-GATCGTGGCCGGA
Blon-gATCC15697-spacer2	Blon-gATCC15697.2ph3	100.000	9.42e-14	CGCCAACTCGGTGGACGCGAGCAA-GATCGTGGCCGGA
Blon-gATCC15697-spacer37	BlongJCM1217ph1	100.000	1.94e-09	GGCGGCCGACAGTACACAGACGGACTGTT
Blon-gATCC15697-spacer4	Blon-gATCC15697ph3	100.000	3.77e-48	CGCGTTGGCGGTGACCGCCGACAA-GCTCGCCGCGAACAGCGTAGTGGCGGGCAA-GATCGCCGCGAACGCGGTCAACGCGGG-CAACATCGTCTCGGGGGCC
Blon-gATCC15697-spacer4	Blon-gATCC15697.2ph3	100.000	3.77e-48	CGCGTTGGCGGTGACCGCCGACAA-GCTCGCCGCGAACAGCGTAGTGGCGGGCAA-GATCGCCGCGAACGCGGTCAACGCGGG-CAACATCGTCTCGGGGGCC
Blong-BBMN68-spacer52	BreNRBB56ph2	100.000	1.40e-27	GCTGGCCAA-GCGAGGGAACACAACAATGAAAACGCCAG-TTACACACTTAACGAGACAGGCCCG
Blong-BBMN68-spacer52	BreIDCC4401ph1	100.000	1.40e-27	GCTGGCCAA-GCGAGGGAACACAACAATGAAAACGCCAG-TTACACACTTAACGAGACAGGCCCG
Blong-BBMN68-spacer72	BlongE18ph4	100.000	4.47e-11	GTGATGGGCAGGTTGCCGCCGGTCCAGGTGAG
Blong-BBMN68-spacer72	Blon-gAF34-9ACph1	100.000	4.47e-11	GTGATGGGCAGGTTGCCGCCGGTCCAGGTGAG
BlongBG7-spacer62	BlongLO-K29bph1	100.000	1.67e-09	TGCAGTATGCGGATGAAGGTGTCCCTCAC
BlongBG7-spacer62	BlongAPC1462ph1	100.000	1.67e-09	TGCAGTATGCGGATGAAGGTGTCCCTCAC
BlongBG7-spacer62	Blong72Bph1	100.000	1.67e-09	TGCAGTATGCGGATGAAGGTGTCCCTCAC
Blong-Bi-26-spacer124	Brelw01ph1	100.000	4.47e-11	GGAAGGAGGGAACGTCCATCATGGCATCGTGT
Blong-Bi-26-spacer124	BlongNCTC11817ph3	100.000	4.47e-11	GGAAGGAGGGAACGTCCATCATGGCATCGTGT
Blong-Bi-26-spacer126	BlongTM01-1ph1	100.000	4.47e-11	GTCGCGCCGCCCGGCCTCAGCTGGCTGCCGTT
Blong-Bi-26-spacer126	BlongMCC10101ph1	100.000	4.47e-11	GTCGCGCCGCCCGGCCTCAGCTGGCTGCCGTT
Blong-Bi-26-spacer126	BlongGT15ph3	100.000	4.47e-11	GTCGCGCCGCCCGGCCTCAGCTGGCTGCCGTT

Spacer Name	Phage Name	Identity	E-value	Nucleotide Sequence
Blong-Bi-26-spacer126	BlongDS15_3ph1	100.000	4.47e-11	GTCGCGCCGCCCGGCCTCAGCTGGCTGCCGTT
Blong-Bi-26-spacer126	BlongBB-79ph1	100.000	4.47e-11	GTCGCGCCGCCCGGCCTCAGCTGGCTGCCGTT
Blong-Bi-26-spacer126	BlongAF30-12ph2	100.000	4.47e-11	GTCGCGCCGCCCGGCCTCAGCTGGCTGCCGTT
Blong-Bi-26-spacer140	BreDRBB28ph4	100.000	4.47e-11	GGCACCAGCAGCATGGCCACAA-GCCAGGCGAC
Blong-Bi-26-spacer140	BlongMCC10124ph1	100.000	4.47e-11	GGCACCAGCAGCATGGCCACAA-GCCAGGCGAC
Blong-Bi-26-spacer140	BlongMCC10102ph1	100.000	4.47e-11	GGCACCAGCAGCATGGCCACAA-GCCAGGCGAC
Blong-Bi-26-spacer140	BlongMCC10052ph1	100.000	4.47e-11	GGCACCAGCAGCATGGCCACAA-GCCAGGCGAC
Blong-Bi-26-spacer166	BadTF06-29ph2	100.000	4.47e-11	TTTTCCTTGATCAGGTGGTAGACCTGCGCGGC
Blong-Bi-26-spacer168	Bad1-11ph1	100.000	4.47e-11	GAACTGGAGGTGACCCAGCAGGTGCTCGGAGA
BlongBi-26-spacer60	BlongPC4ph1	100.000	4.47e-11	TCCTTCACCTCGTCGCGCAGCTTGTCGATCTG
BlongBi-26-spacer66	BlongBifido_04ph1	100.000	7.49e-09	TCGAGGTGCTGACGTGGGACGGCACCCG
BlongBi-26-spacer66	Bad487Bph3	100.000	7.49e-09	TCGAGGTGCTGACGTGGGACGGCACCCG
BlongBi-26-spacer74	BlongMCC10079ph1	100.000	4.47e-11	GGCATGACCGAAGCCGCGTACCGCACCGAGGT
BlongBi-26-spacer76	BlongMCC10079ph1	100.000	4.47e-11	GTTTCGCCGATCACATCGTTCGGGGACCCGTC
BlongBi-26-spacer82	BlongMCC10116ph1	100.000	4.47e-11	AGTCGAACTGCTCGAAGCCAAAGCTCCTCGCA
BlongBi-26-spacer82	BlongMCC10115ph1	100.000	4.47e-11	AGTCGAACTGCTCGAAGCCAAAGCTCCTCGCA
BlongBi-26-spacer82	BlongMCC10111ph1	100.000	4.47e-11	AGTCGAACTGCTCGAAGCCAAAGCTCCTCGCA
BlongBi-26-spacer82	BlongMCC10093ph1	100.000	4.47e-11	AGTCGAACTGCTCGAAGCCAAAGCTCCTCGCA
BlongBi-26-spacer82	BlongMCC10077ph1	100.000	4.47e-11	AGTCGAACTGCTCGAAGCCAAAGCTCCTCGCA
BlongBi-26-spacer82	BlongMCC10073ph1	100.000	4.47e-11	AGTCGAACTGCTCGAAGCCAAAGCTCCTCGCA
BlongBi-26-spacer82	BlongMCC10064ph1	100.000	4.47e-11	AGTCGAACTGCTCGAAGCCAAAGCTCCTCGCA
BlongBi-26-spacer82	BlongMCC10044ph1	100.000	4.47e-11	AGTCGAACTGCTCGAAGCCAAAGCTCCTCGCA
BlongBi-26-spacer82	BlongMCC10038ph1	100.000	4.47e-11	AGTCGAACTGCTCGAAGCCAAAGCTCCTCGCA
BlongBi-26-spacer82	BlongLO-K29aph1	100.000	4.47e-11	AGTCGAACTGCTCGAAGCCAAAGCTCCTCGCA
BlongBi-26-spacer88	BlongPC4ph1	100.000	4.47e-11	GGCCGCTCATGTCTACGACATTCGCAAAGGCG
BlongBi-26-spacer92	BreNRBB52ph5	100.000	4.47e-11	CGACCATAGTCAACCAATCATTTACCAAAAGT
BlongBi-26-spacer92	BreLMC520ph3	100.000	4.47e-11	CGACCATAGTCAACCAATCATTTACCAAAAGT
BlongBi-26-spacer92	BreJR01ph1	100.000	4.47e-11	CGACCATAGTCAACCAATCATTTACCAAAAGT
BlongBi-26-spacer92	BreDRBB28ph5	100.000	4.47e-11	CGACCATAGTCAACCAATCATTTACCAAAAGT
BlongBi-26-spacer92	BreDRBB26ph1	100.000	4.47e-11	CGACCATAGTCAACCAATCATTTACCAAAAGT
BlongBi-26-spacer92	BreBR3ph2	100.000	4.47e-11	CGACCATAGTCAACCAATCATTTACCAAAAGT
BlongBi-26-spacer92	Bre082W48ph1	100.000	4.47e-11	CGACCATAGTCAACCAATCATTTACCAAAAGT
BlongBi-26-spacer92	BlongLO-K29bph1	100.000	4.47e-11	CGACCATAGTCAACCAATCATTTACCAAAAGT
BlongBi-26-spacer92	Blong-CCUG30698ph3	100.000	4.47e-11	CGACCATAGTCAACCAATCATTTACCAAAAGT
BlongBi-26-spacer92	Blong72Bph1	100.000	4.47e-11	CGACCATAGTCAACCAATCATTTACCAAAAGT

Spacer Name	Phage Name	Identity	E-value	Nucleotide Sequence
BlongBi-26-spacer96	BreNRBB52ph5	100.000	2.08e-09	GTGAGCCCCCGCGACGGCGGGGCCTCCGA
BlongBifi-do_02-spacer61	BreNRBB52ph5	100.000	5.49e-09	CCGTGGACGCAACCCATACAACACCAAG
BlongBifi-do_02-spacer61	BreMCC1605ph1	100.000	5.49e-09	CCGTGGACGCAACCCATACAACACCAAG
BlongBifi-do_02-spacer61	BreJR01ph1	100.000	5.49e-09	CCGTGGACGCAACCCATACAACACCAAG
BlongBifi-do_02-spacer61	BreDRBB28ph5	100.000	5.49e-09	CCGTGGACGCAACCCATACAACACCAAG
BlongBifi-do_02-spacer61	BreDRBB26ph1	100.000	5.49e-09	CCGTGGACGCAACCCATACAACACCAAG
BlongBifi-do_02-spacer61	Bre082W48ph1	100.000	5.49e-09	CCGTGGACGCAACCCATACAACACCAAG
BlongBifi-do_02-spacer61	Bre017W439ph2	100.000	5.49e-09	CCGTGGACGCAACCCATACAACACCAAG
BlongBifi-do_02-spacer61	Blong-CCUG30698ph3	100.000	5.49e-09	CCGTGGACGCAACCCATACAACACCAAG
BlongBifi-do_02-spacer61	Blong157Fph1	100.000	5.49e-09	CCGTGGACGCAACCCATACAACACCAAG
BlongBifi-do_06-spacer102	Blongssp_infantis_6_USA001_1ph2	100.000	1.24e-11	GACGGTGAGCCACGTTTGGATAAC-GGACAACTA
BlongBifi-do_06-spacer102	Blon-gATCC15697ph2	100.000	1.24e-11	GACGGTGAGCCACGTTTGGATAAC-GGACAACTA
BlongBifi-do_06-spacer102	Blon-gATCC15697.2ph2	100.000	1.24e-11	GACGGTGAGCCACGTTTGGATAAC-GGACAACTA
Blongbk-spacer0	BlongBORIph1	98.864	8.22e-40	AACCGGAAAAA-TAAGGAAAATGCATGCAACTGCCTAAACCCGAAAAAAATACATGACTAGACACGAAACAC-CGACTTGACGAAATACA
Blongbk-spacer0	Blong105-Aph2	98.864	8.22e-40	AACCGGAAAAA-TAAGGAAAATGCATGCAACTGCCTAAACCCGAAAAAAATACATGACTAGACACGAAACAC-CGACTTGACGAAATACA
Blong-BLOI2-spacer50	BlongVKP-MAc-1636ph1	100.000	1.67e-09	TGGAGGTGACACCATCGCCCATGTCGTTC
Blong-BLOI2-spacer50	BlongMCC10130ph1	100.000	1.67e-09	TGGAGGTGACACCATCGCCCATGTCGTTC
Blong-BLOI2-spacer50	BlongMCC10081ph2	100.000	1.67e-09	TGGAGGTGACACCATCGCCCATGTCGTTC
Blong-BSM11-5-spacer102	Blong-CECT7210.2ph3	100.000	3.69e-12	CGCGTGGCCTGAGCTCGCGCAATCGTTTGAC-CGC
Blong-BSM11-5-spacer102	Blong157Fph1	100.000	3.69e-12	CGCGTGGCCTGAGCTCGCGCAATCGTTTGAC-CGC
Blong-BSM11-5-spacer110	BadZJ2ph1	100.000	4.47e-11	AGGAAAGTCGACCCGGAGGCGCGATTCAA-GAA
Blong-BSM11-5-spacer110	BadTF06-2ACph1	100.000	4.47e-11	AGGAAAGTCGACCCGGAGGCGCGATTCAA-GAA
Blong-BSM11-5-spacer110	BadTF06-10ACph2	100.000	4.47e-11	AGGAAAGTCGACCCGGAGGCGCGATTCAA-GAA
Blong-BSM11-5-spacer110	BadAM12-59ph1	100.000	4.47e-11	AGGAAAGTCGACCCGGAGGCGCGATTCAA-GAA
Blong-BSM11-5-spacer110	BadAM12-20ph1	100.000	4.47e-11	AGGAAAGTCGACCCGGAGGCGCGATTCAA-GAA
Blong-BSM11-5-spacer110	BadAL46-7ph1	100.000	4.47e-11	AGGAAAGTCGACCCGGAGGCGCGATTCAA-GAA
Blong-	BadAF28-4ACph1	100.000	4.47e-11	AGGAAAGTCGACCCGGAGGCGCGATTCAA-

Spacer Name	Phage Name	Identity	E-value	Nucleotide Sequence
BSM11-5-spacer110				GAA
Blong-BSM11-5-spacer110	Bad22Lph1	100.000	4.47e-11	AGGAAAGTCGACCCGGAGGCGCGATTCAA-GAA
Blong-BSM11-5-spacer114	BlongU-MA3015ph1	100.000	1.49e-11	CGCCAAGTACACGCCATCCATGAC-CATCCGCGA
Blong-BSM11-5-spacer116	BlongMCC10040ph2	100.000	1.09e-12	CTGATCGTATGCCGCTATGCGAAGGGCTCG-CAGAA
Blong-BSM11-5-spacer120	Brelw01ph1	100.000	2.36e-09	GTCATGTCCAACGCGCGGTAGGAGAGCAG
Blong-BSM11-5-spacer120	BlongNCTC11817ph3	100.000	2.36e-09	GTCATGTCCAACGCGCGGTAGGAGAGCAG
Blong-BSM11-5-spacer130	BlongTF06-12ACph1	100.000	1.09e-12	TTGCCCCCGCACTTGGGACAC-GGGCTAATCCTGTG
Blong-BSM11-5-spacer142	Brelw01ph1	100.000	9.42e-14	GGCAACCCCAGATAACACACGCAAGCAAAC-CGACACG
Blong-BSM11-5-spacer142	BlongNCTC11817ph3	100.000	9.42e-14	GGCAACCCCAGATAACACACGCAAGCAAAC-CGACACG
Blong-BSM11-5-spacer158	BlongF8ph1	100.000	3.69e-12	CGCCTCGCAATGCGCCGCGTTCGCGGGCGCG-GAA
Blong-BSM11-5-spacer168	BreNRBB52ph4	100.000	1.09e-12	GTCCCGTCGTCGAACAGGACCCG-CATCCACCCGGA
Blong-BSM11-5-spacer168	Blongssp_infantis_6_USA001_1ph2	100.000	1.09e-12	GTCCCGTCGTCGAACAGGACCCG-CATCCACCCGGA
Blong-BSM11-5-spacer168	Blon-gATCC15697ph2	100.000	1.09e-12	GTCCCGTCGTCGAACAGGACCCG-CATCCACCCGGA
Blong-BSM11-5-spacer168	Blon-gATCC15697.2ph2	100.000	1.09e-12	GTCCCGTCGTCGAACAGGACCCG-CATCCACCCGGA
Blong-BSM11-5-spacer98	BreJR01ph1	100.000	1.24e-11	ACCTCCCTCGCCCAAAGA-GATCATGAGGGATTC
Blong-BSM11-5-spacer98	Blong-CECT7210.2ph3	100.000	1.24e-11	ACCTCCCTCGCCCAAAGA-GATCATGAGGGATTC
Blong-BSM11-5-spacer98	Blong-CCUG30698ph3	100.000	1.24e-11	ACCTCCCTCGCCCAAAGA-GATCATGAGGGATTC
Blong-BSM11-5-spacer98	Blong72Bph1	100.000	1.24e-11	ACCTCCCTCGCCCAAAGA-GATCATGAGGGATTC
Blong-BSM11-5-spacer98	Blong157Fph1	100.000	1.24e-11	ACCTCCCTCGCCCAAAGA-GATCATGAGGGATTC
Blong-DJO10A-spacer79	BlongEK5ph1	100.000	5.49e-09	TGAAGTTGCCGGTGTACGCGCCGTTGGC
Blong-DPC6317-spacer47	BlongMCC10115ph1	100.000	3.21e-13	ATCAAGGTCGAGGCCCGGTTGCAGCG-CATCCGACCA
Blong-DPC6317-spacer57	BreUMB0089ph2	100.000	3.21e-13	CTG-CAACAGCTCAAGGAGGCCCAACTGTCCGAA-GCC
Blong-DPC6317-spacer57	BlongMCC10130ph1	100.000	3.21e-13	CTG-CAACAGCTCAAGGAGGCCCAACTGTCCGAA-GCC
Blong-DPC6317-spacer57	BlongMCC10081ph2	100.000	3.21e-13	CTG-CAACAGCTCAAGGAGGCCCAACTGTCCGAA-GCC
Blong-DPC6317-spacer57	BlongBifido_06ph1	100.000	3.21e-13	CTG-CAACAGCTCAAGGAGGCCCAACTGTCCGAA-GCC
Blong-DPC6317-spacer57	BifPRI1ph2	100.000	3.21e-13	CTG-CAACAGCTCAAGGAGGCCCAACTGTCCGAA-GCC

Spacer Name	Phage Name	Identity	E-value	Nucleotide Sequence
Blong-DPC6320-spacer25	BlongE18ph5	100.000	2.32e-30	GCCACGTCCGCCGGCCGGTGCGGAG-CAGGCCACGCAACTGTTGCCCGAC-GCTGCTGCCGATTTCACCG
Blong-DPC6320-spacer25	Blong-CCUG30698ph4	100.000	2.32e-30	GCCACGTCCGCCGGCCGGTGCGGAG-CAGGCCACGCAACTGTTGCCCGAC-GCTGCTGCCGATTTCACCG
Blong-DPC6323-spacer93	BreNRBB02ph1	100.000	5.49e-09	TGAAGCACGGTGCCGTTGCTCAAACCGC
Blong-DSM20211-spacer22	BlongU-MA3015ph1	100.000	3.21e-13	TTCCGACACCGCGCACTACTAC-CTGATCGTCGAATA
Blong-DSM20211-spacer22	BlongMCC10116ph1	100.000	3.21e-13	TTCCGACACCGCGCACTACTAC-CTGATCGTCGAATA
Blong-DSM20211-spacer22	BlongMCC10115ph1	100.000	3.21e-13	TTCCGACACCGCGCACTACTAC-CTGATCGTCGAATA
Blong-DSM20211-spacer22	BlongMCC10111ph1	100.000	3.21e-13	TTCCGACACCGCGCACTACTAC-CTGATCGTCGAATA
Blong-DSM20211-spacer22	BlongMCC10100ph1	100.000	3.21e-13	TTCCGACACCGCGCACTACTAC-CTGATCGTCGAATA
Blong-DSM20211-spacer22	BlongMCC10093ph1	100.000	3.21e-13	TTCCGACACCGCGCACTACTAC-CTGATCGTCGAATA
Blong-DSM20211-spacer22	BlongMCC10077ph1	100.000	3.21e-13	TTCCGACACCGCGCACTACTAC-CTGATCGTCGAATA
Blong-DSM20211-spacer22	BlongMCC10073ph1	100.000	3.21e-13	TTCCGACACCGCGCACTACTAC-CTGATCGTCGAATA
Blong-DSM20211-spacer22	BlongMCC10064ph1	100.000	3.21e-13	TTCCGACACCGCGCACTACTAC-CTGATCGTCGAATA
Blong-DSM20211-spacer22	BlongMCC10044ph1	100.000	3.21e-13	TTCCGACACCGCGCACTACTAC-CTGATCGTCGAATA
Blong-DSM20211-spacer22	BlongMCC10040ph2	100.000	3.21e-13	TTCCGACACCGCGCACTACTAC-CTGATCGTCGAATA
Blong-DSM20211-spacer22	BlongMCC10038ph1	100.000	3.21e-13	TTCCGACACCGCGCACTACTAC-CTGATCGTCGAATA
Blong-DSM20211-spacer22	BlongLO-K29aph1	100.000	3.21e-13	TTCCGACACCGCGCACTACTAC-CTGATCGTCGAATA
BlongI-CIS-505-spacer120	BlongJCM1217ph1	100.000	6.59e-16	TTGACTTCTCCCCACGGCTAAAGCCGGGG-GATTCTAAGCTTT
BlongI-CIS-505-spacer20	BreNRBB02ph1	100.000	5.49e-09	GTACGGATATATGGCGGTTGCCGCCACA
BlongI-CIS-505-spacer22	BreNRBB02ph1	100.000	5.49e-09	TGCTGCTGGCCCTGTAGGGTGTCGGACT
BlongI-CIS-505-spacer92	BlongJSRL02ph2	100.000	4.47e-11	AATCGTCGATATGTCGCAGGGCCGCCCCGGCG
BlongI-CIS-505-spacer92	BlongAF30-11ph1	100.000	4.47e-11	AATCGTCGATATGTCGCAGGGCCGCCCCGGCG
Blon-gIN-07-spacer119	BlongMCC10044ph1	100.000	5.07e-11	TTGTCCAACGTCTGCGCGGCACCGACCACGAG
Blon-gIN-07-spacer121	Blon-gATCC15697ph3	100.000	4.15e-12	GCACGGGCGCGACGGTCACGACGCC-TACATCGCG
Blon-gIN-07-spacer121	Blon-gATCC15697.2ph3	100.000	4.15e-12	GCACGGGCGCGACGGTCACGACGCC-TACATCGCG
Blon-gIN-07-spacer149	BreCNCMI-4321ph2	98.969	8.17e-45	AAGCGCATGTACAAGGCCGCGAAATCAC-GAGCTGGCACTTCGGAAACTAATGCCG-TAATGAAGACCATGAAC-CACATGTTTCCCGACGAGCTCACCT
Blon-gIN-07-spacer149	Blongssp__3_mod ph1	98.969	8.17e-45	AAGCGCATGTACAAGGCCGCGAAATCAC-GAGCTGGCACTTCGGAAACTAATGCCG-

Spacer Name	Phage Name	Identity	E-value	Nucleotide Sequence
Blon-gIN-07-spacer149	BlongMCC10129ph1	98.000	8.17e-45	TAATGAAGACCATGAAC-CACATGTTTCCCGACGAGCTCACCT AAGCGCATGTACAAGGCCGCGAAATCAC-GAGCTGGCACTTCGGAAACTAATGCCG-TAATGAAGACCATGAAC-CACATGTTTCCCGACGAGCTCACCTTTG
BlongIN-07-spacer17	BlongF8ph1	100.000	1.09e-12	CTTTCTTGGGTCGTCATTTGACCCCG-TATCGGCGG
BlongIN-07-spacer17	BlongBG7ph1	100.000	1.09e-12	CTTTCTTGGGTCGTCATTTGACCCCG-TATCGGCGG
BlongIN-07-spacer19	BlongF8ph1	100.000	3.69e-12	AACTTCAACAAAGCAAACAGGAAGCACTAC-CGTA
BlongIN-07-spacer19	BlongBG7ph1	100.000	3.69e-12	AACTTCAACAAAGCAAACAGGAAGCACTAC-CGTA
BlongIN-07-spacer21	BlongF8ph1	100.000	3.69e-12	CATTTCACGTGGTAGAACAGGAAC-GCGGTCAGAC
BlongIN-07-spacer23	BlongBG7ph1	100.000	1.09e-12	TCTGCGCGCTCGTGCTGACCATTTAAGGCTT-GCCC
BlongIN-07-spacer27	Brelw01ph1	100.000	1.09e-12	CATTGGTCAGATTGCCGTTTATGCG-GATTGTCTGC
BlongIN-07-spacer33	BreDRBB30ph3	100.000	1.09e-12	TCAAGCTTCGCCAACTCGTATCGGAC-GGCCTCCGA
BlongIN-07-spacer33	BreCNCMI-4321ph3	100.000	1.09e-12	TCAAGCTTCGCCAACTCGTATCGGAC-GGCCTCCGA
BlongIN-07-spacer39	Brelw01ph1	100.000	1.24e-11	GCCAGCCAACCTCATCCATTTATGCGGCTCCGG
BlongIN-07-spacer41	Brelw01ph1	100.000	3.69e-12	TACACGACCAAGGATGCGGCAGACCAACTC-TACG
BlongIN-07-spacer41	BlongTF07-39ph1	100.000	3.69e-12	TACACGACCAAGGATGCGGCAGACCAACTC-TACG
BlongIN-07-spacer41	BlongTF07-31ph1	100.000	3.69e-12	TACACGACCAAGGATGCGGCAGACCAACTC-TACG
BlongIN-07-spacer41	BlongTF06-12ACph1	100.000	3.69e-12	TACACGACCAAGGATGCGGCAGACCAACTC-TACG
BlongIN-07-spacer41	Blong-BIC1307292462ph1	100.000	3.69e-12	TACACGACCAAGGATGCGGCAGACCAACTC-TACG
BlongIN-07-spacer41	Blon-gATCC15697.2ph1	100.000	3.69e-12	TACACGACCAAGGATGCGGCAGACCAACTC-TACG
BlongIN-07-spacer45	BlongMCC10079ph1	100.000	1.09e-12	TCAAA-TACTGCGCGGCTCTGGGTCTGACCCCCGAA
BlongIN-07-spacer99	BadTF06-29ph2	100.000	3.69e-12	CGATACCGGCCAAGGTCACGCAGATCAC-CGGCAT
BlongIN-07-spacer99	Bad1-11ph1	100.000	3.69e-12	CGATACCGGCCAAGGTCACGCAGATCAC-CGGCAT
Blon-gIN-F29-spacer100	BreNRBB52ph4	100.000	1.24e-11	TCGTCGGTCATATTGTCCTCGATTCTGCAGTTG
Blon-gIN-F29-spacer134	BreUMB0089ph2	100.000	4.47e-11	GGCTGCACGTCGTTGATGGGGTTGGCGATCTG
Blon-gIN-F29-spacer134	BifPRI1ph2	100.000	4.47e-11	GGCTGCACGTCGTTGATGGGGTTGGCGATCTG
Blon-gIN-F29-spacer136	BreUMB0089ph2	100.000	1.24e-11	CGGCTTGTTCGACAGGTCGTTGTAGCTTCCACT
Blon-gIN-F29-spacer136	BifPRI1ph2	100.000	1.24e-11	CGGCTTGTTCGACAGGTCGTTGTAGCTTCCACT
Blon-gIN-F29-spacer138	BreUMB0089ph2	100.000	3.69e-12	CGATCTGGCAGTCGCAGTCGTCGTG-GAACAGGCT
Blon-	BlongAF08-2ph1	100.000	8.48e-09	CGTCGGGGTTGGTTTCGAATGCTGTTTT

Spacer Name	Phage Name	Identity	E-value	Nucleotide Sequence
gIN-F29-spacer14				
Blon-gIN-F29-spacer82	BreNRBB52ph4	100.000	3.69e-12	TGGCGTAGGCCTGCCACTGGCTT-GCGTCGCCTCT
Blon-gIN-F29-spacer96	Brelw01ph1	100.000	2.36e-09	CGCAGGGTCTCGGCGGTCGTGGAATACGC
Blon-gIN-F29-spacer96	BlongNCTC11817ph3	100.000	2.36e-09	CGCAGGGTCTCGGCGGTCGTGGAATACGC
Blon-gIN-F29-spacer98	BlongMCC10094ph1	100.000	3.21e-13	CATGTCCACGTAGTCGAG-GATGTCGTCGCCGTTGTC
BlongJih1-spacer90	BlongTF07-39ph1	100.000	5.49e-09	ACATATGCGATGGCCTTGCGTTCACGCT
BlongJih1-spacer90	BlongTF07-31ph1	100.000	5.49e-09	ACATATGCGATGGCCTTGCGTTCACGCT
BlongJih1-spacer90	BlongTF06-12ACph1	100.000	5.49e-09	ACATATGCGATGGCCTTGCGTTCACGCT
BlongJih1-spacer90	Blong-BIC1307292462ph1	100.000	5.49e-09	ACATATGCGATGGCCTTGCGTTCACGCT
BlongJih1-spacer90	Blon-gATCC15697.2ph1	100.000	5.49e-09	ACATATGCGATGGCCTTGCGTTCACGCT
BlongJih1-spacer90	BlongAH1206ph2	100.000	5.49e-09	ACATATGCGATGGCCTTGCGTTCACGCT
BlongJih1-spacer96	BreDRBB30ph3	100.000	1.67e-09	TCACCATCGAAAAAATCGATGATGAGATT
BlongJih1-spacer96	BreCNCMI-4321ph3	100.000	1.67e-09	TCACCATCGAAAAAATCGATGATGAGATT
BlongJSRL02-spacer140	BreNRBB02ph1	100.000	3.21e-13	GCCACCGTGCCACGGCCACCGGCCTTGAAC-GGTATG
BlongJSRL02-spacer142	BreNRBB02ph1	100.000	9.42e-14	CCATGAGCCGCGCAATCACGCCATT-GGAGCCGACCAC
BlongJSRL02-spacer7	BreCNCMI-4321ph2	100.000	1.67e-09	AAGACGGGCATCCAAGTTTTCAGCCACCC
BlongJSRL02-spacer7	Blongssp__3_modph1	100.000	1.67e-09	AAGACGGGCATCCAAGTTTTCAGCCACCC
BlongJSRL02-spacer9	BreCNCMI-4321ph2	100.000	3.77e-48	GAAAGGGCTCAACCACAAGGAGAACAAC-CATGTTCCTCAACCACACACCCCGTCAC-GTCCGATTCGTCGCGGCCCCGCCG-GAAGGCGGCATGGCTTCCGG
BlongJSRL02-spacer9	Blongssp__3_modph1	100.000	3.77e-48	GAAAGGGCTCAACCACAAGGAGAACAAC-CATGTTCCTCAACCACACACCCCGTCAC-GTCCGATTCGTCGCGGCCCCGCCG-GAAGGCGGCATGGCTTCCGG
BlongMC1-spacer20	Brelw01ph1	100.000	2.68e-10	GGGCACGCTCACCAAAGCACCATCATAGCCG
BlongMC1-spacer22	BlongNCTC11817ph3	100.000	1.47e-12	CTCGAACAGATGCGTCTGATCCGCAAGGGG-GACCA
BlongMC1-spacer26	BlongSu859ph1	100.000	1.82e-11	CGGCCGAAACCGCCATGCAAGGTCGAG-TTCATT
BlongMC1-spacer35	BlongTF06-12ACph1	100.000	1.74e-11	GTGTTCAC-GGATGGCGGGTTCCTGCGCCGCGAG
BlongMC1-spacer35	Brelw01ph1	100.000	6.26e-11	GTGTTCACGGATGGCGGGTTCCTGCGCCGCGA
BlongMC1-spacer48	Brelw01ph1	100.000	1.74e-11	CGTAGGCGATGCGGGTCATGTCGGCGGCGTTTG
BlongMC1-spacer64	BreNRBB52ph5	100.000	6.63e-11	TGCCAGATAACCCAGCACGCCCGAG-CACATTCC
BlongMC1-spacer64	BreMCC1605ph1	100.000	6.63e-11	TGCCAGATAACCCAGCACGCCCGAG-CACATTCC
BlongMC1-spacer64	BreJR01ph1	100.000	6.63e-11	TGCCAGATAACCCAGCACGCCCGAG-CACATTCC
BlongMC1-spacer64	BreDRBB28ph5	100.000	6.63e-11	TGCCAGATAACCCAGCACGCCCGAG-CACATTCC
BlongMC1-spacer64	BreDRBB26ph1	100.000	6.63e-11	TGCCAGATAACCCAGCACGCCCGAG-CACATTCC

Spacer Name	Phage Name	Identity	E-value	Nucleotide Sequence
BlongMC1-spacer64	Bre082W48ph1	100.000	6.63e-11	TGCCAGATAACCCAGCACGCCCGAG-CACATTCC
BlongMC1-spacer64	Bre017W439ph2	100.000	6.63e-11	TGCCAGATAACCCAGCACGCCCGAG-CACATTCC
BlongMC1-spacer64	Blong-CCUG30698ph3	100.000	6.63e-11	TGCCAGATAACCCAGCACGCCCGAG-CACATTCC
BlongMC1-spacer64	Blong157Fph1	100.000	6.63e-11	TGCCAGATAACCCAGCACGCCCGAG-CACATTCC
BlongMC1-spacer67	BlongNCTC11817ph3	100.000	6.86e-11	TGTTTTATTGGTTTTCGTTGTGTGTGGGGCAG
BlongMC1-spacer71	BlongU-MA3015ph1	100.000	1.66e-11	TCGGCGGCACCTCGGGTCAGGGCCCTGAC-GCCG
BlongMC1-spacer71	BlongMCC10100ph1	100.000	1.66e-11	TCGGCGGCACCTCGGGTCAGGGCCCTGAC-GCCG
BlongMC1-spacer71	BlongMCC10079ph1	100.000	1.66e-11	TCGGCGGCACCTCGGGTCAGGGCCCTGAC-GCCG
BlongMC2-spacer143	Brelw01ph1	100.000	1.24e-11	GAAGACCCGATTGCATGGGGACAGCAC-GGCGAA
BlongMC2-spacer32	BreUMB0089ph2	100.000	4.47e-11	GTATGGTTCTTCGAGTGGGCTTCGGCATACGC
BlongMC2-spacer32	BlongBifido_04ph1	100.000	4.47e-11	GTATGGTTCTTCGAGTGGGCTTCGGCATACGC
BlongMC2-spacer66	BadTF06-29ph2	100.000	4.47e-11	AATCCCAGCACATGCGAAGAAAGGAAGAAA-GA
BlongMC2-spacer66	Bad1-11ph1	100.000	4.47e-11	AATCCCAGCACATGCGAAGAAAGGAAGAAA-GA
BlongMC2-spacer75	BifPRI1ph1	100.000	6.45e-31	GGGCGCGTTCCAGTGATCGTT-GCAACATCTGATCGACCGGTAGGAGGATCG-GATGGGCAGGTGGGCGTT
BlongMC2-spacer97	BreNRBB52ph5	100.000	7.33e-10	GATGCGTTCGATGGTCTTGGATACGAGGCC
BlongMC2-spacer97	BreMCC1605ph1	100.000	7.33e-10	GATGCGTTCGATGGTCTTGGATACGAGGCC
BlongMC2-spacer97	BreLMC520ph3	100.000	7.33e-10	GATGCGTTCGATGGTCTTGGATACGAGGCC
BlongMC2-spacer97	BreJR01ph1	100.000	7.33e-10	GATGCGTTCGATGGTCTTGGATACGAGGCC
BlongMC2-spacer97	BreDRBB28ph5	100.000	7.33e-10	GATGCGTTCGATGGTCTTGGATACGAGGCC
BlongMC2-spacer97	BreDRBB26ph1	100.000	7.33e-10	GATGCGTTCGATGGTCTTGGATACGAGGCC
BlongMC2-spacer97	BreBR3ph2	100.000	7.33e-10	GATGCGTTCGATGGTCTTGGATACGAGGCC
BlongMC2-spacer97	Bre082W48ph1	100.000	7.33e-10	GATGCGTTCGATGGTCTTGGATACGAGGCC
BlongMC2-spacer97	Bre017W439ph2	100.000	7.33e-10	GATGCGTTCGATGGTCTTGGATACGAGGCC
BlongMC2-spacer97	BlongLO-K29bph1	100.000	7.33e-10	GATGCGTTCGATGGTCTTGGATACGAGGCC
BlongMC2-spacer97	Blong-CECT7210.2ph3	100.000	7.33e-10	GATGCGTTCGATGGTCTTGGATACGAGGCC
BlongMC2-spacer97	Blong-CCUG30698ph3	100.000	7.33e-10	GATGCGTTCGATGGTCTTGGATACGAGGCC
BlongMC2-spacer97	BlongAPC1462ph1	100.000	7.33e-10	GATGCGTTCGATGGTCTTGGATACGAGGCC
BlongMC2-spacer97	Blong72Bph1	100.000	7.33e-10	GATGCGTTCGATGGTCTTGGATACGAGGCC
BlongMC2-spacer97	Blong157Fph1	100.000	7.33e-10	GATGCGTTCGATGGTCTTGGATACGAGGCC
BlongMC-42-spacer10	BlongAF04-13ph1	100.000	4.47e-11	ACTTGGCGCGGGGTCTTTTGGGCGTCGGCTGT
BlongMC-42-spacer10	BifTF06-13ph1	100.000	4.47e-11	ACTTGGCGCGGGGTCTTTTGGGCGTCGGCTGT
BlongMC-42-spacer10	BifMGYG-HGUT-02396ph1	100.000	4.47e-11	ACTTGGCGCGGGGTCTTTTGGGCGTCGGCTGT
BlongMC-42-spacer10	BifAM18-11ph1	100.000	4.47e-11	ACTTGGCGCGGGGTCTTTTGGGCGTCGGCTGT
BlongMC-42-spacer226	BlongMCC10081ph1	100.000	4.47e-11	CGGGGTTGGCGAGCGTCTTGTTGAGGAACGTG
BlongMC-42-spacer226	Blong-CECT7210.2ph3	100.000	5.79e-10	GGGTTGGCGAGCGTCTTGTTGAGGAACGTG

Spacer Name	Phage Name	Identity	E-value	Nucleotide Sequence
BlongMC-42-spacer226	Blong-CCUG30698ph3	100.000	5.79e-10	GGGTTGGCGAGCGTCTTGTTGAGGAACGTG
BlongMC-42-spacer242	BlongMCC10130ph1	100.000	4.47e-11	TCGTTGTCTGAAAGAATCGAATCCAAACCCAA
BlongMC-42-spacer242	BlongMCC10081ph2	100.000	4.47e-11	TCGTTGTCTGAAAGAATCGAATCCAAACCCAA
BlongMC-42-spacer242	BlongBifido_06ph1	100.000	4.47e-11	TCGTTGTCTGAAAGAATCGAATCCAAACCCAA
BlongMC-42-spacer242	BifPRI1ph2	100.000	4.47e-11	TCGTTGTCTGAAAGAATCGAATCCAAACCCAA
BlongMC-42-spacer284	BlongTF01-22ph1	100.000	6.63e-11	GTTGCCGTCGGTGTCGGCCTTGACGCCGAAGTA
BlongMC-42-spacer50	BlongAPC1462ph1	100.000	4.47e-11	GCGTCGGCCTCGCCTTCGCGCCCCGGCTGCAG
BlongMC-42-spacer50	Blong72Bph1	100.000	4.47e-11	GCGTCGGCCTCGCCTTCGCGCCCCGGCTGCAG
BlongMC-42-spacer50	Blong-CCUG30698ph3	100.000	1.61e-10	GCGTCGGCCTCGCCTTCGCGCCCCGGCTGCA
BlongMC-42-spacer50	Blong157Fph1	100.000	1.61e-10	GCGTCGGCCTCGCCTTCGCGCCCCGGCTGCA
BlongMC-42-spacer72	BlongAM16-2ph1	100.000	4.47e-11	GCCGGCGCCGACATGAAGGAGCTGAAGGAGGT
BlongMC-42-spacer74	BlongMCC10130ph1	100.000	4.47e-11	GACGTGGCCTCCATGGGACAGAAGCTCGCCGA
BlongMC-42-spacer8	BlongAF04-13ph1	100.000	4.47e-11	GTGGCCGGCCATGGCACGCAGATGGACAAGTT
BlongMC-42-spacer80	Brelw01ph1	100.000	4.47e-11	GATACCCCAATGAGTGCGCCGATACCAAACCT
BlongMC-42-spacer88	BlongTM01-1ph1	100.000	4.47e-11	GAAGCCGCTGATGATGCTGGAGCCTGCGTCGT
BlongMC-42-spacer88	BlongMCC10101ph1	100.000	4.47e-11	GAAGCCGCTGATGATGCTGGAGCCTGCGTCGT
BlongMC-42-spacer88	BlongGT15ph3	100.000	4.47e-11	GAAGCCGCTGATGATGCTGGAGCCTGCGTCGT
BlongMC-42-spacer88	BlongEK5ph1	100.000	4.47e-11	GAAGCCGCTGATGATGCTGGAGCCTGCGTCGT
BlongMC-42-spacer88	BlongDS15_3ph1	100.000	4.47e-11	GAAGCCGCTGATGATGCTGGAGCCTGCGTCGT
BlongMC-42-spacer88	BlongBB-79ph1	100.000	4.47e-11	GAAGCCGCTGATGATGCTGGAGCCTGCGTCGT
BlongMC-42-spacer98	BlongU-MA3015ph1	100.000	4.47e-11	GGCTTCCGCAGCGCGGTCGACAAGGAGATGCG
BlongMC-42-spacer98	BlongMCC10116ph1	100.000	4.47e-11	GGCTTCCGCAGCGCGGTCGACAAGGAGATGCG
BlongMC-42-spacer98	BlongMCC10111ph1	100.000	4.47e-11	GGCTTCCGCAGCGCGGTCGACAAGGAGATGCG
BlongMC-42-spacer98	BlongMCC10100ph1	100.000	4.47e-11	GGCTTCCGCAGCGCGGTCGACAAGGAGATGCG
BlongMC-42-spacer98	BlongMCC10093ph1	100.000	4.47e-11	GGCTTCCGCAGCGCGGTCGACAAGGAGATGCG
BlongMC-42-spacer98	BlongMCC10040ph2	100.000	4.47e-11	GGCTTCCGCAGCGCGGTCGACAAGGAGATGCG
BlongMC-42-spacer98	BlongMCC10038ph1	100.000	4.47e-11	GGCTTCCGCAGCGCGGTCGACAAGGAGATGCG
BlongMC-42-spacer98	BlongLO-K29aph1	100.000	4.47e-11	GGCTTCCGCAGCGCGGTCGACAAGGAGATGCG
BlongMC-42-spacer9	BlongMCC10115ph	100.000	1.61e-10	GGCTTCCGCAGCGCGGTCGACAAGGAGATGC

Spacer Name	Phage Name	Identity	E-value	Nucleotide Sequence
8	1			
BlongMC-42-spacer98	BlongMCC10077ph1	100.000	1.61e-10	GGCTTCCGCAGCGCGGTCGACAAGGAGATGC
BlongMCC10006-spacer58	BlongTM01-1ph1	100.000	5.49e-09	TCCATCTCCTGTTCGAGCAGTCCTATCG
BlongMCC10006-spacer58	BlongMCC10101ph1	100.000	5.49e-09	TCCATCTCCTGTTCGAGCAGTCCTATCG
BlongMCC10006-spacer58	BlongGT15ph3	100.000	5.49e-09	TCCATCTCCTGTTCGAGCAGTCCTATCG
BlongMCC10006-spacer58	BlongDS15_3ph1	100.000	5.49e-09	TCCATCTCCTGTTCGAGCAGTCCTATCG
BlongMCC10006-spacer58	BlongBB-79ph1	100.000	5.49e-09	TCCATCTCCTGTTCGAGCAGTCCTATCG
BlongMCC10006-spacer58	BlongAF30-12ph2	100.000	5.49e-09	TCCATCTCCTGTTCGAGCAGTCCTATCG
BlongMCC10007-spacer103	Blong-CECT7210.2ph4	100.000	5.49e-09	GGCCTCAAGTTCGCGCGTCGTCTCGATG
BlongMCC10007-spacer109	Blong72Bph1	100.000	5.49e-09	CGAACATCGTCCGCAACTTCATTGATAA
BlongMCC10007-spacer109	Blong157Fph1	100.000	5.49e-09	CGAACATCGTCCGCAACTTCATTGATAA
BlongMCC10007-spacer129	Brelw01ph1	100.000	5.49e-09	GGAGGATTTTCATGTCGATATCCATAGT
BlongMCC10012-spacer22	Blong105-Aph1	100.000	5.49e-09	CTTGGACGACGAGACCGGCAAACTGATC
BlongMCC10012-spacer24	BlongAF04-13ph1	100.000	5.49e-09	GATGGACTGGTCAGCAGCGTCAGCATCG
BlongMCC10012-spacer24	Blong105-Aph1	100.000	5.49e-09	GATGGACTGGTCAGCAGCGTCAGCATCG
BlongMCC10023-spacer84	BreNRBB02ph1	100.000	5.49e-09	ACGCGGCGCTGATGACCGCGATAGCGCA
BlongMCC10033-spacer103	BreDRBB28ph1	100.000	5.49e-09	CCTCCCAGCGGATACCTGCACGTGCGGT
BlongMCC10033-spacer103	BreCNCMI-4321ph1	100.000	5.49e-09	CCTCCCAGCGGATACCTGCACGTGCGGT
BlongMCC10033-spacer103	Bre689bph1	100.000	5.49e-09	CCTCCCAGCGGATACCTGCACGTGCGGT
BlongMCC10033-spacer97	BreNRBB52ph1	100.000	5.49e-09	GTTGCGGTCGCCGGCCTTGTACAGGTCG
BlongMCC10033-spacer97	BreNRBB50ph1	100.000	5.49e-09	GTTGCGGTCGCCGGCCTTGTACAGGTCG
BlongMCC10041-spacer82	BlongMCC10079ph1	100.000	5.49e-09	AGTGGTCGGCTCCGGATAAATGCGAGCT
BlongMCC10041-spacer82	BlongMCC10077ph1	100.000	5.49e-09	AGTGGTCGGCTCCGGATAAATGCGAGCT
BlongMCC10041-spacer82	BlongMCC10064ph1	100.000	5.49e-09	AGTGGTCGGCTCCGGATAAATGCGAGCT
BlongMCC10041-spacer82	BlongMCC10044ph1	100.000	5.49e-09	AGTGGTCGGCTCCGGATAAATGCGAGCT
BlongMCC10041-spacer82	BlongMCC10038ph1	100.000	5.49e-09	AGTGGTCGGCTCCGGATAAATGCGAGCT
BlongMCC10041-spacer82	BlongLO-K29aph1	100.000	5.49e-09	AGTGGTCGGCTCCGGATAAATGCGAGCT
BlongMCC10060-spacer132	BlongMCC10094ph1	100.000	3.77e-48	CCGATGTCTATTTAGAAGTGCAACACCCTT-GTTGGTCCTGTAATTCTAACAG-TTCGTCGGCGAACGCCTCGGCGGGCGTGCGG-

Spacer Name	Phage Name	Identity	E-value	Nucleotide Sequence
				TAGCCGAGCACGCGCAT
BlongMCC10070-spacer218	BlongSu859ph1	100.000	5.49e-09	TGTTCTTGCGCGGCCTTCCCCGCCGACA
BlongMCC10070-spacer35	Blong-CECT7210.2ph4	100.000	1.24e-11	CCATTGGGTTATCGTGTGGCCGGAGATGCACGC
BlongMCC10071-spacer126	BlongF8ph1	100.000	5.49e-09	ACGCAGAAACCATAGACGTCGTGCCAAC
BlongMCC10071-spacer126	BlongBG7ph1	100.000	5.49e-09	ACGCAGAAACCATAGACGTCGTGCCAAC
BlongMCC10074-spacer109	Blong157Fph1	100.000	5.49e-09	TTCTACCTGTTCAATGCTTTCGATGTTG
BlongMCC10086-spacer133	BlongTM01-1ph1	100.000	1.67e-09	ATCCCCGACCGTACCGCGTTCTTTGATTT
BlongMCC10086-spacer133	BlongMCC10101ph1	100.000	1.67e-09	ATCCCCGACCGTACCGCGTTCTTTGATTT
BlongMCC10086-spacer133	BlongGT15ph3	100.000	1.67e-09	ATCCCCGACCGTACCGCGTTCTTTGATTT
BlongMCC10086-spacer133	BlongDS15_3ph1	100.000	1.67e-09	ATCCCCGACCGTACCGCGTTCTTTGATTT
BlongMCC10086-spacer133	BlongBB-79ph1	100.000	1.67e-09	ATCCCCGACCGTACCGCGTTCTTTGATTT
BlongMCC10086-spacer133	BlongAF30-12ph2	100.000	1.67e-09	ATCCCCGACCGTACCGCGTTCTTTGATTT
BlongMCC10086-spacer139	BreNRBB02ph1	100.000	5.49e-09	AGACCTGATTATCCCCGTCTTGGAGCGC
BlongMCC10096-spacer77	BlongAF08-2ph2	100.000	5.49e-09	TCTATGAGGATCTGCTACGCCCCATCAA
BlongMCC10097-spacer36	BlongBifido_03ph1	100.000	5.49e-09	CACATACGACTATTCCAACGCGGATTTG
BlongMCC10097-spacer54	BlongU-MA3015ph1	100.000	5.49e-09	TTCCAATTCGACAGGATGCTTCCAATCC
BlongMCC10097-spacer54	BlongMCC10100ph1	100.000	5.49e-09	TTCCAATTCGACAGGATGCTTCCAATCC
BlongMCC10097-spacer64	Blong-CCUG30698ph3	100.000	5.49e-09	GGTCTGCTGAGGCTGCTGTGGCGCGTAC
BlongMCC10097-spacer70	BreNRBB01ph2	100.000	1.67e-09	GGCTATTCCGGCTCCATGCCGTCCGAGGT
BlongMCC10097-spacer70	BreDSM20213.3ph2	100.000	1.67e-09	GGCTATTCCGGCTCCATGCCGTCCGAGGT
BlongMCC10106-spacer110	BlongAM16-2ph1	100.000	5.49e-09	GTGGTCGCGCACATACGCGGTCTTGCCC
BlongMCC10106-spacer120	BlongMCC10081ph1	100.000	5.49e-09	GACGTGGTCGATGAGCACGACCGCTGGC
BlongMCC10106-spacer98	BreNRBB01ph2	100.000	5.49e-09	CCTTCGCTCCACTTGTCAGGCACGCCAT
BlongMCC10106-spacer98	BreDSM20213.3ph2	100.000	5.49e-09	CCTTCGCTCCACTTGTCAGGCACGCCAT
BlongMCC10120-spacer76	BlongTM01-1ph1	100.000	1.67e-09	CCCCGCAATCGTTCGTAAGCGCGTTGGAG
BlongMCC10120-spacer76	BlongMCC10101ph1	100.000	1.67e-09	CCCCGCAATCGTTCGTAAGCGCGTTGGAG
BlongMCC10120-spacer76	BlongGT15ph3	100.000	1.67e-09	CCCCGCAATCGTTCGTAAGCGCGTTGGAG
BlongMCC10120-spacer76	BlongDS15_3ph1	100.000	1.67e-09	CCCCGCAATCGTTCGTAAGCGCGTTGGAG
BlongMCC10120-spa	BlongBB-79ph1	100.000	1.67e-09	CCCCGCAATCGTTCGTAAGCGCGTTGGAG

Spacer Name	Phage Name	Identity	E-value	Nucleotide Sequence
cer76				
BlongMCC10120-spacer76	BlongAF30-12ph2	100.000	1.67e-09	CCCCGCAATCGTTCGTAAGCGCGTTGGAG
BlongMCC10126-spacer19	BreCNCMI-4321ph2	99.000	1.76e-46	GAAAGGGCTCAACCACAAGGAGAACAAC-CATGTTCCTCAAC-CACACACCCCGTCATGTCCGAT-TCGTCGCGGCCCCGCCGGAAGGCGG-CATGGCTTCCGG
BlongMCC10126-spacer19	Blongssp__3_modph1	99.000	1.76e-46	GAAAGGGCTCAACCACAAGGAGAACAAC-CATGTTCCTCAAC-CACACACCCCGTCATGTCCGAT-TCGTCGCGGCCCCGCCGGAAGGCGG-CATGGCTTCCGG
BlongMCC10127-spacer26	BadP2P3ph1	100.000	6.95e-10	TGTTCGCGCAGAACGGCACCATCGGCGCCG
BlongN3A01-spacer158	BreLMC520ph3	100.000	3.69e-12	TGGCGGTCGAATCTCAGGTGCTGGCCTACAG-TCA
BlongN3A01-spacer158	BreBR3ph2	100.000	3.69e-12	TGGCGGTCGAATCTCAGGTGCTGGCCTACAG-TCA
BlongN3A01-spacer158	Blong-CECT7210.2ph3	100.000	3.69e-12	TGGCGGTCGAATCTCAGGTGCTGGCCTACAG-TCA
BlongN3A01-spacer158	Blong-CCUG30698ph3	100.000	3.69e-12	TGGCGGTCGAATCTCAGGTGCTGGCCTACAG-TCA
BlongN3A01-spacer158	BlongAPC1462ph1	100.000	3.69e-12	TGGCGGTCGAATCTCAGGTGCTGGCCTACAG-TCA
BlongN3A01-spacer158	Blong72Bph1	100.000	3.69e-12	TGGCGGTCGAATCTCAGGTGCTGGCCTACAG-TCA
BlongN3A01-spacer158	Blong157Fph1	100.000	3.69e-12	TGGCGGTCGAATCTCAGGTGCTGGCCTACAG-TCA
BlongN3A01-spacer160	BreLMC520ph3	100.000	3.69e-12	CATGCCCCTCACCCCCGAAATCGCCGCCCAATGC
BlongN3A01-spacer160	BreBR3ph2	100.000	3.69e-12	CATGCCCCTCACCCCCGAAATCGCCGCCCAATGC
BlongN3A01-spacer160	BlongAPC1462ph1	100.000	3.69e-12	CATGCCCCTCACCCCCGAAATCGCCGCCCAATGC
BlongN3A01-spacer160	Blong72Bph1	100.000	3.69e-12	CATGCCCCTCACCCCCGAAATCGCCGCCCAATGC
BlongN3A01-spacer160	Blong157Fph1	100.000	3.69e-12	CATGCCCCTCACCCCCGAAATCGCCGCCCAATGC
BlongN3A01-spacer24	BlongMCC10081ph1	100.000	3.69e-12	GGCAAGTAAAAACGCCTCCTCCAGCTGGCAG-TTC
BlongN3A01-spacer24	Blong-CECT7210.2ph3	100.000	3.69e-12	GGCAAGTAAAAACGCCTCCTCCAGCTGGCAG-TTC
BlongN3A01-spacer24	Blong-CCUG30698ph3	100.000	3.69e-12	GGCAAGTAAAAACGCCTCCTCCAGCTGGCAG-TTC
BlongN3A01-spacer28	BlongVKP-MAc-1636ph1	100.000	3.69e-12	CGCCACCCGCGCCGCGATCAGCCCGGCCAG-CATG
BlongN3A01-spacer28	BlongBifido_06ph1	100.000	3.69e-12	CGCCACCCGCGCCGCGATCAGCCCGGCCAG-CATG
BlongN3A01-spacer32	BlongMCC10040ph2	100.000	3.69e-12	AATCCCACACGTCGCTCATCGGCTTCAACAC-GTT
BlongN3A01-spacer34	BreNRBB52ph5	100.000	1.72e-10	GGAGGCTGCGGCAAACACAAGGGGCTCTGCG
BlongN3A01-spacer34	BreMCC1605ph1	100.000	1.72e-10	GGAGGCTGCGGCAAACACAAGGGGCTCTGCG
BlongN3A01-spacer3	BreDRBB28ph5	100.000	1.72e-10	GGAGGCTGCGGCAAACACAAGGGGCTCTGCG

Spacer Name	Phage Name	Identity	E-value	Nucleotide Sequence
4				
BlongN3A01-spacer34	BreDRBB26ph1	100.000	1.72e-10	GGAGGCTGCGGCAAACACAAGGGGCTCTGCG
BlongN3A01-spacer34	Bre017W439ph2	100.000	1.72e-10	GGAGGCTGCGGCAAACACAAGGGGCTCTGCG
BlongN3A01-spacer4	BlongMCC10115ph1	100.000	3.21e-13	ACGCCGCCCGAGACCTTGACGATTTTCTT-GGTTATC
BlongN3A01-spacer4	BlongMCC10111ph1	100.000	6.95e-10	ACGCCGCCCGAGACCTTGACGATTTTCTTG
BlongN3A01-spacer4	BlongMCC10040ph2	100.000	6.95e-10	ACGCCGCCCGAGACCTTGACGATTTTCTTG
BlongN3A01-spacer41	BlongMCC10077ph1	100.000	3.69e-12	CGAAGGAGCCGTGGATGGGAT-TTCTGAACAATCT
BlongN3A01-spacer41	BlongMCC10064ph1	100.000	3.69e-12	CGAAGGAGCCGTGGATGGGAT-TTCTGAACAATCT
BlongN3A01-spacer41	BlongMCC10044ph1	100.000	3.69e-12	CGAAGGAGCCGTGGATGGGAT-TTCTGAACAATCT
BlongN3A01-spacer41	BlongMCC10038ph1	100.000	3.69e-12	CGAAGGAGCCGTGGATGGGAT-TTCTGAACAATCT
BlongN3A01-spacer41	BlongLO-K29aph1	100.000	3.69e-12	CGAAGGAGCCGTGGATGGGAT-TTCTGAACAATCT
BlongN3A01-spacer43	BlongMCC10079ph1	100.000	1.43e-12	CGTAGGCTGTGCGAATCTTGTCCTCGTT-GGCCTTGC
BlongN3A01-spacer50	BlongTM01-1ph1	100.000	1.09e-12	TTTGATTTCGGCAT-ACCGTTCGATGCCGACCCGGA
BlongN3A01-spacer50	BlongMCC10101ph1	100.000	1.09e-12	TTTGATTTCGGCAT-ACCGTTCGATGCCGACCCGGA
BlongN3A01-spacer50	BlongGT15ph3	100.000	1.09e-12	TTTGATTTCGGCAT-ACCGTTCGATGCCGACCCGGA
BlongN3A01-spacer50	BlongDS15_3ph1	100.000	1.09e-12	TTTGATTTCGGCAT-ACCGTTCGATGCCGACCCGGA
BlongN3A01-spacer50	BlongBB-79ph1	100.000	1.09e-12	TTTGATTTCGGCAT-ACCGTTCGATGCCGACCCGGA
BlongN3A01-spacer50	BlongAF30-12ph2	100.000	1.09e-12	TTTGATTTCGGCAT-ACCGTTCGATGCCGACCCGGA
BlongN3A01-spacer54	BlongTM01-1ph1	100.000	4.47e-11	AAGCAGGGCATCCTGCCCGTCAAGTTCGCCGC
BlongN3A01-spacer54	BlongMCC10101ph1	100.000	4.47e-11	AAGCAGGGCATCCTGCCCGTCAAGTTCGCCGC
BlongN3A01-spacer54	BlongGT15ph3	100.000	4.47e-11	AAGCAGGGCATCCTGCCCGTCAAGTTCGCCGC
BlongN3A01-spacer54	BlongDS15_3ph1	100.000	4.47e-11	AAGCAGGGCATCCTGCCCGTCAAGTTCGCCGC
BlongN3A01-spacer54	BlongBB-79ph1	100.000	4.47e-11	AAGCAGGGCATCCTGCCCGTCAAGTTCGCCGC
BlongN3A01-spacer54	BlongAF30-12ph2	100.000	4.47e-11	AAGCAGGGCATCCTGCCCGTCAAGTTCGCCGC
BlongN3A01-spacer6	BlongVKP-MAc-1636ph1	100.000	1.09e-12	CCGGCCGCTGATTCGGCCATGTCGCCGGCAC-CACG
BlongN3A01-spacer6	BlongBifido_06ph1	100.000	1.09e-12	CCGGCCGCTGATTCGGCCATGTCGCCGGCAC-CACG
BlongN3A01-spacer72	BlongNCTC11817ph3	100.000	3.69e-12	ACTGCCCGAATGATTCGGGTG-GAGGTTTCATCGA
BlongN3A01-spacer79	BlongAF08-2ph2	100.000	2.76e-14	GGCCTCATCTCGTGAAGAACGTACACTGAG-TCATTCTG
BlongN3A01-spacer8	Brelw01ph1	100.000	6.17e-10	TATTGGCATGGTTCGGTGGTGATGAACATT

Spacer Name	Phage Name	Identity	E-value	Nucleotide Sequence
BlongN3A01-spacer81	BlongAF08-2ph2	100.000	3.77e-48	CAGCTGATTTCGTGAAGAACGTACGCTGAA-GATGCCCCCGGTGTCAC-TGATGACTGTTTTCGGTATGGAAGGCGGCG-CAGCAAGGTGTCCTAGGCTAATA
BlongN3A01-spacer98	BreUMB0089ph2	100.000	3.92e-12	GGTACACCCAGCCTCGCGAGCAGAG-CATCGTGCA
Blongssp_3-spacer23	BlongMCC10079ph1	100.000	1.09e-12	GTTGATTCTGGCCGTCATCCGGAATT-GTCGCCGGC
Blongssp_3-spacer25	Blong157Fph1	100.000	2.22e-09	ACGTTGCCGCCCGCGAAGCAGATATCACC
Blongssp_3-spacer27	BlongMCC10079ph1	100.000	3.21e-13	CTTCACTCGGTCACGGAAACGGCCGGTT-GCGGCTCC
Blongssp_3-spacer29	BreSC95ph1	100.000	6.17e-10	ATGGCGGCGGTGTACTTGCTGCGGTCGGTC
Blongssp_3-spacer31	BlongVKP-MAc-1636ph1	100.000	1.24e-11	TTCGAGGAGAACGTCAATGTCACGCAAC-CGAAA
Blongssp_3-spacer31	BlongMCC10130ph1	100.000	1.24e-11	TTCGAGGAGAACGTCAATGTCACGCAAC-CGAAA
Blongssp_3-spacer31	BlongBifido_06ph1	100.000	1.24e-11	TTCGAGGAGAACGTCAATGTCACGCAAC-CGAAA
Blongssp_3-spacer35	Blon-gATCC15697.2ph1	100.000	1.09e-12	AGGCATGGGTAAAGCGCGA-TAAGGCGGCTCGAATC
Blongssp_3-spacer39	BlongBifido_06ph1	100.000	3.69e-12	CTCGACCCGTTCCGAGTTCGGCGAC-CGCCAGATG
Blongssp_3-spacer41	BlongMCC10130ph1	100.000	3.69e-12	TGTCCCATGGAGGCCACGTCCTT-GGCGAGGTCCT
Blongssp_3-spacer41	BreUMB0089ph2	100.000	1.72e-10	CCCATGGAGGCCACGTCCTTGGCGAGGTCCT
Blongssp_3-spacer47	BreUMB0089ph2	100.000	3.69e-12	AGGGCTGAGCTGTTGCAGCGTCTCCCACAG-TTTC
Blongssp_3-spacer47	BlongMCC10130ph1	100.000	3.69e-12	AGGGCTGAGCTGTTGCAGCGTCTCCCACAG-TTTC
Blongssp_3-spacer47	BlongMCC10081ph2	100.000	3.69e-12	AGGGCTGAGCTGTTGCAGCGTCTCCCACAG-TTTC
Blongssp_3-spacer49	Blon-gATCC15697.2ph1	100.000	3.69e-12	AGTTATTGCCGTACGGCATGGCGTGAA-GCCGCCG
Blongssp_3-spacer55	Blon-gAM39-8ACph1	100.000	3.21e-13	CATGTACGGCGAGTTGGATGGTTT-GGAGGCGGCGAG
Blongssp_3-spacer61	BlongF8ph1	100.000	1.09e-12	TACGCGGCACCACGCATATGGTTCCTCG-GAGCCAA
Blongssp_3-spacer61	BifBIO5480ph1	100.000	1.09e-12	TACGCGGCACCACGCATATGGTTCCTCG-GAGCCAA
Blongssp_3-spacer67	BlongBifido_06ph1	100.000	1.09e-12	GAGCTGGACTCG-GAGGTCGCCGTCCTTCGCGGTGG
Blongssp_3-spacer69	BreSC95ph1	100.000	3.69e-12	ACCAAAAACGCCCCCACGCCCGCCAC-CGAAAACA
Blongssp_3-spacer72	BreBR3ph3	100.000	1.77e-41	TAAGGCCCACGGGTGGTCGG-CATGCCTTTCCGCCTAAACCCGAAAAAC-CTTCGTGACTAAAACCTCAACTTCC-TATTGAACAAAAACA
Blongssp_5-spacer121	BreMCC1605ph1	100.000	4.47e-11	TTGAACGCCAGCAGCTCGCAGACGGCACGGAA
Blongssp_5-spacer121	BreLMC520ph3	100.000	4.47e-11	TTGAACGCCAGCAGCTCGCAGACGGCACGGAA
Blongssp_5-spacer121	BreDRBB28ph5	100.000	4.47e-11	TTGAACGCCAGCAGCTCGCAGACGGCACGGAA
Blongssp_5-spacer121	BreDRBB26ph1	100.000	4.47e-11	TTGAACGCCAGCAGCTCGCAGACGGCACGGAA
Blongssp_5-spacer12	BreBR3ph2	100.000	4.47e-11	TTGAACGCCAGCAGCTCGCAGACGGCACGGAA

Spacer Name	Phage Name	Identity	E-value	Nucleotide Sequence
1				
Blongssp_5-spacer121	Bre082W48ph1	100.000	4.47e-11	TTGAACGCCAGCAGCTCGCAGACGGCACGGAA
Blongssp_5-spacer125	Blong157Fph1	100.000	4.47e-11	GTTGACGTGTCATTCGCAGTCATTTGGTGTGC
Blongssp_5-spacer27	Blong157Fph1	100.000	4.47e-11	GGTGTCGCCCCGAATACAGACCACATGCCCAT
Blongssp_5-spacer61	BifAM12-10ph1	100.000	4.47e-11	AACGCGCTTCCCGACAACCCGATGAGCATGTA
Blongssp_7-spacer162	Blong-CECT7210.2ph3	100.000	2.08e-09	ATGCAGTTGCGTTCCACCCGTTCGCCCTC
Blongssp_7-spacer166	BreSC95ph1	100.000	5.79e-10	CGCGGAAGCTGGTCGGTGGAATCCACCATC
Blongssp_7-spacer170	BlongTF01-22ph1	100.000	4.47e-11	TAGGCTTCGGCGATGTCCTGACGGCATTCGAT
Blongssp_7-spacer340	BreNRBB52ph5	100.000	5.49e-09	ATGGGCGGCCGCACGCATGCGTGGACGA
Blongssp_7-spacer340	BreMCC1605ph1	100.000	5.49e-09	ATGGGCGGCCGCACGCATGCGTGGACGA
Blongssp_7-spacer340	BreLMC520ph3	100.000	5.49e-09	ATGGGCGGCCGCACGCATGCGTGGACGA
Blongssp_7-spacer340	BreDRBB28ph5	100.000	5.49e-09	ATGGGCGGCCGCACGCATGCGTGGACGA
Blongssp_7-spacer340	BreDRBB26ph1	100.000	5.49e-09	ATGGGCGGCCGCACGCATGCGTGGACGA
Blongssp_7-spacer340	BreBR3ph2	100.000	5.49e-09	ATGGGCGGCCGCACGCATGCGTGGACGA
Blongssp_7-spacer340	Bre082W48ph1	100.000	5.49e-09	ATGGGCGGCCGCACGCATGCGTGGACGA
Blongssp_7-spacer340	Bre017W439ph2	100.000	5.49e-09	ATGGGCGGCCGCACGCATGCGTGGACGA
Blongssp_7-spacer340	BlongLO-K29bph1	100.000	5.49e-09	ATGGGCGGCCGCACGCATGCGTGGACGA
Blongssp_7-spacer340	Blong-CECT7210.2ph3	100.000	5.49e-09	ATGGGCGGCCGCACGCATGCGTGGACGA
Blongssp_7-spacer344	BlongE18ph4	100.000	5.49e-09	TTCGGACTCGGCGAGGCGGCGACAGTCG
Blongssp_7-spacer344	BlongBB-79ph2	100.000	5.49e-09	TTCGGACTCGGCGAGGCGGCGACAGTCG
Blongssp_7-spacer344	Blon-gAF34-9ACph1	100.000	5.49e-09	TTCGGACTCGGCGAGGCGGCGACAGTCG
Blongssp_7-spacer44	Blong-CECT7210.2ph3	100.000	4.47e-11	GAGTTTCAAGGATGACCGACATCTACGGATAC
Blongssp_7-spacer78	BlongEK5ph1	100.000	1.61e-10	TCGCAGGCGTCATCACCGCCGTATCCTCCGC
Blongssp_7-spacer80	BlongEK5ph1	100.000	4.47e-11	TTGCCGGTGTACGCGCCGTTGGCGAGCATGGC
Blongssp_7-spacer82	BlongEK5ph1	100.000	4.47e-11	AATCCGATGATGTTGTTCACGCACGAGCCGAT
Blongssp_7-spacer84	BlongEK5ph1	100.000	4.47e-11	TCGTCGCCGATGGTGCCTTCCATCGTCCATGT
Blongssp-spacer10	Brelw01ph1	100.000	1.09e-12	CACTCAGACTGATCGCACTGGTGTT-GAGCGAGGCG
Blongssp-spacer10	BlongNCTC11817ph3	100.000	1.09e-12	CACTCAGACTGATCGCACTGGTGTT-GAGCGAGGCG
Blongssp-spacer116	Blong-CCUG30698ph3	100.000	1.72e-10	TGGTATGACGATGCCCGTGTTCGCGTTCGGG
Blongssp-spacer12	BreUMB0089ph2	100.000	1.09e-12	ACGCAGACGCTTCCCAATGCGTTCCAG-TCCGTCGT
Blongssp-spacer12	BlongBifido_04ph1	100.000	1.09e-12	ACGCAGACGCTTCCCAATGCGTTCCAG-TCCGTCGT
Blongssp-spacer130	BlongPC4ph1	100.000	1.24e-11	CGGACAATATCGAACAGGCGTATTAC-

Spacer Name	Phage Name	Identity	E-value	Nucleotide Sequence
				GGATACA
Blongssp-spacer142	BreNRBB01ph2	100.000	3.69e-12	TTGCACTGCCGGAACCGTTCAATCGTG-TAGCGTT
Blongssp-spacer142	BreDSM20213.3ph2	100.000	3.69e-12	TTGCACTGCCGGAACCGTTCAATCGTG-TAGCGTT
Blongssp-spacer154	BreNRBB01ph2	100.000	4.77e-11	TCGGCCAACTCCGCAAGGAAGGACTATTGCCG
Blongssp-spacer154	BreDSM20213.3ph2	100.000	4.77e-11	TCGGCCAACTCCGCAAGGAAGGACTATTGCCG
Blongssp-spacer154	BreDRBB30ph3	100.000	4.77e-11	TCGGCCAACTCCGCAAGGAAGGACTATTGCCG
Blongssp-spacer154	BreCNCMI-4321ph 3	100.000	4.77e-11	TCGGCCAACTCCGCAAGGAAGGACTATTGCCG
Blongssp-spacer16	BreUMB0089ph2	100.000	1.24e-11	GAGAACGCGCGCAAGTTCCCCGAACTGTCG-GAA
Blongssp-spacer16	BlongBifido_06ph1	100.000	1.24e-11	GAGAACGCGCGCAAGTTCCCCGAACTGTCG-GAA
Blongssp-spacer16	BifPRI1ph2	100.000	1.24e-11	GAGAACGCGCGCAAGTTCCCCGAACTGTCG-GAA
Blongssp-spacer20	BreUMB0089ph2	100.000	1.09e-12	CTGCAGGAACTGTTCGCGCGTCTGAACGAG-GAATC
Blongssp-spacer20	BlongBifido_04ph1	100.000	1.09e-12	CTGCAGGAACTGTTCGCGCGTCTGAACGAG-GAATC
Blongssp-spacer32	BreCNCMI-4321ph 2	100.000	1.09e-12	TCGCCTGGACGAAAACCAAGGAAGCCTCAC-GCAAG
Blongssp-spacer32	Blongssp__3_mod ph1	100.000	1.09e-12	TCGCCTGGACGAAAACCAAGGAAGCCTCAC-GCAAG
Blongssp-spacer32	BlongMCC10129ph 1	100.000	1.09e-12	TCGCCTGGACGAAAACCAAGGAAGCCTCAC-GCAAG
Blongssp-spacer32	Bif85Bph2	100.000	1.09e-12	TCGCCTGGACGAAAACCAAGGAAGCCTCAC-GCAAG
Blongssp-spacer36	BlongF8ph1	100.000	1.24e-11	GAAAGCCTGTGCGCGATCGTGCTCAGGCCG-GAG
Blongssp-spacer36	BifBIO5480ph1	100.000	1.24e-11	GAAAGCCTGTGCGCGATCGTGCTCAGGCCG-GAG
Blongssp-spacer38	Blongssp__3_mod ph1	100.000	1.24e-11	CGCGACGAGGTCGGCAAATGGA-TACCCGCCGGC
Blongssp-spacer38	BlongMCC10129ph 1	100.000	1.24e-11	CGCGACGAGGTCGGCAAATGGA-TACCCGCCGGC
Blongssp-spacer38	BreCNCMI-4321ph 2	100.000	5.79e-10	GACGAGGTCGGCAAATGGATACCCGCCGGC
Blongssp-spacer38	BlongF8ph1	100.000	5.79e-10	GACGAGGTCGGCAAATGGATACCCGCCGGC
Blongssp-spacer40	BlongMCC10094ph 1	100.000	3.69e-12	TCGAAGCCGACGCCCTGACTGACCTTGAC-GACGG
Blongssp-spacer44	BreNRBB52ph4	100.000	1.24e-11	ACGGTCCCGTCCGGATACCTCTGCCGCCAC-CGG
Blongssp-spacer54	Brelw01ph1	100.000	1.09e-12	TCCAAGCAGTGGAAGGACTGG-CAGGCCGAGGGGAA
Blongssp-spacer58	BlongE18ph1	100.000	3.69e-12	TGCTGGTCTGGCAT-ACGTATGATCGGCTGCCGTC
Blongssp-spacer70	BlongTM01-1ph1	100.000	3.69e-12	CCGCAGTCTCGATTTGCGCCATGATTTGAC-GAAA
Blongssp-spacer70	BlongMCC10101ph 1	100.000	3.69e-12	CCGCAGTCTCGATTTGCGCCATGATTTGAC-GAAA
Blongssp-spacer70	BlongGT15ph3	100.000	3.69e-12	CCGCAGTCTCGATTTGCGCCATGATTTGAC-GAAA
Blongssp-spacer70	BlongDS15_3ph1	100.000	3.69e-12	CCGCAGTCTCGATTTGCGCCATGATTTGAC-GAAA
Blongssp-spacer70	BlongBB-79ph1	100.000	3.69e-12	CCGCAGTCTCGATTTGCGCCATGATTTGAC-

Spacer Name	Phage Name	Identity	E-value	Nucleotide Sequence
				GAAA
Blongssp-spacer70	BlongAF30-12ph2	100.000	3.69e-12	CCGCAGTCTCGATTTGCGCCATGATTTGAC-GAAA
Blongssp-spacer72	BreUMB0089ph2	100.000	4.47e-11	ATATCCCAGCTGCCAGGCAATATCTGGAACTG
Blongssp-spacer72	BlongBifido_04ph1	100.000	4.47e-11	ATATCCCAGCTGCCAGGCAATATCTGGAACTG
Blongssp-spacer74	BreNRBB52ph5	100.000	3.69e-12	CGCTTCACGTGGAGCCGACCATAGTCAAC-CAATC
Blongssp-spacer74	BreLMC520ph3	100.000	3.69e-12	CGCTTCACGTGGAGCCGACCATAGTCAAC-CAATC
Blongssp-spacer74	BreJR01ph1	100.000	3.69e-12	CGCTTCACGTGGAGCCGACCATAGTCAAC-CAATC
Blongssp-spacer74	BreDRBB28ph5	100.000	3.69e-12	CGCTTCACGTGGAGCCGACCATAGTCAAC-CAATC
Blongssp-spacer74	BreDRBB26ph1	100.000	3.69e-12	CGCTTCACGTGGAGCCGACCATAGTCAAC-CAATC
Blongssp-spacer74	BreBR3ph2	100.000	3.69e-12	CGCTTCACGTGGAGCCGACCATAGTCAAC-CAATC
Blongssp-spacer74	Bre082W48ph1	100.000	3.69e-12	CGCTTCACGTGGAGCCGACCATAGTCAAC-CAATC
Blongssp-spacer74	BlongLO-K29bph1	100.000	3.69e-12	CGCTTCACGTGGAGCCGACCATAGTCAAC-CAATC
Blongssp-spacer74	Blong-CCUG30698ph3	100.000	3.69e-12	CGCTTCACGTGGAGCCGACCATAGTCAAC-CAATC
Blongssp-spacer74	Blong72Bph1	100.000	3.69e-12	CGCTTCACGTGGAGCCGACCATAGTCAAC-CAATC
Blongssp-spacer80	BreNRBB52ph5	100.000	1.09e-12	CCGGCACCAGCGCCGAGGGCAACATCAC-CACCATC
Blongssp-spacer82	BreNRBB52ph5	100.000	1.24e-11	ACCGTGCTGCTGCGCATCTCCGACCGCGTCGGC
Blongssp-spacer82	BreMCC1605ph1	100.000	1.24e-11	ACCGTGCTGCTGCGCATCTCCGACCGCGTCGGC
Blongssp-spacer82	BreLMC520ph3	100.000	1.24e-11	ACCGTGCTGCTGCGCATCTCCGACCGCGTCGGC
Blongssp-spacer82	BreDRBB28ph5	100.000	1.24e-11	ACCGTGCTGCTGCGCATCTCCGACCGCGTCGGC
Blongssp-spacer82	BreDRBB26ph1	100.000	1.24e-11	ACCGTGCTGCTGCGCATCTCCGACCGCGTCGGC
Blongssp-spacer82	BreBR3ph2	100.000	1.24e-11	ACCGTGCTGCTGCGCATCTCCGACCGCGTCGGC
Blongssp-spacer82	Bre082W48ph1	100.000	1.24e-11	ACCGTGCTGCTGCGCATCTCCGACCGCGTCGGC
Blongssp-spacer82	Bre017W439ph2	100.000	1.24e-11	ACCGTGCTGCTGCGCATCTCCGACCGCGTCGGC
Blongssp-spacer82	BlongLO-K29bph1	100.000	1.24e-11	ACCGTGCTGCTGCGCATCTCCGACCGCGTCGGC
Blongssp-spacer82	Blong-CECT7210.2ph3	100.000	1.24e-11	ACCGTGCTGCTGCGCATCTCCGACCGCGTCGGC
Blongssp-spacer82	Blong-CCUG30698ph3	100.000	1.24e-11	ACCGTGCTGCTGCGCATCTCCGACCGCGTCGGC
Blongssp-spacer82	Blong157Fph1	100.000	1.24e-11	ACCGTGCTGCTGCGCATCTCCGACCGCGTCGGC
Blongssp-spacer86	BreMCC1605ph1	100.000	1.09e-12	CAGAAAGGGATAATCATGGCTTT-GGAAGTGAAGCG
Blongssp-spacer86	BreLMC520ph3	100.000	1.09e-12	CAGAAAGGGATAATCATGGCTTT-GGAAGTGAAGCG
Blongssp-spacer86	BreJR01ph1	100.000	1.09e-12	CAGAAAGGGATAATCATGGCTTT-GGAAGTGAAGCG
Blongssp-spacer86	BreDRBB28ph5	100.000	1.09e-12	CAGAAAGGGATAATCATGGCTTT-GGAAGTGAAGCG
Blongssp-spacer86	BreDRBB26ph1	100.000	1.09e-12	CAGAAAGGGATAATCATGGCTTT-GGAAGTGAAGCG
Blongssp-spacer86	BreBR3ph2	100.000	1.09e-12	CAGAAAGGGATAATCATGGCTTT-GGAAGTGAAGCG
Blongssp-spacer86	Bre082W48ph1	100.000	1.09e-12	CAGAAAGGGATAATCATGGCTTT-GGAAGTGAAGCG
Blongssp-spacer86	BlongLO-K29bph1	100.000	1.09e-12	CAGAAAGGGATAATCATGGCTTT-

Spacer Name	Phage Name	Identity	E-value	Nucleotide Sequence
				GGAAGTGAAGCG
Blongssp-spacer86	Blong-CECT7210.2ph3	100.000	1.09e-12	CAGAAAGGGATAATCATGGCTTT-GGAAGTGAAGCG
Blongssp-spacer86	Blong-CCUG30698ph3	100.000	1.09e-12	CAGAAAGGGATAATCATGGCTTT-GGAAGTGAAGCG
Blongssp-spacer86	Blong72Bph1	100.000	1.09e-12	CAGAAAGGGATAATCATGGCTTT-GGAAGTGAAGCG
Blongssp-spacer86	Blong157Fph1	100.000	1.09e-12	CAGAAAGGGATAATCATGGCTTT-GGAAGTGAAGCG
Blongssp-spacer92	Blon-gAM39-8ACph1	100.000	3.21e-13	TCCACAGCGAGCCTGTTCATGTCGTAC-CTGTCAAGC
Blongssp-spacer94	BlongMCC10040ph2	100.000	1.09e-12	CCTAACCCAGACCGAAGTGCTGACGCTGAT-TAACT
Blongssp-spacer94	Blong-CCUG30698ph3	100.000	1.09e-12	CCTAACCCAGACCGAAGTGCTGACGCTGAT-TAACT
Blongssp-spacer98	BlongTF07-39ph1	100.000	1.09e-12	ACTGATATGACCGTCTGAAAGATTATCTCCG-CAGA
Blongssp-spacer98	BlongTF07-31ph1	100.000	1.09e-12	ACTGATATGACCGTCTGAAAGATTATCTCCG-CAGA
Blongssp-spacer98	BlongTF06-12ACph1	100.000	1.09e-12	ACTGATATGACCGTCTGAAAGATTATCTCCG-CAGA
Blongssp-spacer98	BlongE18ph1	100.000	1.09e-12	ACTGATATGACCGTCTGAAAGATTATCTCCG-CAGA
Blongssp-spacer98	BlongAH1206ph2	100.000	1.09e-12	ACTGATATGACCGTCTGAAAGATTATCTCCG-CAGA
BlongTF01-22-spacer118	BlongU-MA3015ph1	100.000	9.42e-14	ACGGCATCAGCGGTGCCATCGGCG-GATTGTACGACAA
BlongTF01-22-spacer118	BlongMCC10116ph1	100.000	9.42e-14	ACGGCATCAGCGGTGCCATCGGCG-GATTGTACGACAA
BlongTF01-22-spacer118	BlongMCC10115ph1	100.000	9.42e-14	ACGGCATCAGCGGTGCCATCGGCG-GATTGTACGACAA
BlongTF01-22-spacer118	BlongMCC10100ph1	100.000	9.42e-14	ACGGCATCAGCGGTGCCATCGGCG-GATTGTACGACAA
BlongTF01-22-spacer118	BlongMCC10093ph1	100.000	9.42e-14	ACGGCATCAGCGGTGCCATCGGCG-GATTGTACGACAA
BlongTF01-22-spacer118	BlongMCC10077ph1	100.000	9.42e-14	ACGGCATCAGCGGTGCCATCGGCG-GATTGTACGACAA
BlongTF01-22-spacer118	BlongMCC10038ph1	100.000	9.42e-14	ACGGCATCAGCGGTGCCATCGGCG-GATTGTACGACAA
BlongTF01-22-spacer118	BlongMCC10111ph1	100.000	1.58e-11	ACGGCATCAGCGGTGCCATCGGCG-GATTGTACG
BlongTF01-22-spacer118	BlongMCC10040ph2	100.000	1.58e-11	ACGGCATCAGCGGTGCCATCGGCG-GATTGTACG
BlongTF01-22-spacer118	BlongLO-K29aph1	100.000	1.58e-11	ACGGCATCAGCGGTGCCATCGGCG-GATTGTACG
BlongTF01-22-spacer58	Blong-BIC1307292462ph1	100.000	1.24e-11	GTGTTGGTGGACCCGCAGCATGGTTGCGACCAT
BlongTF01-22-spacer58	Blon-gATCC15697.2ph1	100.000	1.24e-11	GTGTTGGTGGACCCGCAGCATGGTTGCGACCAT
BlongTF01-22-spacer76	BreDRBB28ph1	100.000	1.09e-12	GTCATGACACATCATGTGTCAC-GCCGCTAAACTTA
BlongTF01-22-spacer76	BreCNCMI-4321ph1	100.000	1.09e-12	GTCATGACACATCATGTGTCAC-GCCGCTAAACTTA
BlongTF01-22-spacer76	Bre689bph1	100.000	1.09e-12	GTCATGACACATCATGTGTCAC-GCCGCTAAACTTA
BlongTF07-31-spacer	BlongJSRL02ph2	100.000	4.47e-11	CCGCCAAGGGTGGAGCCCACCGAGACCAGGAT

Spacer Name	Phage Name	Identity	E-value	Nucleotide Sequence
7				
BlongTF07-31-spacer 7	BlongAF30-11ph1	100.000	4.47e-11	CCGCCAAGGGTGGAGCCCACCGAGACCAGGAT
BlongTF07-31-spacer 7	BadZJ2ph1	100.000	4.47e-11	CCGCCAAGGGTGGAGCCCACCGAGACCAGGAT
BlongTF07-31-spacer 7	BadTF06-2ACph1	100.000	4.47e-11	CCGCCAAGGGTGGAGCCCACCGAGACCAGGAT
BlongTF07-31-spacer 7	BadTF06-10ACph2	100.000	4.47e-11	CCGCCAAGGGTGGAGCCCACCGAGACCAGGAT
BlongTF07-31-spacer 7	BadAM12-59ph1	100.000	4.47e-11	CCGCCAAGGGTGGAGCCCACCGAGACCAGGAT
BlongTF07-31-spacer 7	BadAM12-20ph1	100.000	4.47e-11	CCGCCAAGGGTGGAGCCCACCGAGACCAGGAT
BlongTF07-31-spacer 7	BadAL46-7ph1	100.000	4.47e-11	CCGCCAAGGGTGGAGCCCACCGAGACCAGGAT
BlongTF07-31-spacer 7	BadAF28-4ACph1	100.000	4.47e-11	CCGCCAAGGGTGGAGCCCACCGAGACCAGGAT
BlongTF07-31-spacer 7	Bad22Lph1	100.000	4.47e-11	CCGCCAAGGGTGGAGCCCACCGAGACCAGGAT
BlongTF07-34-spacer 11	BlongTM01-1ph1	100.000	4.47e-11	AACGCGGTACGGTCGGGGATATCGCCGGCCCT
BlongTF07-34-spacer 11	BlongMCC10101ph 1	100.000	4.47e-11	AACGCGGTACGGTCGGGGATATCGCCGGCCCT
BlongTF07-34-spacer 11	BlongGT15ph3	100.000	4.47e-11	AACGCGGTACGGTCGGGGATATCGCCGGCCCT
BlongTF07-34-spacer 11	BlongDS15_3ph1	100.000	4.47e-11	AACGCGGTACGGTCGGGGATATCGCCGGCCCT
BlongTF07-34-spacer 11	BlongBB-79ph1	100.000	4.47e-11	AACGCGGTACGGTCGGGGATATCGCCGGCCCT
BlongTF07-34-spacer 11	BlongAF30-12ph2	100.000	4.47e-11	AACGCGGTACGGTCGGGGATATCGCCGGCCCT
BlongTF07-34-spacer 13	BlongTM01-1ph1	100.000	4.47e-11	CTCATGCTCGCCGAATCCGGTTCGAGCCTGAC
BlongTF07-34-spacer 13	BlongMCC10101ph 1	100.000	4.47e-11	CTCATGCTCGCCGAATCCGGTTCGAGCCTGAC
BlongTF07-34-spacer 13	BlongGT15ph3	100.000	4.47e-11	CTCATGCTCGCCGAATCCGGTTCGAGCCTGAC
BlongTF07-34-spacer 13	BlongDS15_3ph1	100.000	4.47e-11	CTCATGCTCGCCGAATCCGGTTCGAGCCTGAC
BlongTF07-34-spacer 13	BlongBB-79ph1	100.000	4.47e-11	CTCATGCTCGCCGAATCCGGTTCGAGCCTGAC
BlongTF07-34-spacer 13	BlongAF30-12ph2	100.000	4.47e-11	CTCATGCTCGCCGAATCCGGTTCGAGCCTGAC
BlongTF07-34-spacer 15	BlongTM01-1ph1	100.000	4.47e-11	CAACGGTTCGAGAAGAACATGAAGGGCGTCAA
BlongTF07-34-spacer 15	BlongMCC10101ph 1	100.000	4.47e-11	CAACGGTTCGAGAAGAACATGAAGGGCGTCAA
BlongTF07-34-spacer 15	BlongGT15ph3	100.000	4.47e-11	CAACGGTTCGAGAAGAACATGAAGGGCGTCAA
BlongTF07-34-spacer 15	BlongDS15_3ph1	100.000	4.47e-11	CAACGGTTCGAGAAGAACATGAAGGGCGTCAA
BlongTF07-34-spacer 15	BlongBB-79ph1	100.000	4.47e-11	CAACGGTTCGAGAAGAACATGAAGGGCGTCAA
BlongTF07-34-spacer 15	BlongAF30-12ph2	100.000	4.47e-11	CAACGGTTCGAGAAGAACATGAAGGGCGTCAA
BlongTF07-34-spacer	BlongTM01-1ph1	100.000	4.47e-11	GTCTGCGCGTGCTGCCCGCCTCGTTGGTGAGC

Spacer Name	Phage Name	Identity	E-value	Nucleotide Sequence
17				
BlongTF07-34-spacer 17	BlongMCC10101ph 1	100.000	4.47e-11	GTCTGCGCGTGCTGCCCGCCTCGTTGGTGAGC
BlongTF07-34-spacer 17	BlongGT15ph3	100.000	4.47e-11	GTCTGCGCGTGCTGCCCGCCTCGTTGGTGAGC
BlongTF07-34-spacer 17	BlongDS15_3ph1	100.000	4.47e-11	GTCTGCGCGTGCTGCCCGCCTCGTTGGTGAGC
BlongTF07-34-spacer 17	BlongBB-79ph1	100.000	4.47e-11	GTCTGCGCGTGCTGCCCGCCTCGTTGGTGAGC
BlongTF07-34-spacer 17	BlongAF30-12ph2	100.000	4.47e-11	GTCTGCGCGTGCTGCCCGCCTCGTTGGTGAGC
BlongTF07-34-spacer 43	BlongTM01-1ph1	100.000	4.47e-11	ACCACCATCACCAAAAGCTACGACGTGCGCAA
BlongTF07-34-spacer 43	BlongMCC10101ph 1	100.000	4.47e-11	ACCACCATCACCAAAAGCTACGACGTGCGCAA
BlongTF07-34-spacer 43	BlongGT15ph3	100.000	4.47e-11	ACCACCATCACCAAAAGCTACGACGTGCGCAA
BlongTF07-34-spacer 43	BlongDS15_3ph1	100.000	4.47e-11	ACCACCATCACCAAAAGCTACGACGTGCGCAA
BlongTF07-34-spacer 43	BlongBB-79ph1	100.000	4.47e-11	ACCACCATCACCAAAAGCTACGACGTGCGCAA
BlongTF07-34-spacer 43	BlongAF30-12ph2	100.000	4.47e-11	ACCACCATCACCAAAAGCTACGACGTGCGCAA
BlongTF07-34-spacer 45	BreDRBB28ph1	100.000	4.47e-11	TATCTGACCACGGCTCAGGCGGCGGAATACTT
BlongTF07-34-spacer 45	BreCNCMI-4321ph 1	100.000	4.47e-11	TATCTGACCACGGCTCAGGCGGCGGAATACTT
BlongTF07-34-spacer 45	Bre689bph1	100.000	4.47e-11	TATCTGACCACGGCTCAGGCGGCGGAATACTT
BlongTF07-34-spacer 57	BlongAM16-2ph1	100.000	4.47e-11	TTGAGCCGTGACCACGGCCGTCTCATCGGCAA
BlongTF07-34-spacer 7	BlongTM01-1ph1	100.000	4.47e-11	GCCCGCTGACGACCCCGCCGGCCCGTACCCCG
BlongTF07-34-spacer 7	BlongMCC10101ph 1	100.000	4.47e-11	GCCCGCTGACGACCCCGCCGGCCCGTACCCCG
BlongTF07-34-spacer 7	BlongGT15ph3	100.000	4.47e-11	GCCCGCTGACGACCCCGCCGGCCCGTACCCCG
BlongTF07-34-spacer 7	BlongDS15_3ph1	100.000	4.47e-11	GCCCGCTGACGACCCCGCCGGCCCGTACCCCG
BlongTF07-34-spacer 7	BlongBB-79ph1	100.000	4.47e-11	GCCCGCTGACGACCCCGCCGGCCCGTACCCCG
BlongTF07-34-spacer 7	BlongAF30-12ph2	100.000	4.47e-11	GCCCGCTGACGACCCCGCCGGCCCGTACCCCG
BlongTF07-34-spacer 9	BlongTM01-1ph1	100.000	4.47e-11	CATTTTCATAGCCTGATTTTGGGGTGCCGTCC
BlongTF07-34-spacer 9	BlongGT15ph3	100.000	4.47e-11	CATTTTCATAGCCTGATTTTGGGGTGCCGTCC
BlongTF07-34-spacer 9	BlongDS15_3ph1	100.000	4.47e-11	CATTTTCATAGCCTGATTTTGGGGTGCCGTCC
BlongTF07-34-spacer 9	BlongBB-79ph1	100.000	4.47e-11	CATTTTCATAGCCTGATTTTGGGGTGCCGTCC
BlongTF07-34-spacer 9	BlongAF30-12ph2	100.000	4.47e-11	CATTTTCATAGCCTGATTTTGGGGTGCCGTCC
BlongTF07-34-spacer 99	Blong-CECT7210.2ph3	100.000	4.47e-11	GATACGGGCTCGAATGGGGCGACCTCGACCTG
BlongTF07-34-spacer	Blong-	100.000	4.47e-11	GATACGGGCTCGAATGGGGCGACCTCGACCTG

Spacer Name	Phage Name	Identity	E-value	Nucleotide Sequence
99	CCUG30698ph3			
BlongTF08-4AC-spacer44	Blong105-Aph2	98.020	8.17e-45	CGAGTCGAGGACCTCGACCTCGAGCGCGCG-GAGGCCCAC-GATCACCCCTCTGCCCGCGTTCGCCCGGTCCGCGGAATAACGATGCCGTCCC-CGGGCGTGA
BlongTF08-4AC-spacer45	BlongBORIph1	100.000	6.15e-23	CCACGCCGTACCGGTCGGCCTTCAT-ATGCCGCCACGTGACCACGTCGAAACGCT
BlongTF08-4AC-spacer45	Blong105-Aph2	100.000	6.15e-23	CCACGCCGTACCGGTCGGCCTTCAT-ATGCCGCCACGTGACCACGTCGAAACGCT
BlongTF08-4AC-spacer48	BlongBORIph1	98.000	8.17e-45	TCCCGGGCAACGGCAGCAGCGCCGCAC-GGTCCTCGGAGAACAGGCCAC-GTTCCTCCGTGTCCTTGCGGCAG-TGCGGTTTCGTGGCCAGCTCCAGGCACCG
BlongTF08-4AC-spacer48	Blong105-Aph2	98.000	8.17e-45	TCCCGGGCAACGGCAGCAGCGCCGCAC-GGTCCTCGGAGAACAGGCCAC-GTTCCTCCGTGTCCTTGCGGCAG-TGCGGTTTCGTGGCCAGCTCCAGGCACCG
Bre017W439-spacer0	BreDRBB28ph5	100.000	3.77e-48	CTCGTATTTGCCATGGATGGTGAACCATTT-GTCCGCGATCTGCATGCTGTTCAGGCCTT-GGAGCACTCCGATGGCCTGATCGTCGTTT-GCATAGATATAG
Bre017W439-spacer0	BreDRBB26ph1	100.000	3.77e-48	CTCGTATTTGCCATGGATGGTGAACCATTT-GTCCGCGATCTGCATGCTGTTCAGGCCTT-GGAGCACTCCGATGGCCTGATCGTCGTTT-GCATAGATATAG
Bre017W439-spacer0	BreJR01ph1	99.000	1.76e-46	CTCGTATTTGCCATGGATGGTGAACCATTT-GTCCGCGATCTGCATGCTGTTCAGGCCTT-GGAGCACTCCGATGGCCTGATCGTCGTTT-GCATAGATATAG
Bre017W439-spacer2	BreJR01ph1	100.000	1.76e-23	GCCGTTCGCTTCGGCCACCTTGGCCAG-CATGTCGCTGTTGTCCCCCTGCAGCAAC
Bre017W439-spacer2	BreDRBB28ph5	100.000	1.76e-23	GCCGTTCGCTTCGGCCACCTTGGCCAG-CATGTCGCTGTTGTCCCCCTGCAGCAAC
Bre017W439-spacer2	BreDRBB26ph1	100.000	1.76e-23	GCCGTTCGCTTCGGCCACCTTGGCCAG-CATGTCGCTGTTGTCCCCCTGCAGCAAC
Bre017W439-spacer6	BreJR01ph1	100.000	3.77e-48	TCCGGTGGCGTCTGCGATCTTCTG-CATCAGGTCGGTGTTGTCGCCGCTGATGCG-CACGGTCTTGTCATCGAT-TTTCTCGGCCTTCACCTTCACCTTGTCC
Bre017W439-spacer6	BreDRBB28ph5	100.000	3.77e-48	TCCGGTGGCGTCTGCGATCTTCTG-CATCAGGTCGGTGTTGTCGCCGCTGATGCG-CACGGTCTTGTCATCGAT-TTTCTCGGCCTTCACCTTCACCTTGTCC
Bre017W439-spacer6	BreDRBB26ph1	100.000	3.77e-48	TCCGGTGGCGTCTGCGATCTTCTG-CATCAGGTCGGTGTTGTCGCCGCTGATGCG-CACGGTCTTGTCATCGAT-TTTCTCGGCCTTCACCTTCACCTTGTCC
Bre082W48-spacer28	BreSC95ph1	100.000	8.17e-45	GGAGGGGGCTACAGGG-GAGGTAAGGCTAGGTATGGTTAGGTTAGGAC-CGGTTGCTTCGTTTGCTTCGAAGCAATT-GCTTCGTTTTGCTTCGGAC
Bre12-4-spacer116	BreDRBB28ph1	100.000	3.69e-12	CGTTCCTCGACAAGGGAGCCGTGAC-CGTGTGGGA
Bre12-4-spacer116	BreCNCMI-4321ph1	100.000	3.69e-12	CGTTCCTCGACAAGGGAGCCGTGAC-CGTGTGGGA
Bre12-4-spacer116	Bre689bph1	100.000	3.69e-12	CGTTCCTCGACAAGGGAGCCGTGAC-

Spacer Name	Phage Name	Identity	E-value	Nucleotide Sequence
				CGTGTGGGA
Bre12-4-spacer116	BlongAF36-1ph1	100.000	3.69e-12	CGTTCCTCGACAAGGGAGCCGTGAC-CGTGTGGGA
Bre12-4-spacer116	Blon-gAF35-13ACph2	100.000	3.69e-12	CGTTCCTCGACAAGGGAGCCGTGAC-CGTGTGGGA
Bre12-4-spacer118	BreDRBB28ph1	100.000	3.69e-12	TGCAGACCATGAACTTCGCGGGCATCGACAC-GTC
Bre12-4-spacer118	BreCNCMI-4321ph1	100.000	3.69e-12	TGCAGACCATGAACTTCGCGGGCATCGACAC-GTC
Bre12-4-spacer118	Bre689bph1	100.000	3.69e-12	TGCAGACCATGAACTTCGCGGGCATCGACAC-GTC
Bre12-4-spacer118	Blon-gAF35-13ACph2	100.000	3.69e-12	TGCAGACCATGAACTTCGCGGGCATCGACAC-GTC
Bre12-4-spacer20	BreDRBB30ph3	100.000	3.21e-13	GACGTTCTT-GCCCAGGCTGATCCTGCGATTGACCCT
Bre12-4-spacer20	BreCNCMI-4321ph3	100.000	3.21e-13	GACGTTCTT-GCCCAGGCTGATCCTGCGATTGACCCT
Bre12-4-spacer20	BadAF45-19ph1	100.000	3.21e-13	GACGTTCTT-GCCCAGGCTGATCCTGCGATTGACCCT
Bre12-4-spacer20	BadAF21-27ph1	100.000	3.21e-13	GACGTTCTT-GCCCAGGCTGATCCTGCGATTGACCCT
Bre12-4-spacer22	BreDRBB30ph3	100.000	3.21e-13	CCAGACACCACGACCCCGGCTGCGGTACAC-GATACG
Bre12-4-spacer22	BreCNCMI-4321ph3	100.000	3.21e-13	CCAGACACCACGACCCCGGCTGCGGTACAC-GATACG
Bre12-4-spacer28	BreDRBB30ph3	100.000	3.69e-12	TCGCCGTCCGCTCTCCCAAAGTCCGGGTTTTCGC
Bre12-4-spacer28	BreCNCMI-4321ph3	100.000	3.69e-12	TCGCCGTCCGCTCTCCCAAAGTCCGGGTTTTCGC
Bre12-4-spacer36	BreDRBB28ph1	100.000	3.69e-12	TTTTCGGGCTTTTTTATTTGGTTTGTGGGGATAA
Bre12-4-spacer36	BreCNCMI-4321ph1	100.000	3.69e-12	TTTTCGGGCTTTTTTATTTGGTTTGTGGGGATAA
Bre12-4-spacer36	Bre689bph1	100.000	3.69e-12	TTTTCGGGCTTTTTTATTTGGTTTGTGGGGATAA
Bre12-4-spacer36	BlongAF36-1ph1	100.000	3.69e-12	TTTTCGGGCTTTTTTATTTGGTTTGTGGGGATAA
Bre12-4-spacer38	BreDRBB28ph1	100.000	3.69e-12	AGGTTGGTGATGCGGCGGCGCTGTTCGAG-GATGC
Bre12-4-spacer38	BreCNCMI-4321ph1	100.000	3.69e-12	AGGTTGGTGATGCGGCGGCGCTGTTCGAG-GATGC
Bre12-4-spacer38	Bre689bph1	100.000	3.69e-12	AGGTTGGTGATGCGGCGGCGCTGTTCGAG-GATGC
Bre12-4-spacer38	BlongAF36-1ph1	100.000	3.69e-12	AGGTTGGTGATGCGGCGGCGCTGTTCGAG-GATGC
Bre12-4-spacer38	Blon-gAF35-13ACph2	100.000	3.69e-12	AGGTTGGTGATGCGGCGGCGCTGTTCGAG-GATGC
Bre12-4-spacer40	BreDRBB28ph1	100.000	3.21e-13	GTAGCTTTCCTCGACGTGCCCGCCGAAC-CGGCCGGG
Bre12-4-spacer40	BreCNCMI-4321ph1	100.000	3.21e-13	GTAGCTTTCCTCGACGTGCCCGCCGAAC-CGGCCGGG
Bre12-4-spacer40	Bre689bph1	100.000	3.21e-13	GTAGCTTTCCTCGACGTGCCCGCCGAAC-CGGCCGGG
Bre12-4-spacer57	BreDRBB30ph3	100.000	5.07e-11	CAGCCCTCGCTGCCGGTCTGAGCCTTGCCGTA
Bre12-4-spacer57	BreCNCMI-4321ph3	100.000	5.07e-11	CAGCCCTCGCTGCCGGTCTGAGCCTTGCCGTA
Bre12-4-spacer96	Brelw01ph1	100.000	1.09e-12	GTTTTAATGTGATTTAATGGACTGTT-GCTGAATT
Bre139W423-spacer4	BadBB23ph2	100.000	2.08e-09	CCCTTCGCGATGTTCTGGCCGATTTCGTC

Spacer Name	Phage Name	Identity	E-value	Nucleotide Sequence
2				
Bre1889B-spacer106	BreDRBB30ph3	100.000	3.69e-12	CGGGGCAGGCTGTCGCGGA-GAAATGGCTGTGCGG
Bre1889B-spacer106	BreCNCMI-4321ph3	100.000	3.69e-12	CGGGGCAGGCTGTCGCGGA-GAAATGGCTGTGCGG
Bre1889B-spacer114	BreDRBB27ph4	100.000	4.47e-11	GCGGTGTTCGCCATGCCCCAGGTGTGGTGGCG
Bre1889B-spacer116	BreDRBB28ph1	100.000	1.09e-12	CCAGCCGTCTGGGTGAGCATCATCGCCAC-GCTTTT
Bre1889B-spacer116	BreCNCMI-4321ph1	100.000	1.09e-12	CCAGCCGTCTGGGTGAGCATCATCGCCAC-GCTTTT
Bre1889B-spacer116	Bre689bph1	100.000	1.09e-12	CCAGCCGTCTGGGTGAGCATCATCGCCAC-GCTTTT
Bre1889B-spacer116	Blon-gAF35-13ACph2	100.000	1.09e-12	CCAGCCGTCTGGGTGAGCATCATCGCCAC-GCTTTT
Bre1889B-spacer126	BreNRBB01ph2	100.000	1.09e-12	TGTTCCAGCCGGTGCTTGAGGATGACGGCAC-CGAT
Bre1889B-spacer126	BreDSM20213.3ph2	100.000	1.09e-12	TGTTCCAGCCGGTGCTTGAGGATGACGGCAC-CGAT
Bre1889B-spacer128	BreBIO6018ph1	100.000	1.09e-12	CTGAAATCGTTCGCCGACTGCTTGAAAC-CGTTTTT
Bre1889B-spacer128	BlongTF06-45Aph3	100.000	1.09e-12	CTGAAATCGTTCGCCGACTGCTTGAAAC-CGTTTTT
Bre1889B-spacer128	BlongAF08-2ph1	100.000	1.09e-12	CTGAAATCGTTCGCCGACTGCTTGAAAC-CGTTTTT
Bre1889B-spacer60	BreDRBB30ph3	100.000	3.69e-12	CCATGATTTACTTCTCCTTTTCTTCGGTGGCTTT
Bre1889B-spacer60	BreCNCMI-4321ph3	100.000	3.69e-12	CCATGATTTACTTCTCCTTTTCTTCGGTGGCTTT
Bre1889B-spacer76	Bre689bph1	100.000	3.21e-13	GTGGTGTTGCCCGACACGTTCAC-CGTGGTGCTGGAC
Bre1889B-spacer80	BreDRBB28ph1	100.000	1.24e-11	CAGACCGGCCGGCGCAATGATGACCCAA-GCATG
Bre1889B-spacer80	BreCNCMI-4321ph1	100.000	1.24e-11	CAGACCGGCCGGCGCAATGATGACCCAA-GCATG
Bre1889B-spacer96	Bif85Bph2	100.000	1.09e-12	GCCGTGTCATGGCCCAGCTCTTGGCGTGG-TACCAG
Bre1900B-spacer119	BreCNCMI-4321ph2	100.000	1.09e-12	TTCCATTTGCCGGCGGTGTCCTCGCTGGCATA-GAC
Bre1900B-spacer119	Blongssp__3_modph1	100.000	1.09e-12	TTCCATTTGCCGGCGGTGTCCTCGCTGGCATA-GAC
Bre1900B-spacer119	BlongF8ph1	100.000	1.41e-11	CCATTTGCCGGCGGTGTCCTCGCTGGCATAGAC
Bre1900B-spacer119	BlongMCC10129ph1	100.000	2.36e-09	TTCCATTTGCCGGCGGTGTCCTCGCTGGC
Bre1900B-spacer121	BlongF8ph1	100.000	1.09e-12	TCTGCCGTCAGTTCGACGAACTTGCG-CAGCTCACC
Bre1900B-spacer121	BlongBG7ph1	100.000	1.09e-12	TCTGCCGTCAGTTCGACGAACTTGCG-CAGCTCACC
Bre1900B-spacer125	BreCNCMI-4321ph2	100.000	9.42e-14	TCGCGCACAGGCTTTCGAACGTGTCCTT-GATGACGCT
Bre1900B-spacer125	Blongssp__3_modph1	100.000	9.42e-14	TCGCGCACAGGCTTTCGAACGTGTCCTT-GATGACGCT
Bre1900B-spacer125	BlongMCC10129ph1	100.000	9.42e-14	TCGCGCACAGGCTTTCGAACGTGTCCTT-GATGACGCT
Bre1900B-spacer125	BlongF8ph1	100.000	9.42e-14	TCGCGCACAGGCTTTCGAACGTGTCCTT-GATGACGCT
Bre1900B-spacer125	BlongBG7ph1	100.000	9.42e-14	TCGCGCACAGGCTTTCGAACGTGTCCTT-GATGACGCT

Spacer Name	Phage Name	Identity	E-value	Nucleotide Sequence
Bre1900B-spacer125	BifBIO5480ph1	100.000	9.42e-14	TCGCGCACAGGCTTTCGAACGTGTCCTT-GATGACGCT
Bre1900B-spacer127	BlongF8ph1	100.000	3.69e-12	CAAGGACGG-CACCCGCCATCCCTGCCCGCCACCC
Bre1900B-spacer127	BlongBG7ph1	100.000	3.69e-12	CAAGGACGG-CACCCGCCATCCCTGCCCGCCACCC
Bre1900B-spacer44	BreDRBB30ph3	100.000	5.07e-11	GCGGTGTCCTTGTCGGACACGCCACCCTGCGA
Bre1900B-spacer44	BreCNCMI-4321ph3	100.000	5.07e-11	GCGGTGTCCTTGTCGGACACGCCACCCTGCGA
Bre1900B-spacer46	BreDRBB30ph3	100.000	3.69e-12	ACGGCATCGCAAGA-CACAGGCCGCGCTCGCCAAG
Bre1900B-spacer46	BreCNCMI-4321ph3	100.000	3.69e-12	ACGGCATCGCAAGA-CACAGGCCGCGCTCGCCAAG
Bre1900B-spacer48	BreBIO6018ph1	100.000	3.21e-13	GCGCACCACCGCGTCGCCATAGTCGGACAG-GAGGTT
Bre1900B-spacer48	BlongTF06-45Aph3	100.000	3.21e-13	GCGCACCACCGCGTCGCCATAGTCGGACAG-GAGGTT
Bre1900B-spacer48	BlongAF08-2ph1	100.000	3.21e-13	GCGCACCACCGCGTCGCCATAGTCGGACAG-GAGGTT
Bre1900B-spacer50	BreNRBB01ph2	100.000	1.09e-12	TTGCGCAAGTGCGGTGGGTCGAGCACGAC-CATGCG
Bre1900B-spacer50	BreDSM20213.3ph2	100.000	1.09e-12	TTGCGCAAGTGCGGTGGGTCGAGCACGAC-CATGCG
Bre1900B-spacer50	BreDRBB30ph3	100.000	1.09e-12	TTGCGCAAGTGCGGTGGGTCGAGCACGAC-CATGCG
Bre1900B-spacer50	BreCNCMI-4321ph3	100.000	1.09e-12	TTGCGCAAGTGCGGTGGGTCGAGCACGAC-CATGCG
Bre1900B-spacer77	BreBR-19ph1	100.000	3.77e-48	TAACCGACAACCCACAGGACTGGCCAAA-GCCGAAACGGCCTAAAAACAAC-CGCTCAAACGGACTAAAAGAAAC-GCCGAAACGGCCCACAAAAATTTGACA
Bre1900B-spacer77	BreBR-15ph1	100.000	3.77e-48	TAACCGACAACCCACAGGACTGGCCAAA-GCCGAAACGGCCTAAAAACAAC-CGCTCAAACGGACTAAAAGAAAC-GCCGAAACGGCCCACAAAAATTTGACA
Bre1900B-spacer79	BreNRBB51ph1	100.000	3.21e-13	GGTCATCAGCATGTTCTTCG-GAATCATCATCACCAT
Bre1900B-spacer81	BlongF8ph1	100.000	4.47e-11	GTGTTACGCTGTCGCGTGTAATCGGAGTATCG
Bre1900B-spacer81	BlongBG7ph1	100.000	4.47e-11	GTGTTACGCTGTCGCGTGTAATCGGAGTATCG
Bre1900B-spacer83	BreCNCMI-4321ph2	100.000	3.39e-13	GTCGCGTTTGATTTGGGTGAGGTCCTTGCCG-TATTC
Bre1900B-spacer83	Blongssp__3_modph1	100.000	3.39e-13	GTCGCGTTTGATTTGGGTGAGGTCCTTGCCG-TATTC
Bre1900B-spacer83	Bif85Bph2	100.000	3.39e-13	GTCGCGTTTGATTTGGGTGAGGTCCTTGCCG-TATTC
Bre1900B-spacer83	BlongMCC10129ph1	100.000	1.58e-11	GTCGCGTTTGATTTGGGTGAGGTCCTTGCCGTA
Bre1900B-spacer87	Blong157Fph1	100.000	1.24e-11	CGGACGAGGCGGTGGCCATTACAC-GGCTCAGCA
Bre1900B-spacer95	BreNRBB52ph5	100.000	3.69e-12	AGGTTCGCGCCGTCAGCACCAC-CGCCGACAGCGT
Bre1900B-spacer95	BreLMC520ph3	100.000	3.69e-12	AGGTTCGCGCCGTCAGCACCAC-CGCCGACAGCGT
Bre1900B-spacer95	BreBR3ph2	100.000	3.69e-12	AGGTTCGCGCCGTCAGCACCAC-CGCCGACAGCGT
Bre1900B-spacer95	BlongPC4ph1	100.000	3.69e-12	AGGTTCGCGCCGTCAGCACCAC-

Spacer Name	Phage Name	Identity	E-value	Nucleotide Sequence
				CGCCGACAGCGT
Bre1900B-spacer95	Blong-CECT7210.2ph3	100.000	3.69e-12	AGGTTCGCGCCGTCAGCACCAC-CGCCGACAGCGT
Bre1900B-spacer95	Blong-CCUG30698ph3	100.000	3.69e-12	AGGTTCGCGCCGTCAGCACCAC-CGCCGACAGCGT
Bre1900B-spacer95	Blong72Bph1	100.000	3.69e-12	AGGTTCGCGCCGTCAGCACCAC-CGCCGACAGCGT
Bre1900B-spacer95	Blong157Fph1	100.000	1.72e-10	AGGTTCGCGCCGTCAGCACCACCGCCGACAG
Bre31L-spacer132	BlongMCC10081ph1	100.000	1.09e-12	CCGGGACGTGGTGTCGGCGGAG-GAAGGCCACGCAA
BreACS-071-V-Sch8b-spacer117	BreDRBB28ph1	100.000	1.24e-11	TTACCGGCCAAACCCGCCTCACGATCCATGTCT
BreACS-071-V-Sch8b-spacer117	BreCNCMI-4321ph1	100.000	1.24e-11	TTACCGGCCAAACCCGCCTCACGATCCATGTCT
BreACS-071-V-Sch8b-spacer117	Bre689bph1	100.000	1.24e-11	TTACCGGCCAAACCCGCCTCACGATCCATGTCT
BreACS-071-V-Sch8b-spacer117	BlongAF36-1ph1	100.000	4.47e-11	TTACCGGCCAAACCCGCCTCACGATCCATGTC
BreACS-071-V-Sch8b-spacer117	Blon-gAF35-13ACph2	100.000	4.47e-11	TTACCGGCCAAACCCGCCTCACGATCCATGTC
BreACS-071-V-Sch8b-spacer119	BreDRBB28ph1	100.000	1.09e-12	TTGCCGTCGATGGTCGGG-GAGCCGTGCCCGGTGAG
BreACS-071-V-Sch8b-spacer119	BreCNCMI-4321ph1	100.000	1.09e-12	TTGCCGTCGATGGTCGGG-GAGCCGTGCCCGGTGAG
BreACS-071-V-Sch8b-spacer119	Bre689bph1	100.000	1.09e-12	TTGCCGTCGATGGTCGGG-GAGCCGTGCCCGGTGAG
BreACS-071-V-Sch8b-spacer121	BreDRBB28ph1	100.000	1.09e-12	CCGGTCGATGCCGGTCGTGCTTGTCGGTTT-GTCGA
BreACS-071-V-Sch8b-spacer121	BreCNCMI-4321ph1	100.000	1.09e-12	CCGGTCGATGCCGGTCGTGCTTGTCGGTTT-GTCGA
BreACS-071-V-Sch8b-spacer121	Bre689bph1	100.000	1.09e-12	CCGGTCGATGCCGGTCGTGCTTGTCGGTTT-GTCGA
BreACS-071-V-Sch8b-spacer123	BreDRBB28ph1	100.000	1.24e-11	CACCAAGCTCCGCGCGTTGATTCTCGCGGTGTT
BreACS-071-V-Sch8b-spacer123	BreCNCMI-4321ph1	100.000	1.24e-11	CACCAAGCTCCGCGCGTTGATTCTCGCGGTGTT
BreACS-071-V-Sch8b-spacer123	Bre689bph1	100.000	1.24e-11	CACCAAGCTCCGCGCGTTGATTCTCGCGGTGTT
BreACS-071-V-Sch8b-spacer125	BreDRBB28ph1	100.000	3.69e-12	CGAAGGCAC-GCGCCCAACCCGCCGCGTCCTGGCT
BreACS-071-V-Sch8b-spacer125	BreCNCMI-4321ph1	100.000	3.69e-12	CGAAGGCAC-GCGCCCAACCCGCCGCGTCCTGGCT
BreACS-071-V-Sch8b-spacer125	Bre689bph1	100.000	3.69e-12	CGAAGGCAC-GCGCCCAACCCGCCGCGTCCTGGCT
BreACS-071-V-Sch8b-spacer125	BlongAF36-1ph1	100.000	3.69e-12	CGAAGGCAC-GCGCCCAACCCGCCGCGTCCTGGCT
BreACS-071-V-Sch8b-spacer125	Blon-gAF35-13ACph2	100.000	4.77e-11	CGAAGGCACGCGCCCAACCCGCCGCGTCCTGG
BreACS-071-V-Sch8b-spacer127	BreDRBB28ph1	100.000	3.69e-12	GCAACGACTGTTGGCTGGACATGCCGG-GATGCAC
BreACS-071-V-Sch8b-spacer127	BreCNCMI-4321ph1	100.000	3.69e-12	GCAACGACTGTTGGCTGGACATGCCGG-GATGCAC
BreACS-071-V-Sch8b-spacer127	Bre689bph1	100.000	3.69e-12	GCAACGACTGTTGGCTGGACATGCCGG-GATGCAC
BreACS-071-V-Sch8b-spacer127	BlongAF36-1ph1	100.000	3.69e-12	GCAACGACTGTTGGCTGGACATGCCGG-GATGCAC

Spacer Name	Phage Name	Identity	E-value	Nucleotide Sequence
BreACS-071-V-Sch8b-spacer127	Blon-gAF35-13ACph2	100.000	3.69e-12	GCAACGACTGTTGGCTGGACATGCCGG-GATGCAC
BreACS-071-V-Sch8b-spacer129	BreDRBB28ph1	100.000	3.69e-12	CCAATGCGGAGAGGATACGGCCTGG-GATGCTGGA
BreACS-071-V-Sch8b-spacer129	BreCNCMI-4321ph1	100.000	3.69e-12	CCAATGCGGAGAGGATACGGCCTGG-GATGCTGGA
BreACS-071-V-Sch8b-spacer129	Bre689bph1	100.000	3.69e-12	CCAATGCGGAGAGGATACGGCCTGG-GATGCTGGA
BreACS-071-V-Sch8b-spacer129	Blon-gAF35-13ACph2	100.000	3.69e-12	CCAATGCGGAGAGGATACGGCCTGG-GATGCTGGA
BreACS-071-V-Sch8b-spacer131	BreDRBB28ph1	100.000	3.69e-12	TGAGCTGGCCCGCATACCAGAAC-GCCAAGGTGGA
BreACS-071-V-Sch8b-spacer131	BreCNCMI-4321ph1	100.000	3.69e-12	TGAGCTGGCCCGCATACCAGAAC-GCCAAGGTGGA
BreACS-071-V-Sch8b-spacer131	Bre689bph1	100.000	3.69e-12	TGAGCTGGCCCGCATACCAGAAC-GCCAAGGTGGA
BreACS-071-V-Sch8b-spacer131	BlongAF36-1ph1	100.000	3.69e-12	TGAGCTGGCCCGCATACCAGAAC-GCCAAGGTGGA
BreACS-071-V-Sch8b-spacer131	Blon-gAF35-13ACph2	100.000	3.69e-12	TGAGCTGGCCCGCATACCAGAAC-GCCAAGGTGGA
BreACS-071-V-Sch8b-spacer133	BreDRBB28ph1	100.000	1.24e-11	TGCCAGTGGTGCTCATGCCCGGCGCGGCCG-CAG
BreACS-071-V-Sch8b-spacer133	BreCNCMI-4321ph1	100.000	1.24e-11	TGCCAGTGGTGCTCATGCCCGGCGCGGCCG-CAG
BreACS-071-V-Sch8b-spacer133	Bre689bph1	100.000	1.24e-11	TGCCAGTGGTGCTCATGCCCGGCGCGGCCG-CAG
BreACS-071-V-Sch8b-spacer133	BlongAF36-1ph1	100.000	1.24e-11	TGCCAGTGGTGCTCATGCCCGGCGCGGCCG-CAG
BreACS-071-V-Sch8b-spacer137	BreDRBB28ph1	100.000	1.09e-12	CCGGCCACGCCGCTGCCGGTGGGAG-CACCCTGGGT
BreACS-071-V-Sch8b-spacer137	BreCNCMI-4321ph1	100.000	1.09e-12	CCGGCCACGCCGCTGCCGGTGGGAG-CACCCTGGGT
BreACS-071-V-Sch8b-spacer137	Bre689bph1	100.000	1.09e-12	CCGGCCACGCCGCTGCCGGTGGGAG-CACCCTGGGT
BreACS-071-V-Sch8b-spacer139	BreDRBB28ph1	100.000	1.09e-12	TTCTTCGGCGTCCCGTTGATGGCTTTGCTG-GATGA
BreACS-071-V-Sch8b-spacer141	BreDRBB28ph1	100.000	3.69e-12	TCGCGCAACGCTTCGGCCTCGGAAC-GATCCAGAG
BreACS-071-V-Sch8b-spacer141	Bre689bph1	100.000	3.69e-12	TCGCGCAACGCTTCGGCCTCGGAAC-GATCCAGAG
BreACS-071-V-Sch8b-spacer143	BreDRBB28ph1	100.000	3.21e-13	CTGATTGCCGGCCCAGGCGTTGC-TATCGTTCCTGCG
BreACS-071-V-Sch8b-spacer143	BreCNCMI-4321ph1	100.000	3.21e-13	CTGATTGCCGGCCCAGGCGTTGC-TATCGTTCCTGCG
BreACS-071-V-Sch8b-spacer143	Bre689bph1	100.000	3.21e-13	CTGATTGCCGGCCCAGGCGTTGC-TATCGTTCCTGCG
BreACS-071-V-Sch8b-spacer145	BreDRBB28ph1	100.000	1.09e-12	GTGGCAGGCCAAGGACGCGAGAAACGG-CATCGTGG
BreACS-071-V-Sch8b-spacer145	BreCNCMI-4321ph1	100.000	1.09e-12	GTGGCAGGCCAAGGACGCGAGAAACGG-CATCGTGG
BreACS-071-V-Sch8b-spacer145	Bre689bph1	100.000	1.09e-12	GTGGCAGGCCAAGGACGCGAGAAACGG-CATCGTGG
BreACS-071-V-Sch8b-spacer145	Blon-gAF35-13ACph2	100.000	1.09e-12	GTGGCAGGCCAAGGACGCGAGAAACGG-CATCGTGG
BreACS-071-V-Sch8b-spacer147	BreDRBB28ph1	100.000	1.24e-11	TAGCGTCAACGAG-CATGTCCGCCATGCTCAACG

Spacer Name	Phage Name	Identity	E-value	Nucleotide Sequence
BreACS-071-V-Sch8b-spacer147	BreCNCMI-4321ph1	100.000	1.24e-11	TAGCGTCAACGAG-CATGTCCGCCATGCTCAACG
BreACS-071-V-Sch8b-spacer149	BlongTM01-1ph1	100.000	1.09e-12	CTGCCGAAGGCGGCGGCTTTGCCGGTGAC-CTTGCT
BreACS-071-V-Sch8b-spacer149	BlongMCC10101ph1	100.000	1.09e-12	CTGCCGAAGGCGGCGGCTTTGCCGGTGAC-CTTGCT
BreACS-071-V-Sch8b-spacer149	BlongGT15ph3	100.000	1.09e-12	CTGCCGAAGGCGGCGGCTTTGCCGGTGAC-CTTGCT
BreACS-071-V-Sch8b-spacer149	BlongDS15_3ph1	100.000	1.09e-12	CTGCCGAAGGCGGCGGCTTTGCCGGTGAC-CTTGCT
BreACS-071-V-Sch8b-spacer149	BlongBB-79ph1	100.000	1.09e-12	CTGCCGAAGGCGGCGGCTTTGCCGGTGAC-CTTGCT
BreACS-071-V-Sch8b-spacer149	BlongAM16-2ph1	100.000	1.09e-12	CTGCCGAAGGCGGCGGCTTTGCCGGTGAC-CTTGCT
BreACS-071-V-Sch8b-spacer149	BlongAF30-12ph2	100.000	1.09e-12	CTGCCGAAGGCGGCGGCTTTGCCGGTGAC-CTTGCT
BreBifido_1-spacer51	BreDRBB28ph1	100.000	3.69e-12	AAGTCCCTGGGCGAA-GCCCGCCATGATGGCTTTG
BreBifido_1-spacer51	BreCNCMI-4321ph1	100.000	3.69e-12	AAGTCCCTGGGCGAA-GCCCGCCATGATGGCTTTG
BreBifido_1-spacer51	Bre689bph1	100.000	3.69e-12	AAGTCCCTGGGCGAA-GCCCGCCATGATGGCTTTG
BreBifido_1-spacer51	Blon-gAF35-13ACph2	100.000	3.69e-12	AAGTCCCTGGGCGAA-GCCCGCCATGATGGCTTTG
BreBifido_1-spacer53	Brelw01ph1	100.000	1.09e-12	TCGTCACGCAGGGAGTATTCGACGGCCAC-CTCGCA
BreBifido_1-spacer59	BreNRBB01ph2	100.000	1.09e-12	AGAACGAGGAAGTAGTGGCCACCGTCCTT-GAAATG
BreBifido_1-spacer59	BreDSM20213.3ph2	100.000	1.09e-12	AGAACGAGGAAGTAGTGGCCACCGTCCTT-GAAATG
BreBifido_1-spacer61	BreDRBB28ph1	100.000	3.69e-12	ACGAGCAGGCCGCCCACGTTGCCCAATGCG-GAGA
BreBifido_1-spacer61	BreCNCMI-4321ph1	100.000	3.69e-12	ACGAGCAGGCCGCCCACGTTGCCCAATGCG-GAGA
BreBifido_1-spacer61	Bre689bph1	100.000	3.69e-12	ACGAGCAGGCCGCCCACGTTGCCCAATGCG-GAGA
BreBifido_1-spacer61	Blon-gAF35-13ACph2	100.000	3.69e-12	ACGAGCAGGCCGCCCACGTTGCCCAATGCG-GAGA
BreBR-10-spacer17	BreCNCMI-4321ph1	100.000	3.21e-13	CCCACAGAACCGGCACGAGAGAACTCGAC-GAAGAAC
BreBR-10-spacer51	BreNRBB52ph2	100.000	3.69e-12	ATGAACGGCAGGTGGATCAC-GTCGGCGTCGGTGT
BreBR-10-spacer51	BreNRBB50ph1	100.000	3.69e-12	ATGAACGGCAGGTGGATCAC-GTCGGCGTCGGTGT
BreBR-10-spacer53	Bif85Bph2	100.000	1.09e-12	CAGGCAACGCAGGAAATCGACCCCGCTCTT-GCCGG
BreBR-10-spacer57	BifMGYG-HGUT-02396ph1	100.000	3.21e-13	AGTGAACCCGCATGG-CAGACCCCGCCGCCGTCGTAA
BreBR-10-spacer57	Bif85Bph2	100.000	3.21e-13	AGTGAACCCGCATGG-CAGACCCCGCCGCCGTCGTAA
BreBR-10-spacer65	Blong44Bph1	100.000	1.72e-10	ATGATACCGGCGTTGCGCAGGCTGGCCAGCA
BreBR-10-spacer73	BreDRBB28ph1	100.000	1.24e-11	ATGACAATGCTAGATATTCAGCCGAGCGC-TACA
BreBR-10-spacer93	BlongPC4ph1	100.000	3.69e-12	TCGTTGAGGTCGAGTATGCCCTGGTT-GAACTTGG
BreBR-10-spacer93	BlongLO-K29bph1	100.000	3.69e-12	TCGTTGAGGTCGAGTATGCCCTGGTT-

Spacer Name	Phage Name	Identity	E-value	Nucleotide Sequence
				GAACTTGG
BreBR-15-spacer105	BreNRBB52ph1	100.000	1.09e-12	AACGACCTCGACACCGGCAAGGTC-TACTACTGGAA
BreBR-15-spacer105	BreNRBB50ph1	100.000	1.09e-12	AACGACCTCGACACCGGCAAGGTC-TACTACTGGAA
BreBR-15-spacer111	BreDRBB28ph1	100.000	1.09e-12	TTGAAGGTGCCGGGACGGTTGCGCGG-CATGGGGCC
BreBR-15-spacer111	BreCNCMI-4321ph1	100.000	1.09e-12	TTGAAGGTGCCGGGACGGTTGCGCGG-CATGGGGCC
BreBR-15-spacer111	Bre689bph1	100.000	1.09e-12	TTGAAGGTGCCGGGACGGTTGCGCGG-CATGGGGCC
BreBR-15-spacer111	BlongAF36-1ph1	100.000	1.09e-12	TTGAAGGTGCCGGGACGGTTGCGCGG-CATGGGGCC
BreBR-15-spacer113	BreDRBB28ph1	100.000	3.69e-12	TTCGCATGGATGCGGCAGACCGGCCGGCG-CAATG
BreBR-15-spacer113	BreCNCMI-4321ph1	100.000	3.69e-12	TTCGCATGGATGCGGCAGACCGGCCGGCG-CAATG
BreBR-15-spacer115	BreDRBB28ph1	100.000	3.21e-13	GCTGTGGAGTCGAGCGGG-TACATGGTCGCTTCATGG
BreBR-15-spacer115	BreCNCMI-4321ph1	100.000	3.21e-13	GCTGTGGAGTCGAGCGGG-TACATGGTCGCTTCATGG
BreBR-15-spacer115	Bre689bph1	100.000	3.21e-13	GCTGTGGAGTCGAGCGGG-TACATGGTCGCTTCATGG
BreBR-15-spacer12	BlongMCC10094ph1	98.000	4.38e-19	CCCGATGTCTATTTAGAAGTACAACACCCTT-GTTGGTCCTGTAATTCTAA
BreBR-15-spacer125	BreBIO6018ph1	100.000	1.49e-11	GTCGGGCAGGGCGTT-GATGAGGCCCTGCGCCAG
BreBR-15-spacer14	BlongMCC10094ph1	100.000	3.77e-48	GCGTGCGGTAGCCGAGCACGCG-CATGGGGCGGTTGTTGATCTCGTCGAC-GATCTCCCTGACCTCCTTT-GCCATGTCCATCCGTATCTCGCAGCGTTTGGG
BreBR-15-spacer45	BlongMCC10102ph1	100.000	3.69e-12	TCCAGCTACCAGCAGTCGGTCGGCGG-CATCGACA
BreBR-15-spacer49	BlongN3A01ph1	100.000	3.69e-12	CACCACGACCTGCGCTCGCTGGCGGAC-GGTTACG
BreBR-15-spacer49	BlongMCC10102ph1	100.000	3.69e-12	CACCACGACCTGCGCTCGCTGGCGGAC-GGTTACG
BreBR-15-spacer49	BlongMCC10008ph1	100.000	3.69e-12	CACCACGACCTGCGCTCGCTGGCGGAC-GGTTACG
BreBR-15-spacer51	BreDRBB28ph4	100.000	1.24e-11	CAGGCCAAGGCCGGCAACGCCATCGGCAC-GCCC
BreBR-15-spacer51	BlongN3A01ph1	100.000	1.24e-11	CAGGCCAAGGCCGGCAACGCCATCGGCAC-GCCC
BreBR-15-spacer51	BlongMCC10124ph1	100.000	1.24e-11	CAGGCCAAGGCCGGCAACGCCATCGGCAC-GCCC
BreBR-15-spacer51	BlongMCC10102ph1	100.000	1.24e-11	CAGGCCAAGGCCGGCAACGCCATCGGCAC-GCCC
BreBR-15-spacer51	BlongMCC10052ph1	100.000	1.24e-11	CAGGCCAAGGCCGGCAACGCCATCGGCAC-GCCC
BreBR-15-spacer51	BlongMCC10008ph1	100.000	1.24e-11	CAGGCCAAGGCCGGCAACGCCATCGGCAC-GCCC
BreBR-15-spacer51	BlongE18ph4	100.000	1.24e-11	CAGGCCAAGGCCGGCAACGCCATCGGCAC-GCCC
BreBR-15-spacer51	BlongBB-79ph2	100.000	1.24e-11	CAGGCCAAGGCCGGCAACGCCATCGGCAC-GCCC
BreBR-15-spacer51	BlongAPC1504ph1	100.000	1.24e-11	CAGGCCAAGGCCGGCAACGCCATCGGCAC-

Spacer Name	Phage Name	Identity	E-value	Nucleotide Sequence
				GCCC
BreBR-15-spacer51	Blon-gAF34-9ACph1	100.000	1.24e-11	CAGGCCAAGGCCGGCAACGCCATCGGCAC-GCCC
BreBR-15-spacer53	BreDRBB28ph4	100.000	3.69e-12	GAAGTGGTGCATGCGGGCCCGTTGAC-GGACTGGG
BreBR-15-spacer93	BreDRBB30ph3	100.000	3.69e-12	ATCTGCGGGAAAATGCTCACATGAGCG-GAACCCA
BreBR-15-spacer93	BreCNCMI-4321ph3	100.000	3.69e-12	ATCTGCGGGAAAATGCTCACATGAGCG-GAACCCA
BreBR-15-spacer95	BreDRBB28ph1	100.000	1.24e-11	GGCATGGGTGAAATACGCGCAGAACAC-GCAAAT
BreBR-15-spacer95	BreCNCMI-4321ph1	100.000	1.24e-11	GGCATGGGTGAAATACGCGCAGAACAC-GCAAAT
BreBR-15-spacer95	Bre689bph1	100.000	1.24e-11	GGCATGGGTGAAATACGCGCAGAACAC-GCAAAT
BreBR-20-spacer58	BreDRBB28ph1	100.000	1.09e-12	CCGAGCGTGGTGTGACCGGCGTTGCG-CATGGCGAT
BreBR-20-spacer58	BreCNCMI-4321ph1	100.000	1.09e-12	CCGAGCGTGGTGTGACCGGCGTTGCG-CATGGCGAT
BreBR-20-spacer58	Bre689bph1	100.000	1.09e-12	CCGAGCGTGGTGTGACCGGCGTTGCG-CATGGCGAT
BreBR-20-spacer58	Blon-gAF35-13ACph2	100.000	1.09e-12	CCGAGCGTGGTGTGACCGGCGTTGCG-CATGGCGAT
BreBR-A29-spacer12	BreLMC520ph3	100.000	1.09e-12	CAGCCGTACACGTCGGGCGGCAC-GCGCTGGTCGGT
BreBR-A29-spacer12	BreBR3ph2	100.000	1.09e-12	CAGCCGTACACGTCGGGCGGCAC-GCGCTGGTCGGT
BreBR-A29-spacer12	Blong-CECT7210.2ph3	100.000	1.09e-12	CAGCCGTACACGTCGGGCGGCAC-GCGCTGGTCGGT
BreBR-A29-spacer12	Blong-CCUG30698ph3	100.000	1.09e-12	CAGCCGTACACGTCGGGCGGCAC-GCGCTGGTCGGT
BreBR-A29-spacer12	BlongAPC1462ph1	100.000	1.09e-12	CAGCCGTACACGTCGGGCGGCAC-GCGCTGGTCGGT
BreBR-A29-spacer12	Blong72Bph1	100.000	1.09e-12	CAGCCGTACACGTCGGGCGGCAC-GCGCTGGTCGGT
BreBR-A29-spacer12	Blong157Fph1	100.000	1.09e-12	CAGCCGTACACGTCGGGCGGCAC-GCGCTGGTCGGT
BreBR-A29-spacer121	BreUMB0089ph2	100.000	1.09e-12	GAGGCGTCGGCGGAGACATGCGCATGG-TATAAGCC
BreBR-A29-spacer121	Blon-gATCC15697ph3	100.000	5.07e-11	GCGTCGGCGGAGACATGCGCATGGTATAAGCC
BreBR-A29-spacer121	Blon-gATCC15697.2ph3	100.000	5.07e-11	GCGTCGGCGGAGACATGCGCATGGTATAAGCC
BreBR-A29-spacer14	BreNRBB52ph5	100.000	1.24e-11	CACCGCATGCGAGATCGCGCCGGCACCCAC-GGC
BreBR-A29-spacer14	BreMCC1605ph1	100.000	1.24e-11	CACCGCATGCGAGATCGCGCCGGCACCCAC-GGC
BreBR-A29-spacer14	BreLMC520ph3	100.000	1.24e-11	CACCGCATGCGAGATCGCGCCGGCACCCAC-GGC
BreBR-A29-spacer14	BreDRBB28ph5	100.000	1.24e-11	CACCGCATGCGAGATCGCGCCGGCACCCAC-GGC
BreBR-A29-spacer14	BreDRBB26ph1	100.000	1.24e-11	CACCGCATGCGAGATCGCGCCGGCACCCAC-GGC
BreBR-A29-spacer14	BreBR3ph2	100.000	1.24e-11	CACCGCATGCGAGATCGCGCCGGCACCCAC-GGC
BreBR-A29-spacer14	Bre082W48ph1	100.000	1.24e-11	CACCGCATGCGAGATCGCGCCGGCACCCAC-

Spacer Name	Phage Name	Identity	E-value	Nucleotide Sequence
				GGC
BreBR-A29-spacer14	Bre017W439ph2	100.000	1.24e-11	CACCGCATGCGAGATCGCGCCGGCACCCAC-GGC
BreBR-A29-spacer14	BlongLO-K29bph1	100.000	1.24e-11	CACCGCATGCGAGATCGCGCCGGCACCCAC-GGC
BreBR-A29-spacer14	Blong-CECT7210.2ph3	100.000	1.24e-11	CACCGCATGCGAGATCGCGCCGGCACCCAC-GGC
BreBR-A29-spacer14	Blong-CCUG30698ph3	100.000	1.24e-11	CACCGCATGCGAGATCGCGCCGGCACCCAC-GGC
BreBR-A29-spacer14	Blong157Fph1	100.000	1.24e-11	CACCGCATGCGAGATCGCGCCGGCACCCAC-GGC
BreBR-A29-spacer163	BreNRBB52ph5	100.000	1.24e-11	CGACGA-GATGGCCGACCCCGAACTCGAATCGTT
BreBR-A29-spacer163	BreMCC1605ph1	100.000	1.24e-11	CGACGA-GATGGCCGACCCCGAACTCGAATCGTT
BreBR-A29-spacer163	BreLMC520ph3	100.000	1.24e-11	CGACGA-GATGGCCGACCCCGAACTCGAATCGTT
BreBR-A29-spacer163	BreJR01ph1	100.000	1.24e-11	CGACGA-GATGGCCGACCCCGAACTCGAATCGTT
BreBR-A29-spacer163	BreDRBB28ph5	100.000	1.24e-11	CGACGA-GATGGCCGACCCCGAACTCGAATCGTT
BreBR-A29-spacer163	BreDRBB26ph1	100.000	1.24e-11	CGACGA-GATGGCCGACCCCGAACTCGAATCGTT
BreBR-A29-spacer163	BreBR3ph2	100.000	1.24e-11	CGACGA-GATGGCCGACCCCGAACTCGAATCGTT
BreBR-A29-spacer163	BlongLO-K29bph1	100.000	1.24e-11	CGACGA-GATGGCCGACCCCGAACTCGAATCGTT
BreBR-A29-spacer163	Blong-CECT7210.2ph3	100.000	1.24e-11	CGACGA-GATGGCCGACCCCGAACTCGAATCGTT
BreBR-A29-spacer163	Blong-CCUG30698ph3	100.000	1.24e-11	CGACGA-GATGGCCGACCCCGAACTCGAATCGTT
BreBR-A29-spacer163	BlongAPC1462ph1	100.000	1.24e-11	CGACGA-GATGGCCGACCCCGAACTCGAATCGTT
BreBR-A29-spacer163	Blong72Bph1	100.000	1.24e-11	CGACGA-GATGGCCGACCCCGAACTCGAATCGTT
BreBR-A29-spacer163	Blong157Fph1	100.000	1.24e-11	CGACGA-GATGGCCGACCCCGAACTCGAATCGTT
BreBR-A29-spacer2	BlongBifido_04ph1	100.000	3.21e-13	ACCCAGTCCATGAC-GTTCTCGAACCCTCGCGGCGAA
BreBR-A29-spacer38	BreDRBB28ph1	100.000	1.24e-11	ATCGGCACGATAGGCCATGGCGGCAC-CGTCACC
BreBR-A29-spacer38	BreCNCMI-4321ph1	100.000	1.24e-11	ATCGGCACGATAGGCCATGGCGGCAC-CGTCACC
BreBR-A29-spacer38	Bre689bph1	100.000	1.24e-11	ATCGGCACGATAGGCCATGGCGGCAC-CGTCACC
BreBR-A29-spacer46	BreDRBB28ph1	100.000	3.69e-12	TCCTTGACTAGATGGATTGGTTAACAATT-GATTG
BreBR-A29-spacer46	BreCNCMI-4321ph1	100.000	3.69e-12	TCCTTGACTAGATGGATTGGTTAACAATT-GATTG
BreBR-A29-spacer6	BlongBG7ph1	100.000	3.69e-12	CGCGCCGATGGTCGCCGCGCCTCGGTCGATGACG
BreBR-A29-spacer8	BlongBG7ph1	100.000	1.33e-11	GCCTCCAGCGACCGGAAGCCGCTCGGCAC-CGTC
BreBR-I29-spacer12	BreDRBB28ph1	100.000	3.69e-12	TGGCGCAATGGGCGTTGAAC-GTCGCCATGAACGC
BreBR-I29-spacer12	BreCNCMI-4321ph	100.000	3.69e-12	TGGCGCAATGGGCGTTGAAC-

Spacer Name	Phage Name	Identity	E-value	Nucleotide Sequence
	1			GTCGCCATGAACGC
BreBR-I29-spacer12	Bre689bph1	100.000	3.69e-12	TGGCGCAATGGGCGTTGAAC-GTCGCCATGAACGC
BreBR-I29-spacer12	Blon-gAF35-13ACph2	100.000	3.69e-12	TGGCGCAATGGGCGTTGAAC-GTCGCCATGAACGC
BreBR-I29-spacer20	Brelw01ph1	100.000	3.69e-12	GCGGTCGTGGAATACGCAGACAA-GAAACATTTCA
BreBR-I29-spacer20	BlongNCTC11817ph3	100.000	3.69e-12	GCGGTCGTGGAATACGCAGACAA-GAAACATTTCA
BreBR-I29-spacer61	BlongBifido_04ph1	100.000	1.09e-12	GCGAAGAAGACGGCTGGCAATGCG-CAGACTTTGGC
BreCECT7263-spacer103	BreDRBB28ph1	100.000	2.76e-14	TCGCGGTGTTGAAGACCGTCACATGGGCG-CAACTGTCA
BreCECT7263-spacer103	BreCNCMI-4321ph1	100.000	2.76e-14	TCGCGGTGTTGAAGACCGTCACATGGGCG-CAACTGTCA
BreCECT7263-spacer103	Bre689bph1	100.000	2.76e-14	TCGCGGTGTTGAAGACCGTCACATGGGCG-CAACTGTCA
BreCECT7263-spacer113	BlongMCC10079ph1	100.000	4.47e-11	TGCCACGGGTAGAGATGCACGTGGAGGAACTT
BreCECT7263-spacer121	BreNRBB50ph1	100.000	1.09e-12	GGGTTGCCGTAGGTGCCGCCGCCCTGA-TAGCCGTT
BreCECT7263-spacer121	BlongF8ph1	100.000	1.09e-12	GGGTTGCCGTAGGTGCCGCCGCCCTGA-TAGCCGTT
BreCECT7263-spacer99	BreDRBB30ph3	100.000	3.69e-12	CTTGAGGATGAATCCAGTCAGGCGATGCTG-GACG
BreCECT7263-spacer99	BreCNCMI-4321ph3	100.000	3.69e-12	CTTGAGGATGAATCCAGTCAGGCGATGCTG-GACG
BreJCM7019-spacer52	BifS17ph1	100.000	1.09e-12	GCGCCCAACTGCTCATCAATCTGATGGACTG-CATC
BreJCM7019-spacer72	BreNRBB01ph2	100.000	1.24e-11	GGACGGTTCCAATCCGAGCGCGCTG-CAGGTCAT
BreJCM7019-spacer72	BreDSM20213.3ph2	100.000	1.24e-11	GGACGGTTCCAATCCGAGCGCGCTG-CAGGTCAT
Brelw01-spacer75	Bre689bph1	100.000	3.21e-13	GGCCGACCACAAGGCCACCGGCAG-CATGGACGCGGA
BreMC1-spacer6	BreNRBB51ph1	100.000	2.70e-21	CACTACGAGGAGTCGTTCAAGCGG-CAGATCGTGCAGTTGTATGAGAACGGC
BreMC1-spacer6	BreNRBB56ph3	98.039	1.26e-19	CACTACGAGGAGTCGTTCAAGCGG-CAGATCGTGCAGTTGTATGAGAACGGC
BreMC1-spacer6	BadBB23ph1	98.039	1.26e-19	CACTACGAGGAGTCGTTCAAGCGG-CAGATCGTGCAGTTGTATGAGAACGGC
BreMC1-spacer7	BreNRBB51ph1	100.000	2.80e-16	GAGCCGGCGCGCGAGATCAGGGCCGAGTAC-GACATCTCGCAT
BreMC1-spacer7	BreNRBB56ph3	100.000	1.01e-15	AGCCGGCGCGCGAGATCAGGGCCGAGTAC-GACATCTCGCAT
BreMC1-spacer7	BreNRBB56ph3	100.000	1.01e-15	AGCCGGCGCGCGAGATCAGGGCCGAGTAC-GACATCTCGCAT
BreMC1-spacer8	BreNRBB56ph3	100.000	2.70e-21	CACTACGAGGAGTCGTTCAAACGG-CAGATCGTGCAGTTGTATGAGAACGGC
BreMC1-spacer8	BreNRBB51ph1	98.039	1.26e-19	CACTACGAGGAGTCGTTCAAACGG-CAGATCGTGCAGTTGTATGAGAACGGC
BreMCC1604-spacer12	BreDRBB28ph1	100.000	1.09e-12	GTTGAGTGTCTTATCGATGAATTGCCTG-TAGACGC
BreMCC1604-spacer12	BreCNCMI-4321ph1	100.000	1.09e-12	GTTGAGTGTCTTATCGATGAATTGCCTG-TAGACGC
BreMCC1604-spacer	Bre689bph1	100.000	1.09e-12	GTTGAGTGTCTTATCGATGAATTGCCTG-

Spacer Name	Phage Name	Identity	E-value	Nucleotide Sequence
12				TAGACGC
BreMCC1604-spacer 12	Blon-gAF35-13ACph2	100.000	1.09e-12	GTTGAGTGTCTTATCGATGAATTGCCTG-TAGACGC
BreMCC1604-spacer 42	Bad1-11ph1	100.000	3.69e-12	TACAATGCCGTGAACAGCCTCTAAAA-GCATTAAG
BreNCFB2258-spacer 35	BifS17ph1	100.000	3.69e-12	GCGGCTGGGAGACCGTGCCGAACCG-CAGGTCGGG
BreNCFB2258-spacer 35	BifMGYG-HGUT-02396ph2	100.000	3.69e-12	GCGGCTGGGAGACCGTGCCGAACCG-CAGGTCGGG
BreNRBB09-spacer100	BlongEK5ph1	100.000	3.69e-12	TGACGCAATCACGAGTCGTGGATGCCGTT-GACCT
BreNRBB09-spacer102	BlongEK5ph1	100.000	1.24e-11	CTGGTCGATGCCGTCGGTGCCCCACTGGATGCT
BreNRBB09-spacer104	BlongTM01-1ph1	100.000	3.69e-12	TGATGGGTTCGGCGTTCGCGCTCACCCAG-TCGCT
BreNRBB09-spacer104	BlongMCC10101ph1	100.000	3.69e-12	TGATGGGTTCGGCGTTCGCGCTCACCCAG-TCGCT
BreNRBB09-spacer104	BlongGT15ph3	100.000	3.69e-12	TGATGGGTTCGGCGTTCGCGCTCACCCAG-TCGCT
BreNRBB09-spacer104	BlongDS15_3ph1	100.000	3.69e-12	TGATGGGTTCGGCGTTCGCGCTCACCCAG-TCGCT
BreNRBB09-spacer104	BlongBB-79ph1	100.000	3.69e-12	TGATGGGTTCGGCGTTCGCGCTCACCCAG-TCGCT
BreNRBB09-spacer15	BreBR3ph3	98.305	1.09e-23	GACAC-CGAGCTTTTAATGCTCGTCGCGGTCTGCTCCACGCTGCTCTTGGTCGCGTACGT
BreNRBB09-spacer36	BreDRBB28ph1	100.000	1.11e-08	CCGGGCGCTATCGTGGTGGCGATGTCGGA
BreNRBB09-spacer36	BreCNCMI-4321ph1	100.000	1.11e-08	CCGGGCGCTATCGTGGTGGCGATGTCGGA
BreNRBB09-spacer36	Bre689bph1	100.000	1.11e-08	CCGGGCGCTATCGTGGTGGCGATGTCGGA
BreNRBB09-spacer36	Blon-gAF35-13ACph2	100.000	1.11e-08	CCGGGCGCTATCGTGGTGGCGATGTCGGA
BreNRBB09-spacer44	BreDRBB30ph3	100.000	1.72e-10	CCGACACATTGTCCAATTTCCCGTTCTCGTC
BreNRBB09-spacer44	BreCNCMI-4321ph3	100.000	1.72e-10	CCGACACATTGTCCAATTTCCCGTTCTCGTC
BreNRBB09-spacer96	BreDRBB28ph1	100.000	3.21e-13	GCCATCATGGACTTGGGCATGAGCGAC-GTCGCCAAG
BreNRBB09-spacer96	BreCNCMI-4321ph1	100.000	3.21e-13	GCCATCATGGACTTGGGCATGAGCGAC-GTCGCCAAG
BreNRBB09-spacer96	Bre689bph1	100.000	3.21e-13	GCCATCATGGACTTGGGCATGAGCGAC-GTCGCCAAG
BreNRBB09-spacer96	Blon-gAF35-13ACph2	100.000	3.21e-13	GCCATCATGGACTTGGGCATGAGCGAC-GTCGCCAAG
BreNRBB11-spacer116	BreMCC1605ph1	100.000	3.69e-12	CGCTGCTGCTACCCAGCAGGCGGGCGA-TATCCTC
BreNRBB11-spacer116	BreJR01ph1	100.000	3.69e-12	CGCTGCTGCTACCCAGCAGGCGGGCGA-TATCCTC
BreNRBB11-spacer116	BreDRBB28ph5	100.000	3.69e-12	CGCTGCTGCTACCCAGCAGGCGGGCGA-TATCCTC
BreNRBB11-spacer116	BreDRBB26ph1	100.000	3.69e-12	CGCTGCTGCTACCCAGCAGGCGGGCGA-TATCCTC
BreNRBB11-spacer116	BreBR3ph2	100.000	3.69e-12	CGCTGCTGCTACCCAGCAGGCGGGCGA-TATCCTC
BreNRBB11-spacer116	Bre082W48ph1	100.000	3.69e-12	CGCTGCTGCTACCCAGCAGGCGGGCGA-TATCCTC
BreNRBB11-spacer11	Blong72Bph1	100.000	3.69e-12	CGCTGCTGCTACCCAGCAGGCGGGCGA-

Spacer Name	Phage Name	Identity	E-value	Nucleotide Sequence
6				TATCCTC
BreNRBB11-spacer116	Blong157Fph1	100.000	3.69e-12	CGCTGCTGCTACCCAGCAGGCGGGCGA-TATCCTC
BreNRBB11-spacer120	BlongMCC10116ph1	100.000	3.69e-12	CGAACATCAAGGGTCTGCTCTCCCGCGACATCCA
BreNRBB11-spacer120	BlongMCC10111ph1	100.000	3.69e-12	CGAACATCAAGGGTCTGCTCTCCCGCGACATCCA
BreNRBB11-spacer120	BlongMCC10100ph1	100.000	3.69e-12	CGAACATCAAGGGTCTGCTCTCCCGCGACATCCA
BreNRBB11-spacer120	BlongMCC10093ph1	100.000	3.69e-12	CGAACATCAAGGGTCTGCTCTCCCGCGACATCCA
BreNRBB11-spacer120	BlongMCC10079ph1	100.000	3.69e-12	CGAACATCAAGGGTCTGCTCTCCCGCGACATCCA
BreNRBB11-spacer120	BlongMCC10077ph1	100.000	3.69e-12	CGAACATCAAGGGTCTGCTCTCCCGCGACATCCA
BreNRBB11-spacer120	BlongMCC10073ph1	100.000	3.69e-12	CGAACATCAAGGGTCTGCTCTCCCGCGACATCCA
BreNRBB11-spacer120	BlongMCC10064ph1	100.000	3.69e-12	CGAACATCAAGGGTCTGCTCTCCCGCGACATCCA
BreNRBB11-spacer120	BlongMCC10044ph1	100.000	3.69e-12	CGAACATCAAGGGTCTGCTCTCCCGCGACATCCA
BreNRBB11-spacer120	BlongMCC10038ph1	100.000	3.69e-12	CGAACATCAAGGGTCTGCTCTCCCGCGACATCCA
BreNRBB11-spacer120	BlongLO-K29aph1	100.000	3.69e-12	CGAACATCAAGGGTCTGCTCTCCCGCGACATCCA
BreNRBB11-spacer142	BreDRBB28ph1	100.000	4.47e-11	TCGCTCATGCCCAAGTCCATGATGGCCTTGTT
BreNRBB11-spacer142	BreCNCMI-4321ph1	100.000	4.47e-11	TCGCTCATGCCCAAGTCCATGATGGCCTTGTT
BreNRBB11-spacer142	Bre689bph1	100.000	4.47e-11	TCGCTCATGCCCAAGTCCATGATGGCCTTGTT
BreNRBB11-spacer142	Blon-gAF35-13ACph2	100.000	4.47e-11	TCGCTCATGCCCAAGTCCATGATGGCCTTGTT
BreNRBB11-spacer38	BifS17ph1	100.000	3.21e-13	CATCTGCCGTCCTGCGGTGTCCTCGCTCAGGTAGGC
BreNRBB11-spacer38	BifMGYG-HGUT-02396ph2	100.000	3.21e-13	CATCTGCCGTCCTGCGGTGTCCTCGCTCAGGTAGGC
BreNRBB51-spacer102	BreDRBB28ph1	100.000	1.09e-12	TTCCATGGTCGGGTTCCTTTCTGTCAGGCGTT-GGC
BreNRBB51-spacer102	BreCNCMI-4321ph1	100.000	1.09e-12	TTCCATGGTCGGGTTCCTTTCTGTCAGGCGTT-GGC
BreNRBB51-spacer102	Bre689bph1	100.000	1.09e-12	TTCCATGGTCGGGTTCCTTTCTGTCAGGCGTT-GGC
BreNRBB51-spacer102	BlongAF36-1ph1	100.000	1.09e-12	TTCCATGGTCGGGTTCCTTTCTGTCAGGCGTT-GGC
BreNRBB51-spacer102	Blon-gAF35-13ACph2	100.000	1.09e-12	TTCCATGGTCGGGTTCCTTTCTGTCAGGCGTT-GGC
BreNRBB51-spacer122	BreDRBB30ph3	100.000	3.21e-13	CGGCCAGTCGATCAGCTGGCTCCTGAG-CAAGGCGGA
BreNRBB51-spacer122	BreCNCMI-4321ph3	100.000	3.21×10^{-13}	CGGCCAGTCGATCAGCTGGCTCCTGAG-CAAGGCGGA
BreNRBB51-spacer122	BreBIO6018ph1	100.000	3.21e-13	CGGCCAGTCGATCAGCTGGCTCCTGAG-CAAGGCGGA
BreNRBB51-spacer124	BreDRBB28ph1	100.000	1.09e-12	CCGACGCCATCCCCGGCGCTTCGGG-CAAACTCCAG
BreNRBB51-spacer12	BreCNCMI-4321ph	100.000	1.09e-12	CCGACGCCATCCCCGGCGCTTCGGG-

Spacer Name	Phage Name	Identity	E-value	Nucleotide Sequence
4	1			CAAACTCCAG
BreNRBB51-spacer124	Bre689bph1	100.000	1.09e-12	CCGACGCCATCCCCGGCGCTTCGGG-CAAACTCCAG
BreNRBB51-spacer124	Blon-gAF35-13ACph2	100.000	1.09e-12	CCGACGCCATCCCCGGCGCTTCGGG-CAAACTCCAG
BreNRBB51-spacer136	BreDRBB28ph1	100.000	3.69e-12	AACACGACAACTAAAAGGACACAACAATGG-CATT
BreNRBB51-spacer136	BreCNCMI-4321ph1	100.000	3.69e-12	AACACGACAACTAAAAGGACACAACAATGG-CATT
BreNRBB51-spacer136	Bre689bph1	100.000	3.69e-12	AACACGACAACTAAAAGGACACAACAATGG-CATT
BreNRBB51-spacer138	Bre689bph1	100.000	3.69e-12	CAACCAATTGTTAACCAATCCATCTAG-TCAAGGA
BreNRBB51-spacer140	Bif85Bph2	100.000	3.69e-12	CGGCCAAAAGGCCGATGGCCTT-GGTGATGGGGTC
BreNRBB51-spacer144	Bif85Bph2	100.000	1.09e-12	GCGCAGACGGGCAAGGACGCGGGCGACCAC-TTCAA
BreNRBB51-spacer148	BreDRBB28ph1	100.000	1.09e-12	ATGAGCGACAGCACCGACAAGTTCGTG-CAGACCAT
BreNRBB51-spacer148	BreCNCMI-4321ph1	100.000	1.09e-12	ATGAGCGACAGCACCGACAAGTTCGTG-CAGACCAT
BreNRBB51-spacer148	Bre689bph1	100.000	1.09e-12	ATGAGCGACAGCACCGACAAGTTCGTG-CAGACCAT
BreNRBB51-spacer148	Blon-gAF35-13ACph2	100.000	1.09e-12	ATGAGCGACAGCACCGACAAGTTCGTG-CAGACCAT
BreNRBB51-spacer150	BreDRBB28ph1	100.000	3.69e-12	ACCAAACCCAAGGCCGTGCTCG-TAACCCGCGTCA
BreNRBB51-spacer150	BreCNCMI-4321ph1	100.000	3.69e-12	ACCAAACCCAAGGCCGTGCTCG-TAACCCGCGTCA
BreNRBB51-spacer150	Bre689bph1	100.000	3.69e-12	ACCAAACCCAAGGCCGTGCTCG-TAACCCGCGTCA
BreNRBB52-spacer137	Bre689bph1	100.000	3.69e-12	GCATGAGCAACGGCAGCATGTGCCTCACGTT-GAA
BreNRBB52-spacer137	BlongAF36-1ph1	100.000	3.69e-12	GCATGAGCAACGGCAGCATGTGCCTCACGTT-GAA
BreNRBB52-spacer137	Blon-gAF35-13ACph2	100.000	3.69e-12	GCATGAGCAACGGCAGCATGTGCCTCACGTT-GAA
BreNRBB56-spacer102	BreUMB0089ph2	100.000	3.69e-12	CTCGCTTTCCGCCGCCATGATCGCGTCGGAC-GAG
BreNRBB56-spacer102	BlongMCC10130ph1	100.000	1.33e-11	TCGCTTTCCGCCGCCATGATCGCGTCGGACGAG
BreNRBB56-spacer98	BreUMB0089ph2	100.000	1.09e-12	CGCACAC-GGGCCGCGCTGGCCAATGTGGTCTGCAT
BreNRBB56-spacer98	BreDRBB27ph4	100.000	1.09e-12	CGCACAC-GGGCCGCGCTGGCCAATGTGGTCTGCAT
BreNRBB56-spacer98	BlongBifido_04ph1	100.000	1.09e-12	CGCACAC-GGGCCGCGCTGGCCAATGTGGTCTGCAT
BreNRBB56-spacer98	Blon-gATCC15697ph3	100.000	1.09e-12	CGCACAC-GGGCCGCGCTGGCCAATGTGGTCTGCAT
BreNRBB56-spacer98	Blon-gATCC15697.2ph3	100.000	1.09e-12	CGCACAC-GGGCCGCGCTGGCCAATGTGGTCTGCAT
